# Assembly of Lanthanide-Containing Tungstotellurates(VI): Syntheses, Structures, and Catalytic Properties

**DOI:** 10.3389/fchem.2020.598961

**Published:** 2020-11-23

**Authors:** Jie Li, Shuxia Shang, Zhengguo Lin, Zishuo Yao, Ni Zhen, Zhen Li, Yingnan Chi, Changwen Hu

**Affiliations:** ^1^Key Laboratory of Cluster Science Ministry of Education, Beijing Key Laboratory of Photoelectronic/Electrophotonic Conversion Materials, School of Chemistry and Chemical Engineering, Beijing Institute of Technology, Beijing, China; ^2^China Resources Double-Crane Pharmaceutical Co., Ltd., Beijing, China

**Keywords:** polyoxometalates, tungstotellurates(VI), lanthanide ions, self-assembly, Lewis acidic catalysis, cyanosilylation

## Abstract

Lanthanide (Ln)-containing polyoxometalates (POMs) have attracted particular attention owing to their structural diversity and potential applications in luminescence, magnetism, and catalysis. Herein three types of Ln-containing tungstotellurates(VI) (Ln = Dy^3+^, Ho^3+^, Er^3+^, Tm^3+^, Yb^3+^, and Lu^3+^), dimeric (DMAH)_*n*_[H_22−*n*_{Ln(H_2_O)_3_[TeW_17_O_61_]}_2_]^·^mH_2_O (abbreviated as {Ln_2_Te_2_W_34_}; DMAH^+^ = dimethylammonium), mono-substituted (DMAH)_7_Na_2_{H_2_Ln(H_2_O)_4_[TeW_17_O_61_]}^·^mH_2_O (abbreviated as {LnTeW_17_}), and three-dimensional (3D) inorganic frameworks (DMAH)_*n*_{H_3−*n*_Ln(H_2_O)_4_[TeW_6_O_24_]}^·^mH_2_O (abbreviated as {LnTeW_6_}), have been synthesized by using simple metal salts and characterized by single-crystal X-ray diffraction and other routine techniques. Interestingly, the assembly of these POMs is pH dependent. Using the same starting materials, {Ln_2_Te_2_W_34_} were obtained at pH 1.7, where two Dawson-like monovacant [TeW_17_O_61_]^14−^ are linked by two Ln^3+^ ions; mono-substituted Dawson-like {LnTeW_17_} were isolated at pH 1.9, and 3D inorganic framework {LnTeW_6_} based on Anderson-type [TeW_6_O_24_]^6−^ were formed at pH 2.3. It was also found that the assembly of Ln-containing POMs depends on the type of Ln^3+^ ions. The three types of POMs can be prepared by using Ln^3+^ ions with a relatively smaller ionic radius, such as Tb^3+^-Lu^3+^, while the use of Ln^3+^ ions (La^3+^-Eu^3+^) results in the formation of precipitation or {TeW_18_O_62_} clusters. Furthermore, three {LnTeW_6_} (Ln = Tb^3+^, Er^3+^, Lu^3+^) were used as Lewis acid catalysts for the cyanosilylation of benzaldehydes, and their catalytic activity decreases with the decrease of Ln^3+^ ionic radius, giving the order: {TbTeW_6_} > {ErTeW_6_} > {LuTeW_6_}. Notably, {TbTeW_6_} is stable to leaching and can be reused for five cycles without a significant loss of its activity.

## Introduction

Polyoxometalates (POMs) are a unique class of metal-oxo clusters with tunable structures and excellent properties (Hill, 1998; Cronin and Müller, 2012). Due to the existence of abundant surface oxygen atoms, POMs as inorganic ligands can easily coordinate with transition metal or lanthanide (Ln) ions, resulting in the formation of discrete nanoscale clusters or extended structures. Among them, Ln-containing polyoxotungstates have attracted numerous attention owing to their structural diversity (Ma et al., 2015; Zhao et al., 2016b) and attractive applications in luminescence (Granadeiro et al., 2010; Ritchie et al., 2010b), magnetism (Clemente-Juan et al., 2012; Suzuki et al., 2013), and catalysis (Boglio et al., 2006; Suzuki et al., 2014; Li et al., 2018). Up to now, two synthetic strategies have been developed to construct Ln-containing POMs. One is building block method, where different lacunary POM precursors, such as monolacunary [XW_11_O_39_]^*n*−^ (Zhang et al., 2012; Arab Fashapoyeh et al., 2018; Mougharbel et al., 2020) and trilacunary [XW_9_O_34_]^*n*−^ (X = P, Si, Ge) (Zhao et al., 2014, 2017) and [XW_9_O_33_]^9−^ (X = As^III^, Sb^III^) (Chen et al., 2018; Kaushik et al., 2018), were used to coordinate with Ln^3+^ ions. The other is one-pot synthetic strategy, by which intricate POM structures are fabricated through the condensation reaction of simple metal salts with heteroanions (Chen et al., 2013, 2014; Zhao et al., 2016a; Liu J. C. et al., 2018). Although the one-pot method is simple and straightforward, understanding the assembly process is challenging.

Generally, the assembly of Ln-containing POMs can be affected by many synthetic parameters, such as molar ratio of reactants, temperature, pH, solvents, and so on. Among them, the pH value is a key factor. For example, Chen et al. reported five Ce(III)-containing POMs, finding that the structural motif of POM building block can be influenced by the pH value (Chen et al., 2014). Subsequently, they also demonstrated that pH can control the arrangement of lacunary Keggin and Wells–Dawson clusters (Chen et al., 2019). Moreover, the type of Ln^3+^ ions plays a role during the formation of Ln-containing POMs. Ozeki and Yamase reported the effect of lanthanide contraction on the Ln–O bond lengths in [LnW_10_O_36_]^10−^ clusters (Ln = Pr, Nd, Sm, Gd, Tb, Dy) (Ozeki and Yamase, 1994). Previous investigations indicate that the arrangement of POM can also be influenced by the type of Ln^3+^ ions. For example, the assembly of monovacant [α-SiW_11_*O*_39_]^8^− with different Ln^3+^ ions leads to three different structural motifs: 1D linear chain [Yb(α-SiW_11_*O*_39_)(H_2_O)_2_]^5−^, 1D zigzag chain [Eu(α − *SiW*_11_*O*_39_)(H_2_O)_2_]^5−^, and 2D network [Nd_2_(α − *SiW*_11_*O*_39_)(H_2_O)_11_]^2−^ (Mialane et al., 2003). In 2007, Kortz et al. found that the configuration of POMs can change with the size of Ln^3+^ ions. For example, [Ln(β_2_ − *GeW*_11_*O*_39_)_2_]^13−^ was isolated when using a larger Ln^3+^ ion (Ln = La, Ce, Pr, Nd, Sm, Gd, or Dy), while [Ln(β_2_ − *GeW*_11_*O*_39_) (α − *GeW*_11_*O*_39_)]^13−^ was prepared when using a smaller Ln^3+^ ion (Ln = Ho, Er, or Tm) (Mougharbel et al., 2020).

Tungstotellurates are an important subfamily of polyoxotungstates with trigonal–pyramidal {Te^IV^O_3_}^2−^ or octahedral {Te^VI^O_6_}^6−^ as heteroanions. Due to the existence of lone pair electrons in Te(IV) atom, tungstotellurates(IV) tend to form lacunary structures, such as [H_2_Te_4_W_20_O_80_]^22−^ and [NaTeW_15_O_54_]^13−^ (Ismail et al., 2009), dimeric [Te_2_W_17_O_61_]^12−^, [Te_2_W_16_O_58_(OH)_2_]^14−^, and [Te_2_W_18_O_62_(OH)_2_]^10−^ (Ritchie et al., 2010a), as well as macrocyclic clusters: [W_28_Te_8_O_112_]^24−^, [W_28_Te_9_O_115_]^26−^, and [W_28_Te_10_O_118_]^28−^ (Gao et al., 2011). Therefore, Ln^3+^ ions can be readily incorporated into tungstotellurates(IV) (Chen et al., 2013, 2015; Han et al., 2017a,b; Liu J. L. et al., 2018; Liu et al., 2019; Zhang et al., 2020). In comparison, limited attention has been paid on tungstotellurates(VI). The first member of tungstotellurates(VI) is Anderson-type [TeW_6_O_24_]^6−^, which was first determined in 1986 (Schmidt et al., 1986). After that, until 2009 two new members, Dawson-like [H_3_W_18_O_56_(Te^VI^O_6_)]^7−^ ({TeW_18_O_62_}) and high nuclearity [H_10_${\text{Te}}_{2}^{\text{VI}}$W_58_O_198_]^26−^, were discovered by Cronin and Long (Yan et al., 2009). As the reported tungstotellurates(VI) are plenary clusters, Ln-containing tungstotellurates(VI) was nearly unexplored. Recently, Ln^3+^ ions (Ln = Eu, Gd, Tb) have been successfully introduced into tungstotellurates(VI) in our group, obtaining dimeric [H_10_(WO_2_){Ln(H_2_O)_5_(Te^VI^W_18_O_65_)}_2_]^12−^ (Ln = Eu, Gd, Tb) and tetrameric [H_16_{Ln(H_2_O)_5_(Te^VI^W_18_O_64_)}_4_]^28−^ (Ln = Eu, Gd), dimeric [H_6_{Tb(H_2_O)_3_(Te^VI^W_17_O_61_)}_2_]^12−^, mono-substituted [H_2_Tb(H_2_O)_4_(Te^VI^W_17_O_61_)]^9−^, and 3D inorganic framework [HTb(H_2_O)_4_{Te^VI^W_6_O_24_}]^2−^ (Shang et al., 2018). In the synthetic process, we find that the assembly of Tb-containing tungstotellurates(VI) is controlled by pH and that the formation of tetrameric clusters depends on the type of Ln^3+^ ions.

**Scheme 1:**
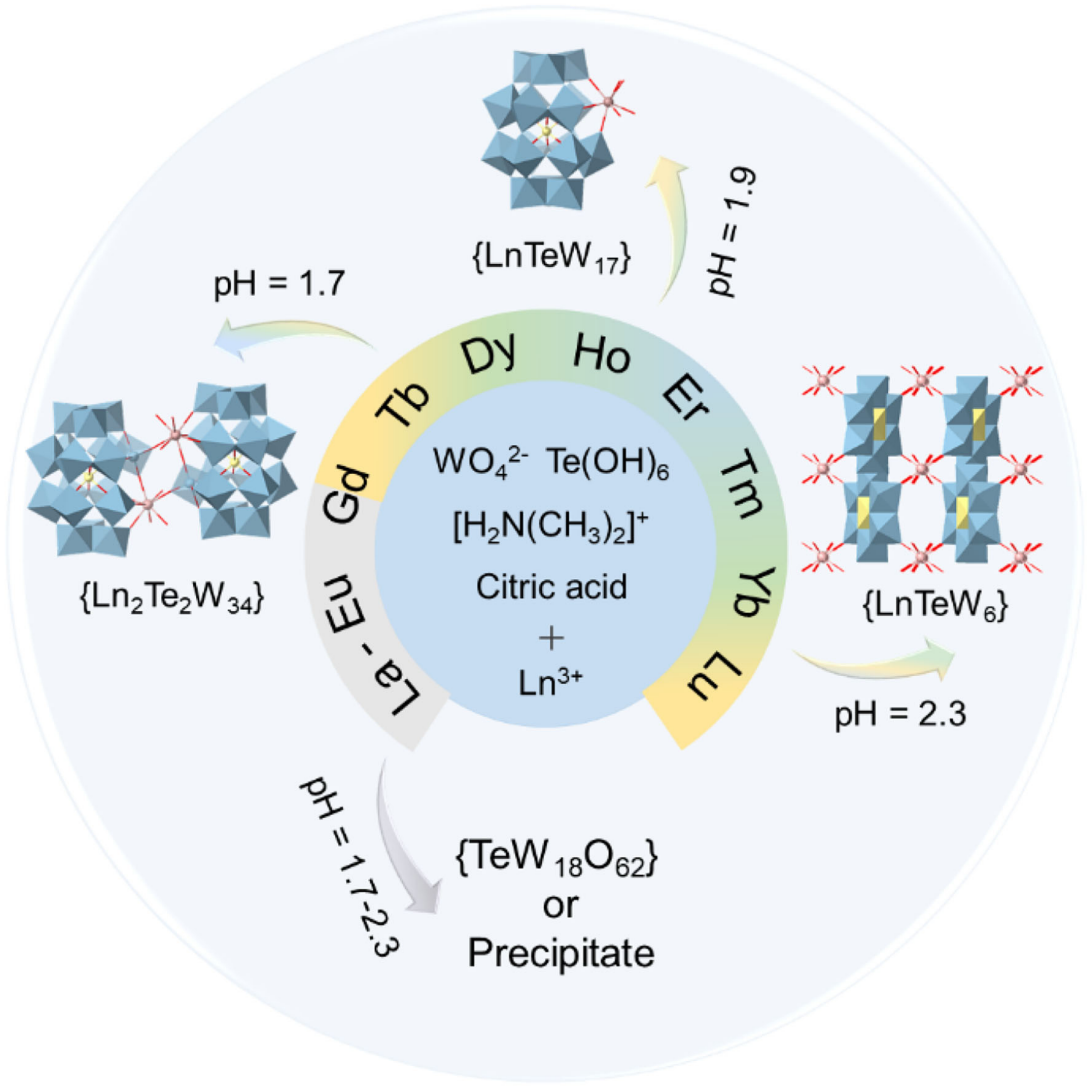
The synthetic illustration of the three types of Ln-containing tungstotellurates(VI): {Ln_2_Te_2_W_34_}, {LnTeW_17_}, and {LnTeW_6_}.

To systematically investigate the effect of Ln^3+^ ions on the assembly of Ln-containing tungstotellurates(VI), herein the Ln source was extended from La^3+^ to Lu^3+^ (except radioactive Pm^3+^). When Ln^3+^ (Tb^3+^, Dy^3+^, Ho^3+^, Er^3+^, Tm^3+^, Yb^3+^, Lu^3+^) with a relatively smaller ionic radius was used in the assembly, dimeric (DMAH)_n_[H_22−*n*_{Ln(H_2_O)_3_[TeW_17_O_61_]}_2_]^·^mH_2_O (abbreviated as {Ln_2_Te_2_W_34_}, DMAH^+^ = dimethylammonium), mono-substituted (DMAH)_7_Na_2_{H_2_Ln(H_2_O)_4_[TeW_17_O_61_]}^·^mH_2_O (abbreviated as {LnTeW_17_}), and 3D inorganic frameworks (DMAH)_n_{H_3−*n*_Ln(H_2_O)_4_[TeW_6_O_24_]}^·^mH_2_O (abbreviated as {LnTeW_6_}) were formed at pH 1.7, 1.9, and 2.3, respectively. However, under the otherwise identical conditions, no crystal compounds were generated by using La^3+^-Eu^3+^ with a relatively larger ionic radius (Scheme 1). The three types of Ln-containing tungstotellurates(VI) have been characterized by single-crystal X-ray diffraction, Fourier-transform infrared (FT-IR) spectra, elemental analyses, and TG analyses. Moreover, {LnTeW_6_} (Ln = Tb^3+^, Er^3+^, Lu^3+^) were used as Lewis acid catalysts to catalyze the cyanosilylation of aldehydes or ketone with trimethylsilylcyanide (TMSCN) under solvent-free conditions, and the relationship between the radii of Ln^3+^ ions and catalytic activity was also investigated.

## Experimental

### Materials

Most of the chemicals were purchased from the following manufacturers: Aladdin (PrCl_3_, 99.9%; TbCl_3_·6H_2_O, 99.9%; HoCl_3_·6H_2_O, 99.9%; LuCl_3_·6H_2_O, 99.9%; citric acid monohydrate, 99.5%; trimethylsilycyanide, 96%), Energy chemical [NdCl_3_·6H_2_O, 99.99%; SmCl_3_·6H_2_O, 99.99%; EuCl_3_·6H_2_O, 99.99%; GdCl_3_·6H_2_O, 99.99%; DyCl_3_·6H_2_O, 99.99%; ErCl_3_·6H_2_O, 99.99%; TmCl_3_·6H_2_O, 99.99%; dimethylamine hydrochloride, 98%; α-(trimethylsilyloxy)phenylacetonitrile, 97%], Alfa Aesar (YbCl_3_·6H_2_O, 99.9%), and Sinopharm Chemical Reagent Co., Ltd. (Na_2_WO_4_·2H_2_O, AR; LaCl_3_·nH_2_O, AR; CeCl_3_·6H_2_O, 99%; benzaldehyde, AR; acetophenone, AR). These reagents were used without further purification, except benzaldehyde. The Anderson-type tungstotellurates(VI), Na_6_TeW_6_O_24_·22H_2_O, was prepared according to the literature (Shah et al., 2014).

### Characterizations

FT-IR spectra were recorded on a Thermo IS5 spectrophotometer in the 4,000–400 cm^−1^ region as KBr-pressed pellets. Thermogravimetric analyses were carried out under flowing N_2_ on a Shimadzu DTG-60H instrument at a heating rate of 10°C/min. Elemental analyses (C, H, and N) were performed on ELEMENTAR vario EL cube Elmer CHN elemental analyzer. The Ln, W, Na, and Te elements were measured with ThermoiCAP Q mass spectrometry. The morphologies of {TbTeW_6_} were observed on a JEOL model S-4800 field-emission scanning electron microscopy with an accelerating voltage of 5 kV. The catalytic reaction was monitored on a Shimadzu GC-2014C instrument with a flame ionization detector.

### X-ray Crystallography

The X-ray single crystal diffraction data were collected on a Bruker APEX-II CCD diffractometer with graphite monochromatic Mo–Kα radiation (λ = 0.71073 Å) at 296 K. All crystals were sealed in capillary glass tubes for testing. All structures were solved using an intrinsic phasing method (SHELXT) (Sheldrick, 2015) and refined by full-matrix least-squares against $F_{\text{o}}^{2}$ with SHELXL software package (Sheldrick, 2015). Moreover, the residual disordered or crystal solvent molecules and cations were estimated by using the solvent MASK routine of OLEX2 (similar to PLATON/SQUEEZE) (Dolomanov et al., 2009). The hydrogen atoms were not incorporated in the refinements, and all non-hydrogen (Ln, W, Te, C, N, and O) atoms were refined anisotropically. The lattice H_2_O molecules and cations can be partly found from the Fourier maps, but not all lattice H_2_O molecules and cations can be found from the weak residual electron peaks. Thus, the numbers of the cations and lattice H_2_O molecules were determined and added to the molecular formula directly on the basis of elemental analyses, TG analyses, and the charge balance consideration. The crystallographic data for these crystal compounds are summarized in Supplementary Table 1. The crystallographic data have been deposited with the Cambridge Crystallographic Data Center with CCDC 2023272-2023277 for {Ln_2_Te_2_W_34_}, 2023278-2023283 for {LnTeW_17_}, and 2023284-2023289 for {LnTeW_6_} (Ln = Dy^3+^, Ho^3+^, Er^3+^, Tm^3+^, Yb^3+^, and Lu^3+^), respectively.

Synthesis of (DMAH)_*n*_[H_22−*n*_{Ln(H_2_O)_3_[TeW_17_O_61_]}_2_]^·^ mH_2_O: ({Ln_2_Te_2_W_34_}, Ln = Dy^3+^, *n* = 16, *m* = 23; Ho^3+^, *n* = 15, *m* = 24; Er^3+^, *n* = 15, *m* = 25; Tm^3+^ and Yb^3+^, *n* = 15, *m* = 28; Lu^3+^, *n* = 15, *m* = 30).

A mixture containing Na_2_WO_4_·2H_2_O (4.50 mmol, 1.50 g), Te(OH)_6_ (0.44 mmol, 0.10 g), dimethylamine hydrochloride (7.35 mmol, 0.60 g), citric acid monohydrate (0.26 mmol, 0.05 g), and distilled water (20 mL) was charged to a 25 ml glass beaker. The pH was adjusted to 4.5 by dropping 6 M HCl under stirring. After that, LnCl_3_·6H_2_O (0.33 mmol, 0.12 g) was added to the mixture, forming a uniform suspension and the pH was adjusted to 1.7. The suspension was stirred at room temperature for about 5 min and filtered. The clear filtrate was placed in a refrigerator at 10°C for 5 days and then evaporated at the ambient environment after filtering again. The cubic-shaped crystals were observed after 1 month, and at that time, the pH of the mother liquor is 2.7. Element analysis (%) for {Dy_2_Te_2_W_34_}: calcd. C 3.83, N 2.23, W 62.2, Te 2.54, Dy 3.23; found: C 3.39, N 2.06, W 61.2, Te 2.53, Dy 3.12; yield: 34% based on W. Element analysis (%) for {Ho_2_Te_2_W_34_}: calcd. C 3.59, N 2.10, W 62.3, Te 2.55, Ho 3.29; found: C 3.41, N 2.10, W 61.3, Te 2.57, Ho 3.15; yield: 30% based on W. Element analysis (%) for {Er_2_Te_2_W_34_}: calcd. C 3.59, N 2.09, W 62.2, Te 2.54, Er 3.33; found: C 3.70, N 2.29, W 60.5, Te 2.60, Er 3.10; yield: 32% based on W. Element analysis (%) for {Tm_2_Te_2_W_34_}: calcd. C 3.57, N 2.08, W 61.8, Te 2.53, Tm 3.34; found: C 3.61, N 2.24, W 62.0, Te 2.60, Tm 3.30; yield: 35% based on W. Element analysis (%) for {Yb_2_Te_2_W_34_}: calcd. C 3.56, N 2.08, W 61.8, Te 2.52, Yb 3.42; found: C 3.57, N 2.11, W 61.2, Te 2.50, Yb 3.23; yield: 36% based on W. Element analysis (%) for {Lu_2_Te_2_W_34_}: calcd. C 3.55, N 2.07, W 61.6, Te 2.51, Lu 3.45; found: C 3.61, N 2.14, W 61.3, Te 2.60, Lu 3.40; yield: 37% based on W.

Synthesis of (DMAH)_7_Na_2_{H_2_Ln(H_2_O)_4_[TeW_17_O_61_]}^·^mH_2_O: ({LnTeW_17_}, Ln = Dy^3+^, *m* = 23; Ho^3+^ and Lu^3+^, *m* = 27; Er^3+^, *m* = 22; Tm^3+^, *m* = 21; Yb^3+^, *m* = 26).

The synthetic procedure of {LnTeW_17_} was similar to that for {Ln_2_Te_2_W_34_}, and the only difference is that the pH of the reaction mixture was adjusted to 1.9. When the rod-shaped crystals were obtained, the pH of the mother liquor is 2.9. Element analysis (%) for {DyTeW_17_}: calcd. C 3.20, N 1.87, Na 0.88, W 59.6, Te 2.43, Dy 3.10; found: C 3.23, N 1.78, Na 0.96, W 60.6, Te 2.40, Dy 3.06; yield: 17% based on W. Element analysis (%) for {HoTeW_17_}: calcd. C 3.16, N 1.84, Na 0.86, W 58.7, Te 2.40, Ho 3.10; found: C 3.20, N 1.73, Na 0.86, W 57.2, Te 2.34, Ho 3.09; yield: 17% based on W. Element analysis (%) for {ErTeW_17_}: calcd. C 3.21, N 1.87, Na 0.88, W 59.7, Te 2.44, Er 3.20; found: C 3.21, N 1.73, Na 0.89, W 60.8, Te 2.50, Er 3.15; yield: 20% based on W. Element analysis (%) for {TmTeW_17_}: calcd. C 3.22, N 1.88, Na 0.88, W 59.9, Te 2.45, Tm 3.24; found: C 3.23, N 1.75, Na 0.87, W 58.9, Te 2.50, Tm 3.20; yield: 20% based on W. Element analysis (%) for {YbTeW_17_}: C 3.16, N 1.85, Na 0.87, W 58.8, Te 2.40, Yb 3.26; found: C 3.32, N 1.78, Na 0.83, W 58.2, Te 2.40, Yb 3.26; yield: 19% based on W. Element analysis (%) for {LuTeW_17_}: C 3.15, N 1.84, Na 0.86, W 58.6, Te 2.39, Lu 3.28; found: C 3.09, N 1.80, Na 0.86, W 59.5, Te 2.46, Lu 3.35; yield: 20% based on W.

Synthesis of (DMAH)_*n*_{H_3−*n*_Ln(H_2_O)_4_[TeW_6_O_24_]}^·^mH_2_O: ({LnTeW_6_}, Ln = Dy^3+^, *n* = 1, *m* = 4; Ho^3+^, *n* = 2, *m* = 1; Er^3+^, *n* = 1, *m* = 10; Tm^3+^, *n* = 2, *m* = 8; Yb^3+^, *n* = 1, *m* = 7.5; Lu^3+^, *n* = 1.5, *m* = 10).

{LnTeW_6_} was synthesized by the procedure similar to that for {Ln_2_Te_2_W_34_} except that pH = 2.3. The hexagon-shaped crystals were observed after 1 month, and at that time the pH of the mother liquor is 3.2. Element analysis (%) for {DyTeW_6_}: calcd. C 1.22, N 0.71, W 56.0, Te 6.48, Dy 8.25; found: C 1.14, N 0.68, W 55.7, Te 6.45, Dy 8.21; yield: 13% based on W. Element analysis (%) for {HoTeW_6_}: calcd. C 2.49, N 1.45, W 57.1, Te 6.61, Ho 8.54; found: C 2.56, N 1.43, W 57.5, Te 6.67, Ho 8.59; yield: 16% based on W. Element analysis (%) for {ErTeW_6_}: calcd. C 1.15, N 0.67, W 53.0, Te 6.13, Er 8.03; found: C 1.10, N 0.65, W 53.6, Te 6.18, Er 8.10; yield: 16% based on W. Element analysis (%) for {TmTeW_6_}: calcd. C 2.30, N 1.34, W 52.7, Te 6.10, Tm 8.07; found: C 2.24, N 1.25, W 52.5, Te 6.06, Tm 8.01; yield: 17% based on W. Element analysis (%) for {YbTeW_6_}: calcd. C 1.18, N 0.69, W 54.0, Te 6.25, Yb 8.47; found: C 1.14, N 0.75, W 54.2, Te 6.28, Yb 8.51; yield: 18% based on W. Element analysis (%) for {LuTeW_6_}: calcd. C 1.71, N 0.99, W 52.2, Te 6.04, Lu 8.28; found: C 1.74, N 1.03, W 51.8 Te 6.01, Lu 8.23; yield: 20% based on W.

### Catalytic Tests

Before the catalytic reaction, ground crystal samples were pretreated in a vacuum oven at 100°C for 3 h. The scanning electron microscopy (SEM) image (Supplementary Figure 9) shows that, after the pretreatment, micron-sized samples with an irregular morphology were obtained. Typically, aldehyde or ketone (1 mmol), trimethylsilycyanide (TMSCN, 2 mmol), and catalysts (2 mol%) were loaded into a 25 ml Shrek tube, which was purged three times with argon gas and heated at 45°C for 12 h. After the reaction, the mixture was diluted with 2 ml acetonitrile and added with naphthalene (0.75 mmol) as internal standard. Finally, the mixture was centrifuged and monitored quantitatively by gas chromatography. The catalysts were collected, washed with acetonitrile, and dried under vacuum for the characterization and the next run.

## Results and Discussions

### Syntheses and Structures

All crystal compounds were prepared by using Na_2_WO_4_·2H_2_O, Te(OH)_6_, dimethylamine hydrochloride, citric acid, and LnCl_3_·6H_2_O as starting materials in aqueous solution. Although citric acid does not appear in the final structures, the control experiments show that citric acid is important during the synthetic process. When we used another weak organic acid (e.g., acetic acid or amino acid) instead of citric acid, the yields of these crystal compounds dramatically decreased. According to previous investigations, we speculate that citric acid might play the role of a protective agent to coordinate with Ln^3+^ ions in preventing the formation of precipitates (Li et al., 2012; Wang et al., 2015). Moreover, it was found that dimethylamine hydrochloride is indispensable for the synthesis of crystal compounds. In the absence of dimethylamine hydrochloride, under the same reaction conditions, only numerous precipitation was obtained.

### Description of {Ln_2_Te_2_W_34_} and {LnTeW_17_} Structures

**Figure 1:**
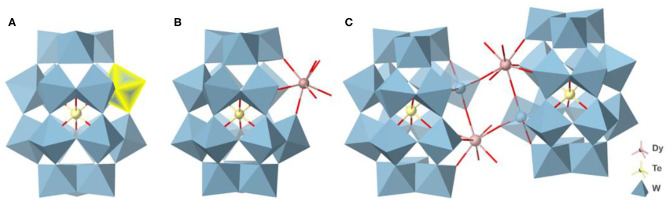
The polyhedral and ball-and-stick view of **(A)** {TeW_18_O_62_}, **(B)** {DyTeW_17_}, and **(C)** {Dy_2_Te_2_W_34_}. The cations and the lattice H_2_O molecules are omitted for clarity.

Single-crystal X-ray crystallographic analyses reveal that both {Ln_2_Te_2_W_34_} and {LnTeW_17_} crystallize in the triclinic space group of *P-1*. {LnTeW_17_} is mono-Ln^3+^-substituted Dawson-like POMs, while {Ln_2_Te_2_W_34_} has 2:2 dimeric clusters based on {LnTeW_17_} (Ln = Dy^3+^, Ho^3+^, Er^3+^, Tm^3+^, Yb^3+^, Lu^3+^). Due to the structural similarity, only the structures of {Dy_2_Te_2_W_34_} and {DyTeW_17_} are described here. {DyTeW_17_} consists of one Dy^3+^ ion, one {TeW_17_O_61_} polyanion, seven DMAH^+^ cations, two Na^+^ ions, two protons, and four coordinated and 23 lattice water molecules. Dawson-like {TeW_18_O_62_} was first reported by Cronin and Long in 2009 (Yan et al., 2009), where a {Te^VI^O_6_} octahedron is encapsulated into {W_18_O_54_} cage (Figure 1A). The {TeW_17_O_61_} can be regarded as a monovacant structure of {TeW_18_O_62_} formed by losing one [WO]^4+^ from the belt position. Compared with {TeW_18_O_62_}, the {Te^VI^O_6_} octahedron in {DyTeW_17_} still lies in the center of the cluster but exhibits a slight distortion because one [WO]^4+^ unit is replaced by a Dy^3+^ ion. The Te-O bond lengths of {DyTeW_17_} are in the range of 1.930(5)−2.010(6) Å, deviating from an ideal bond length [1.981(6) Å] in {TeW_18_O_62_}. The Dy^3+^ ion is incorporated into the vacant position of {TeW_17_O_61_} (Figure 1B) and coordinated with eight oxygen atoms: four from monovacant POM and four from coordinated water molecules. The inserted Dy^3+^ ion exhibits a distorted bicapped trigonal prismatic geometry with Dy-O bond lengths of 2.292(6)−2.464(7) Å (Supplementary Figure 1C). Due to the contraction effect of lanthanide, the Ln-O bond lengths decrease with the decrease of Ln^3+^ ionic radii (shown in Supplementary Table 2).

{Dy_2_Te_2_W_34_} is composed of two Dy^3+^ ions, two monovacant [TeW_17_O_61_]^14−^ polyanions, 16 DMAH^+^ cations, six protons, and six coordinated and 23 lattice H_2_O molecules. The dimeric {Dy_2_Te_2_W_34_} is composed of two mono-substituted {DyTeW_17_} subunits, which are connected by a belt-to-belt linking mode through Dy-O bonds (Figure 1C). In this cluster, the two Dy^3+^ ions also display distorted bicapped trigonal prismatic coordination geometries, coordinating by five terminal oxygen atoms (O4, O5, O6, O7, and O8) from two monovacant [TeW_17_O_61_]^14−^ polyanions [Dy-O: 2.305(6)−2.374(7) Å] and three H_2_O molecules [O1w, O2w and O3w) (Dy-O: 2.370(8)−2.434(9) Å] (Supplementary Figure 1E).

### Description of {LnTeW_6_} Structures

**Figure 2:**
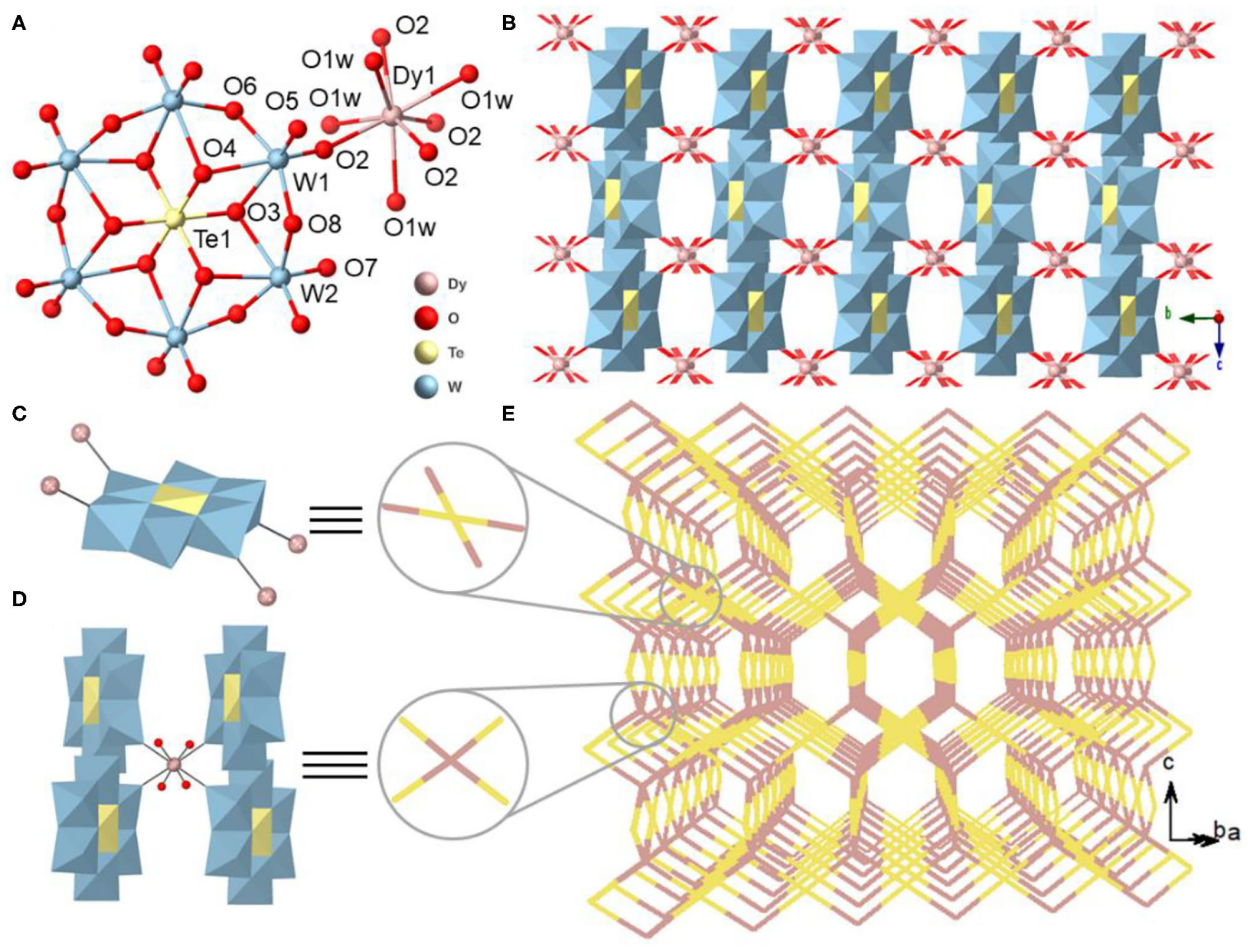
**(A)** View of the coordination of an Anderson-type [TeW_6_O_24_]^6−^ polyanion to one hydrated Dy^3+^ cation. **(B)** View of 3D inorganic structure of {DyTeW_6_} compound. Simplified view of **(C)** [TeW_6_O_24_]^6−^ polyanion and **(D)** Dy^3+^ cation as the four-connecting sites. **(E)** 3D schematic illustration of {DyTeW_6_} (yellow: [TeW_6_O_24_]^6−^ polyanion; purple: Dy^3+^ cation). The DMAH^+^ cations and the lattice H_2_O molecules are omitted for clarity.

Single-crystal X-ray crystallographic analyses reveal that the six {LnTeW_6_} compounds are isostructural and crystallize in the orthorhombic space group of *Cccm*. As a result, only the structure of {DyTeW_6_} is described in detail. {DyTeW_6_} contains one Dy^3+^ ion, one [TeW_6_O_24_]^6−^ polyanion, one DMAH^+^ cation, two protons, and four coordinated and four lattice H_2_O molecules. The [TeW_6_O_24_]^6−^ cluster shows an A-type Anderson structure where the central Te(VI) is surrounded by six edge-sharing WO_6_ octahedra (Figure 2A). The octahedral {TeO_6_} has a slight distortion with O-Te-O bond angles in the range of 85.0(2)° to 95.0(2)°.

As shown in Figure 2C, each [TeW_6_O_24_]^6−^ acts as a four-connecting node and connects with four Dy^3+^ ions through the terminal O2 atoms. The Dy^3+^ center exhibits a square antiprismatic geometry completed by four H_2_O molecules (O1w) and four terminal oxygen atoms (O2) from four adjacent [TeW_6_O_24_]^6−^ polyanions, and the Dy-O bond lengths are from 2.410(4) to 2.342(4) Å (Supplementary Figure 2A). As shown in Figure 2B and Supplementary Figure 2B, the alternating connection of [TeW_6_O_24_]^6−^ polyanions and Dy^3+^ cations by sharing O2 atoms leads to a 3D inorganic framework. From the topological views, both [TeW_6_O_24_]^6−^ and Dy^3+^ can be regarded as four-connecting sites (Figures 2C,D) and a (4,4)-connected PtS topology can be abstracted (Figure 2E).

It was found that the assembly of three types of Ln-containing tungstotellurates(VI) is pH dependent. Using {TeW_17_O_61_} as a building block, dimeric {Ln_2_Te_2_W_34_} and mono-substituted {LnTeW_17_} were isolated at pH 1.7 and 1.9, respectively. At pH 1.8, {Ln_2_Te_2_W_34_} and {LnTeW_17_} crystallized together. When the solution pH was adjusted to 2.3, 3D inorganic frameworks {LnTeW_6_} based on Anderson-type {TeW_6_O_24_} were obtained. The results indicate that the size of tungstotellurates clusters decreases with the increase of pH. A similar trend is reported in Tb-containing tungstotellurates(VI) (Shang et al., 2018). In addition, we found that the assembly of Ln-containing POMs is influenced by the type of Ln^3+^ ions. The use of Ln^3+^ ions with a relatively smaller ionic radius [e.g., Tb^3+^ (Shang et al., 2018), Dy^3+^, Ho^3+^, Er^3+^, Tm^3+^, Yb^3+^, or Lu^3+^] leads to the formation of {Ln_2_Te_2_W_34_}, {LnTeW_17_}, and {LnTeW_6_} in the pH range of 1.7–2.3. However, when starting from Ln^3+^ ions with a relatively larger ionic radius (e.g., La^3+^, Ce^3+^, Pr^3+^, Nd^3+^, Sm^3+^, or Eu^3+^), under the otherwise identical conditions only precipitate or {TeW_18_O_62_} clusters were observed. Notably, dimeric clusters, [H_10_(WO_2_){Ln(H_2_O)_5_(TeW_18_O_65_)}_2_]^12−^, were prepared at pH 1.5 by using La^3+^, Ce^3+^, Pr^3+^, Nd^3+^, Sm^3+^, Eu^3+^, or Tb^3+^, where two {TeW_18_O_65_} units are bridged by one {WO_2_} and two Ln^3+^ (Shang et al., 2018; Yang et al., 2018). Nevertheless, such structural motif cannot be obtained when using Dy^3+^, Ho^3+^, Er^3+^, Tm^3+^, Yb^3+^, or Lu^3+^ as starting materials. Therefore, it is obvious that both pH and the type of Ln^3+^ ions have a significant impact on the assembly of these Ln-containing tungstotellurates(VI) (Scheme 1).

### FT-IR Spectra

Since both {Ln_2_Te_2_W_34_} and {LnTeW_17_} are based on monovacant {TeW_17_O_61_}, their FT-IR spectra exhibit similar characteristic absorption peaks. As shown in Supplementary Figures 3, 4, the characteristic peaks at 1,020 cm^−1^ correspond to the antisymmetric stretching vibrations of Te-O bonds and the peaks at 942–948, 812–818, and 743–757 cm^−1^ are attributed to the vibrations of terminal W=O bonds, bridging W-O_b_-W (b: edge-shared O atoms) and W-O_c_-W (c: corner-shared O atoms), respectively. Compared with the plenary Dawson-like [TeW_18_O_62_]^10−^ (1,019, 949, and 799 cm^−1^) (Yan et al., 2009), some characteristic absorption peaks of POMs are slightly shifted, which might be caused by inserting Ln^3+^ ions in the vacant site of monovacant {TeW_17_O_61_}. The FT-IR spectra of {LnTeW_6_} are illustrated in Supplementary Figure 5. The characteristic peaks in the range of 1,000–400 cm^−1^ are very similar. The peaks between 970 and 950 cm^−1^ are assigned to the terminal W=O bonds, and those in the range of 920–630 cm^−1^ are the characteristic stretching vibrations of the W-O-W bridges.

Moreover, the characteristic peaks of DMAH^+^ countercations can be observed in these compounds. The peaks at 3,110–3,132 and 2,778–2,790 cm^−1^ are assigned to the stretching vibrations of N-H and C-H bonds, respectively, while the peaks at 1,558–1,640 and 1,456–1,466 cm^−1^ are attributed to the bending vibrations of N-H and C-H bonds, respectively. The broad peaks at 3,400–3,432 cm^−1^ are the stretching vibrations of H_2_O molecules. In addition, we observed that the crystals of Ln-containing tungstotellurates(VI) can easily turn to powder when leaving the mother liquor due to the loss of lattice water molecules. However, the POM skeleton is still maintained, which has been demonstrated by the FT-IR spectra.

### Catalytic Activities of {LnTeW_6_}

**Figure 3:**
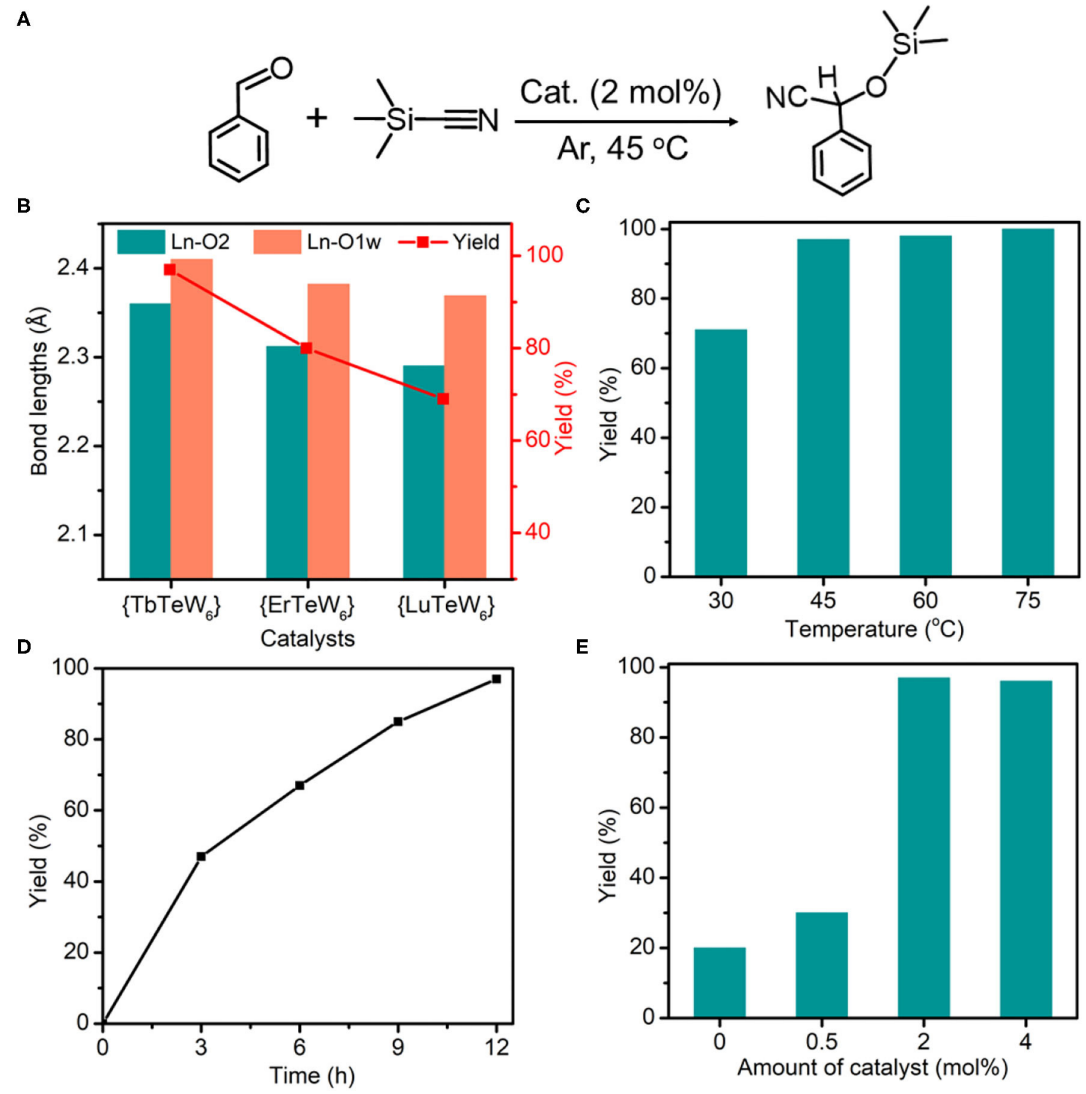
**(A)** The cyanosilylation reaction of benzaldehyde with trimethylsilylcyanide (TMSCN). **(B)** The Ln–O_2_ bond lengths and the yield of cyanosilylation catalyzed by {TbTeW_6_}, {ErTeW_6_}, and {LuTeW_6_}, respectively. **(C)** Effect of temperature on cyanosilylation using {TbTeW_6_}. **(D)** Time profile for cyanosilylation catalyzed by {TbTeW_6_}. **(E)** Effect of the amounts of {TbTeW_6_} on cyanosilylation. Reaction conditions: benzaldehyde (1 mmol), TMSCN (2 mmol), {TbTeW_6_} (2 mol%, relative to the benzaldehyde), naphthalene (0.75 mmol, internal standard) under Ar atmosphere, 45°C, 12 h.

Our previous electrospray ionization–mass spectrometry investigations show that the {Tb_2_Te_2_W_34_} and {TbTeW_17_} clusters are unstable in solution, dissociating into stable {TeW_18_O_62_} and other fragments (Shang et al., 2018). In comparison, the 3D inorganic framework {LnTeW_6_} is stable, and the Lewis acidic centers (Ln^3+^) are accessible after removing the lattice and the coordinated water molecules. The cyanosilylation reaction is an important method to prepare cyanohydrins, which can be further converted into value-added chemicals (e.g., α-hydroxy ketones, α-hydroxy acids, and β-amino alcohols) and drug molecules (Brunel and Holmes, 2004; Jia et al., 2014). Therefore, the cyanosilylation of benzaldehyde with TMSCN under solvent-free conditions was used as a reaction model to evaluate the Lewis acid catalytic activity of {LnTeW_6_} (Figure 3A). As no byproduct was obtained in the reaction, the yield of cyanohydrin trimethylsilyl ethers was calculated based on the conversion of benzaldehyde.

In our work, seven isostructural {LnTeW_6_} frameworks (Ln = Tb^3+^, Dy^3+^, Ho^3+^, Er^3+^, Tm^3+^, Yb^3+^, and Lu^3+^) were synthesized, which provides a good platform to investigate the effect of Ln ionic radius on the Lewis acid catalytic activity. As the decrease of ionic radius from Tb^3+^ (0.0923 Å) to Lu^3+^ (0.0848 Å) is unobvious, only three representative catalysts, {TbTeW_6_}, {ErTeW_6_}, and {LuTeW_6_}, were used. As shown in Figure 3B, all of {TbTeW_6_}, {ErTeW_6_}, and {LuTeW_6_} can promote the cyanosilylation reaction with a yield of 69–97%. In contrast, only 20% yield was obtained in the absence of a catalyst. Interestingly, it was found that the catalytic activity of {LnTeW_6_} decreased with the decrease of Ln ionic radius, giving the order of {TbTeW_6_} (97%) > {ErTeW_6_} (80%) > {LuTeW_6_} (69%). On the one hand, the Ln-O2 bond lengths (O2: terminal oxygen atom of {TeW_6_O_24_}) decrease from Tb^3+^ to Lu^3+^ in the order of Tb-O2 (2.360 Å) > Er-O2 (2.312 Å) > Lu-O2 (2.290 Å). The decrease of Ln-O2 bond length leads to the increase of steric hindrance around Ln^3+^ ions, and as a result, it becomes difficult for the substrates to access the Lewis acidic centers. On the other hand, the Ln-O1w bond lengths (O1w: the coordinated H_2_O molecules) also decreases from Tb^3+^ to Lu^3+^ (Tb-O1w: 2.410 Å, Er-O1w: 2.382 Å, and Lu-O1w: 2.369 Å), making the removal of coordinated H_2_O molecules difficult and resulting in the catalytic activity of Ln^3+^ ions being reduced. With the good performance of {TbTeW_6_}, it is used in the following experiments.

To explore the optimal reaction conditions, the influences of reaction temperature, time, and amount of catalyst on the cyanosilylation reaction were systematically investigated. As shown in Figure 3C, the reaction was conducted in the temperature range of 30–75°C, and a satisfactory yield (97%) was achieved at 45°C. The yield of 2-phenyl-2-[(trimethylsilyl)oxy]acetonitrile increases with the reaction time, and the maximum yield was reached after 12 h at 45°C (Figure 3D). As shown in Figure 3E, the catalyst amount of 2 mol% (relative to benzaldehyde) is an optimized dosage for this reaction.

**Figure 4:**
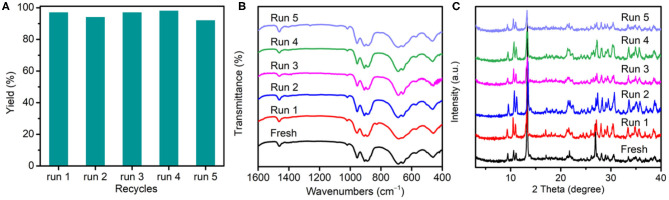
**(A)** Reusability of {TbTeW_6_} catalyst. **(B)** Fourier-transform infrared spectra and **(C)** powder X-ray diffraction patterns of the activated {TbTeW_6_} catalyst before reaction and after each cycle. Reaction conditions: benzaldehyde (1 mmol), trimethylsilylcyanide (2 mmol), {TbTeW_6_} (2 mol%, relative to the benzaldehyde), naphthalene (0.75 mmol, internal standard) under Ar atmosphere, 45°C, 12 h.

To verify the heterogeneity of {TbTeW_6_}, the catalyst was incubated in solvent at 45°C for 12 h, and the inductively coupled plasma result reveals that a negligible amount of Te, Tb, and W was detected in the filtrate. Moreover, the reusability and the stability of {TbTeW_6_} was tested under the optimized conditions. As shown in Figure 4A, {TbTeW_6_} could be reused for five times without a significant loss of its catalytic activity. The FT-IR spectra and powder X-ray diffraction (PXRD) patterns of the {TbTeW_6_} used were basically identical to those of the fresh ones (Figures 4B,C), suggesting that the structure of {TbTeW_6_} was maintained after five cycles and that Lewis acidic center Tb^3+^ is successfully stabilized by {TeW_6_O_24_} clusters. The SEM images show that the ground {TbTeW_6_} has an irregular morphology in micrometers before the reaction and after five recycles (Supplementary Figure 9). In addition, Na_6_TeW_6_O_24_ and TbCl_3_·6H_2_O were used in the reaction and gave a yield of 100%. However, Na_6_TeW_6_O_24_ is unstable under the turnover conditions, as confirmed by FT-IR and PXRD characterization (Supplementary Figure 10), and TbCl_3_·6H_2_O cannot be reused.

The effect of substituents on aromatic aldehydes was investigated, and the results are shown in Table 1. Aromatic aldehyde with electron-withdrawing group (chloro) is beneficial to the cyanosilylation reaction, giving a yield of up to 100% (2 in Table 1). However, a significant decrease of yield was observed by using aromatic aldehydes with electron-donating groups (methoxy) (3–5 in Table 1). Among them, the yield of **5** (29%) with two methoxy substituents is much lower than those of **3** (49%) and **4** (66%) with one methoxy substituent. Generally, ketones are less reactive than aldehydes in cyanosilylation reaction, and thus only 8% yield was obtained using acetophenone as substrate (6 in Table 1).

**Table 1:** The cyanosilylation reaction catalyzed by {TbTeW_6_}*^a^*.

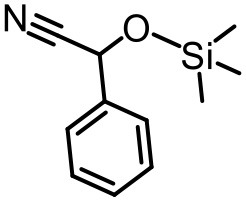	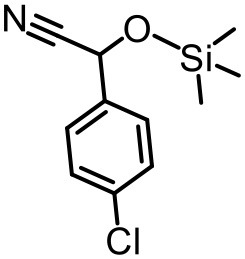	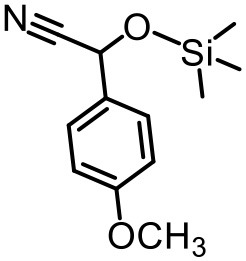
**1** (97%)	**2** (100%)	**3** (49%)
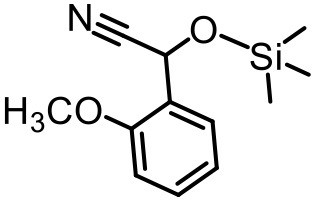	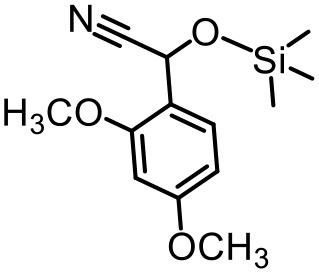	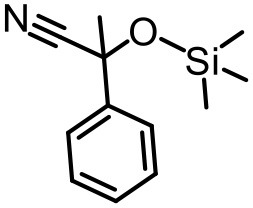
**4** (66%)	**5** (29%)	**6** (8%)

a *Reaction conditions: substrate (1 mmol), trimethylsilylcyanide (2 mmol), {TbTeW_6_} (2 mol%, relative to the benzaldehyde), naphthalene (0.75 mmol, internal standard) under Ar atmosphere, 45°C, 12 h. The yield of the corresponding cyanohydrin trimethylsilyl ethers was monitored by gas chromatography with flame ionization detector*.

## Conclusions

In summary, a series of Ln-containing tungstotellurates(VI) have been isolated and structurally characterized. The assembly of dimeric {Ln_2_Te_2_W_34_}, mono-substituted {LnTeW_17_}, and 3D inorganic framework {LnTeW_6_} is pH dependent, which were formed at pH 1.7, 1.9, and 2.3, respectively. Importantly, the type of Ln^3+^ ions plays an important role in the assembly process. The three types of Ln-containing POMs can be synthesized by using Tb^3+^-Lu^3+^ ions with a relatively smaller radius, while when starting from La^3+^-Eu^3+^ ions only precipitation or {TeW_18_O_62_} clusters were observed. Moreover, three {LnTeW_6_} (Ln = Tb^3+^, Er^3+^, Lu^3+^) are selected as heterogeneous Lewis acid catalysts for the cyanosilylation reaction. It was found that the catalytic activity of {LnTeW_6_} decreases with the decrease of Ln^3+^ ionic radius. The {TbTeW_6_} catalyst exhibits excellent stability and can be reused for five times without a significant loss of activity. The successful isolation of Ln-containing POMs not only contributes to the understanding of Ln-containing POM assembly but also provides a good platform to investigate the influence of Ln ionic radius on their Lewis acid catalytic activity.

## Data Availability Statement

The datasets presented in this study can be found in online repositories. The names of the repository/repositories and accession number(s) can be found in the article/Supplementary Material.

## Author Contributions

YC and CH supervised the project. JL, SS, and NZ prepared the catalysts. ZLin and ZY analyzed the crystallographic data. JL performed the catalytic experiments and wrote the manuscript. ZLi participated in the analysis of the results. All authors contributed to the article and approved the submitted version.

## Conflict of Interest

SS was employed by company China Resources Double-Crane Pharmaceutical Co., Ltd. The remaining authors declare that the research was conducted in the absence of any commercial or financial relationships that could be construed as a potential conflict of interest.

## References

[B1] Arab Fashapoyeh M. Mirzaei M. Eshtiagh-Hosseini H. Rajagopal A. Lechner M. Liu R. (2018). Photochemical and electrochemical hydrogen evolution reactivity of lanthanide-functionalized polyoxotungstates. Chem. Commun. 54, 10427–10430. 10.1039/C8CC06334F 30159565

[B2] Boglio C. Lemière G. Hasenknopf B. Thorimbert S. Lacôte E. Malacria M. (2006). Lanthanide complexes of the monovacant dawson polyoxotungstate [α_1_-P_2_W_17_O_61_]^10−^ as selective and recoverable lewis acid catalysts. Angew. Chem. Int. Ed. 45, 3324–3327. 10.1002/anie.200600364 16619320

[B3] Brunel J. M. Holmes I. P. (2004). Chemically catalyzed asymmetric cyanohydrin syntheses. Angew. Chem. Int. Ed. 43, 2752–2778. 10.1002/anie.200300604 15150747

[B4] Chen W. C. Jiao C. Q. Wang X. L. Shao K. Z. Su Z. M. (2019). Self-assembly of nanoscale lanthanoid-containing selenotungstates: synthesis, structures, and magnetic studies. Inorg. Chem. 58, 12895–12904. 10.1021/acs.inorgchem.9b01830 31532221

[B5] Chen W. C. Li H. L. Wang X. L. Shao K. Z. Su Z. M. Wang E. B. (2013). Assembly of cerium(III)-stabilized polyoxotungstate nanoclusters with ${\text{SeO}}_{3}^{2-}$/${\text{TeO}}_{3}^{2-}$ templates: from single polyoxoanions to inorganic hollow spheres in dilute solution. Chem. Eur. J. 19, 11007–11015. 10.1002/chem.201300615 23794258

[B6] Chen W. C. Qin C. Wang X. L. Li Y. G. Zang H. Y. Shao K. Z.. (2015). Assembly of a large cerium(III)-containing tungstotellurites(IV) nanocluster: [Ce_10_Te_8_W_88_O_298_(OH)_12_(H_2_O)_40_]^18−^. Dalton Trans. 44, 11290–11293. 10.1039/C5DT01711D 26053387

[B7] Chen W. C. Wang X. L. Jiao Y. Q. Huang P. Zhou E. L. Su Z. M.. (2014). pH-controlled and sulfite anion-directed assembly of a family of cerium(III)-containing polyoxotungstates clusters. Inorg. Chem. 53, 9486–9497. 10.1021/ic500442k 25153270

[B8] Chen Y. Sun L. Chang S. Chen L. Zhao J. (2018). Synergistic effect between different coordination geometries of lanthanides and various coordination modes of 2-picolinic acid ligands tuning three types of rare 3d-4f heterometallic tungstoantimonates. Inorg. Chem. 57, 15079–15092. 10.1021/acs.inorgchem.8b02103 30480437

[B9] Clemente-Juan J. M. Coronado E. Gaita-Ariño A. (2012). Magnetic polyoxometalates: from molecular magnetism to molecular spintronics and quantum computing. Chem. Soc. Rev. 41, 7464–7478. 10.1039/c2cs35205b 22948854

[B10] Cronin L. Müller A. (2012). From serendipity to design of polyoxometalates at the nanoscale, aesthetic beauty and applications. Chem. Soc. Rev. 41, 7333–7334. 10.1039/c2cs90087d 23052289

[B11] Dolomanov O. V. Bourhis L. J. Gildea R. J. Howard J. A. K. Puschmann H. (2009). OLEX2: a complete structure solution, refinement and analysis program. J. Appl. Cryst. 42, 339–341. 10.1107/S0021889808042726

[B12] Gao J. Yan J. Mitchell S. G. Miras H. N. Boulay A. G. Long D. L. (2011). Self-assembly of a family of macrocyclic polyoxotungstates with emergent material properties. Chem. Sci. 2, 1502–1508. 10.1039/c1sc00150g

[B13] Granadeiro C. M. Ferreira R. A. S. Soares-Santos P. C. R. Carlos L. D. Trindade T. Nogueira H. I. S. (2010). Lanthanopolyoxotungstates in silica nanoparticles: multi-wavelength photoluminescent core/shell materials. J. Mater. Chem. 20, 3313–3318. 10.1039/b919691a

[B14] Han Q. Liu J. C. Wen Y. Chen L. J. Zhao J. W. Yang G. Y. (2017a). Tellurotungstate-based organotin–rare-earth heterometallic hybrids with four organic components. Inorg. Chem. 56, 7257–7269. 10.1021/acs.inorgchem.7b00924 28581730

[B15] Han Q. Wen Y. Liu J. C. Zhang W. Chen L. J. Zhao J. W. (2017b). Rare-earth-incorporated tellurotungstate hybrids functionalized by 2-picolinic acid ligands: syntheses, structures, and properties. Inorg. Chem. 56, 13228–13240. 10.1021/acs.inorgchem.7b02009 29048174

[B16] Hill C. L. (1998). Introduction: polyoxometalatesmulticomponent molecular vehicles to probe fundamental issues and practical problems. Chem. Rev. 98, 1–2. 10.1021/cr960395y 11851497

[B17] Ismail A. H. Nsouli N. H. Dickman M. H. Knez J. Kortz U. (2009). The 20-Tungsto-4-tellurate(IV) [H_2_Te_4_W_20_O_80_]^22−^ and the 15-tungstotellurate(IV) [NaTeW_15_O_54_]^13−^. J. Cluster Sci. 20, 453–465. 10.1007/s10876-009-0245-6

[B18] Jia Y. Zhao S. Song Y. F. (2014). The application of spontaneous flocculation for the preparation of lanthanide-containing polyoxometalates intercalated layered double hydroxides: highly efficient heterogeneous catalysts for cyanosilylation. Appl. Catal. A Gen. 487, 172–180. 10.1016/j.apcata.2014.09.005

[B19] Kaushik R. Khan I. Saini M. K. Hussain F. Sadakane M. (2018). Synthesis and characterization of carbonate-encapsulated ytterbium- and yttrium-containing polyoxotungstates. Acta Crystallogr. Sect. C 74, 1355–1361. 10.1107/S2053229618011841 30398188

[B20] Li F. Guo W. Xu L. Ma L. Wang Y. (2012). Two dysprosium-incorporated tungstoarsenates: synthesis, structures and magnetic properties. Dalton Trans. 41, 9220–9226. 10.1039/c2dt12277d 22729234

[B21] Li S. Zhou Y. Peng Q. Wang R. Feng X. Liu S.. (2018). Controllable synthesis and catalytic performance of nanocrystals of rare-earth-polyoxometalates. Inorg. Chem. 57, 6624–6631. 10.1021/acs.inorgchem.8b00763 29749730

[B22] Liu J. C. Han Q. Chen L. J. Zhao J. W. Streb C. Song Y. F. (2018). Aggregation of giant cerium-bismuth tungstate clusters into a 3d porous framework with high proton conductivity. Angew. Chem. Int. Ed. 57, 8416–8420. 10.1002/anie.201803649 29683244

[B23] Liu J. C. Zhao J. W. Song Y. F. (2019). 1-D chain tungstotellurate hybrids constructed from organic-ligand-connecting iron-lanthanide heterometal encapsulated tetrameric polyoxotungstate units. Inorg. Chem. 58, 9706–9712. 10.1021/acs.inorgchem.9b00618 31318540

[B24] Liu J. L. Jin M. T. Chen L. J. Zhao J. W. (2018). First dimethyltin-functionalized rare-earth incorporated tellurotungstates consisting of {B-α-TeW_7_O_28_} and {W_5_O_18_} mixed building units. Inorg. Chem. 57, 12509–12520. 10.1021/acs.inorgchem.8b01486 30281291

[B25] Ma X. Yang W. Chen L. Zhao J. (2015). Significant developments in rare-earth-containing polyoxometalate chemistry: synthetic strategies, structural diversities and correlative properties. CrystEngComm 17, 8175–8197. 10.1039/C5CE01240F

[B26] Mialane P. Lisnard L. Mallard A. Marrot J. Antic-Fidancev E. Aschehoug P.. (2003). Solid-State and solution studies of {Ln_n_(SiW_11_O_39_)} polyoxoanions: an example of building block condensation dependent on the nature of the rare earth. Inorg. Chem. 42, 2102–2108. 10.1021/ic020486f 12639147

[B27] Mougharbel A. S. Bhattacharya S. Bassil B. S. Rubab A. van Leusen J. Kogerler P.. (2020). Lanthanide-containing 22-tungsto-2-germanates [Ln(GeW_11_O_39_)_2_]^13−^: synthesis, structure, and magnetic properties. Inorg. Chem. 59, 4340–4348. 10.1021/acs.inorgchem.9b03271 32133839

[B28] Ozeki T. Yamase T. (1994). Effect of lanthanide contraction on the structures of the decatungstolanthanoate anions in K_3_Na_4_H_2_[LnW_10_O_36_].nH_2_0 (Ln = Pr, Nd, Sm, Gd, Tb, Dy) crystals. Acta Crystallogr. Sect. B 50:128 10.1107/S0108768193011553

[B29] Ritchie C. Alley K. G. Boskovic C. (2010a). Lacunary tungstotellurates(iv): [Te_2_W_17_O_61_]^12−^, [Te_2_W_16_O_58_(OH)_2_]^14−^ and [Te_2_W_18_O_62_(OH)_2_]^10−^. Dalton Trans. 39, 8872–8874. 10.1039/c0dt00547a 20820614

[B30] Ritchie C. Moore E. G. Speldrich M. Kögerler P. Boskovic C. (2010b). Terbium polyoxometalate organic complexes: correlation of structure with luminescence properties. Angew. Chem. Int. Ed. 49, 7702–7705. 10.1002/anie.201002320 20836101

[B31] Schmidt K. J. Schrobilgen G. J. Sawyer J. F. (1986). Hexasodium hexatungstotellurate(VI) 22-hydrate. Acta Crystallogr. Sect. C 42, 1115–1118. 10.1107/S0108270186093204

[B32] Shah H. S. Al-Oweini R. Haider A. Kortz U. Iqbal J. (2014). Cytotoxicity and enzyme inhibition studies of polyoxometalates and their chitosan nanoassemblies. Toxicol. Rep. 1, 341–352. 10.1016/j.toxrep.2014.06.001 28962250 PMC5598103

[B33] Shang S. Lin Z. Yin A. Yang S. Chi Y. Wang Y.. (2018). Self-assembly of Ln(III)-containing tungstotellurates(VI): correlation of structure and photoluminescence. Inorg. Chem. 57, 8831–8840. 10.1021/acs.inorgchem.8b00693 30015477

[B34] Sheldrick G. M. (2015). Crystal structure refinement with SHELXL. Acta Crystallogr. Sect. C 71, 3–8. 10.1107/S2053229614024218 25567568 PMC4294323

[B35] Suzuki K. Sato R. Mizuno N. (2013). Reversible switching of single-molecule magnet behaviors by transformation of dinuclear dysprosium cores in polyoxometalates. Chem. Sci. 4, 596–600. 10.1039/C2SC21619A

[B36] Suzuki K. Tang F. Kikukawa Y. Yamaguchi K. Mizuno N. (2014). Visible-light-induced photoredox catalysis with a tetracerium-containing silicotungstate. Angew. Chem. Int. Ed. 53, 5356–5360. 10.1002/anie.201403215 24740892

[B37] Wang Y. Sun X. Li S. Ma P. Niu J. Wang J. (2015). Generation of large polynuclear rare earth metal-containing organic–inorganic polytungstoarsenate aggregates. Cryst. Growth Des. 15, 2057–2063. 10.1021/cg5012499

[B38] Yan J. Long D. L. Wilson E. F. Cronin L. (2009). Discovery of heteroatom-“embedded” Te {W_18_O_54_} nanofunctional polyoxometalates by use of cryospray mass spectrometry. Angew. Chem. Int. Ed. 48, 4376–4380. 10.1002/anie.200806343 19434635

[B39] Yang G. P. Shang S. X. Yu B. Hu C. W. (2018). Ce(iii)-Containing tungstotellurate(vi) with a sandwich structure: an efficient Lewis acid–base catalyst for the condensation cyclization of 1,3-diketones with hydrazines/hydrazides or diamines. Inorg. Chem. Front. 5, 2472–2477. 10.1039/C8QI00678D

[B40] Zhang D. Zhang C. Chen H. Ma P. Wang J. Niu J. (2012). Syntheses, structures and properties of dimeric rare earth derivatives based on monovacant keggin-type polyoxotungstates. Inorg. Chim. Acta 391, 218–223. 10.1016/j.ica.2012.04.030

[B41] Zhang Y. Wang D. Zeng B. Chen L. Zhao J. Yang G. Y. (2020). An unprecedented polyhydroxycarboxylic acid ligand bridged multi-Eu^III^ incorporated tellurotungstate and its luminescence properties. Dalton Trans. 49, 8933–8948. 10.1039/D0DT00729C 32558836

[B42] Zhao H. Y. Yang B. F. Yang G. Y. (2017). Two new 2D organic–inorganic hybrids assembled by lanthanide-substituted polyoxotungstate dimers and copper–complex linkers. Inorg. Chem. Commun. 84, 212–216. 10.1016/j.inoche.2017.08.027

[B43] Zhao J. W. Li H. L. Ma X. Xie Z. Chen L. J. Zhu Y. (2016a). Lanthanide-connecting and lone-electron-pair active trigonal-pyramidal-AsO_3_ inducing nanosized poly(polyoxotungstate) aggregates and their anticancer activities. Sci. Rep. 6:26406. 10.1038/srep26406 27193961 PMC4872259

[B44] Zhao J. W. Li Y. Z. Chen L. J. Yang G. Y. (2016b). Research progress on polyoxometalate-based transition-metal-rare-earth heterometallic derived materials: synthetic strategies, structural overview and functional applications. Chem. Commun. 52, 4418–4445. 10.1039/C5CC10447E 26894638

[B45] Zhao J. W. Li Y. Z. Ji F. Yuan J. Chen L. J. Yang G. Y. (2014). Syntheses, structures and electrochemical properties of a class of 1-D double chain polyoxotungstate hybrids [H_2_dap][Cu(dap)_2_]_0.5_[Cu(dap)_2_(H_2_O)][Ln(H_2_O)_3_(alpha-GeW_11_O_39_)]·3H_2_O. Dalton Trans. 43, 5694–5706. 10.1039/C3DT53616E 24554042

