# Supplementary material for: Assembly of Lanthanide-Containing Tungstotellurates(VI): Syntheses, Structures, and Catalytic Properties
Source: Front Chem. 2020 Nov 23;8:598961. doi: 10.3389/fchem.2020.598961 (PMC7719746; doi:10.3389/fchem.2020.598961)
Supplement: Supplementary file 1 [file Table_1.DOCX]

Supplementary Materials

Index

Supplementary **Figure 1**. The ball-and-stick views of {TeW_18_O_62_}, {DyTeW_17_} and {Dy_2_Te_2_W_34_}. 2

Supplementary **Figure 2**. The coordination geometry of Dy ion and the view of 3D inorganic structure in {DyTeW_6_}. 2

Supplementary **Figure 3**. FT-IR spectra of {Ln_2_Te_2_W_34_}. 3

Supplementary **Figure 4**. FT-IR spectra of {LnTeW_17_}. 3

Supplementary **Figure 5**. FT-IR spectra of {LnTeW_6_}. 4

Supplementary **Figure 6**. TGA curves of {Ln_2_Te_2_W_34_}. 4

Supplementary **Figure 7**. TGA curves of {LnTeW_17_}. 5

Supplementary **Figure 8**. TGA curves of {LnTeW_6_}. 5

Supplementary **Figure 9**. SEM images of {TbTeW_6_} catalyst before reaction and after five cycles….6

Supplementary **Figure 10**. FT-IR spectra and PXRD patterns of Na_6_TeW_6_O_24_ catalyst before reaction and after three cycles. 6

Supplementary **Table S1**. Crystallographic data and structure refinements for {Ln_2_Te_2_W_34_}, {LnTeW_17_} and {LnTeW_6_}. 7

Supplementary **Table S2**. The selected bond lengths and angles for {Ln_2_Te_2_W_34_}, {LnTeW_17_} and {LnTeW_6_}. 10

**
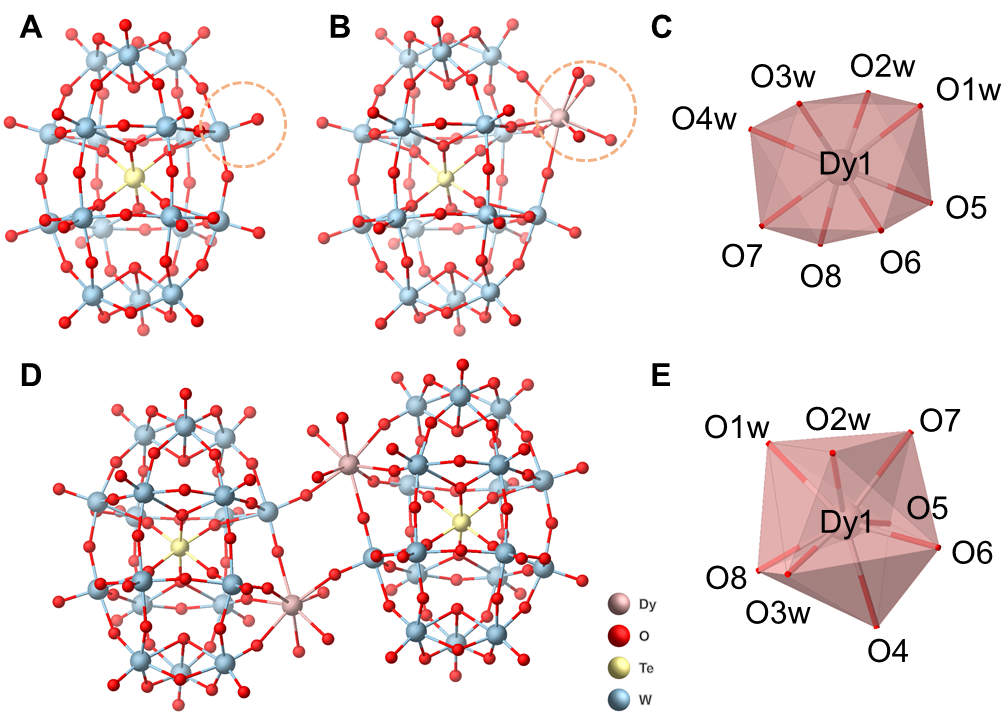
**

# Supplementary Figure 1. The ball-and-stick views of (A) {TeW_18_O_62_}, (B) {DyTeW_17_}, and (D) {Dy_2_Te_2_W_34_}. The coordination geometry of Dy ion in {DyTeW_17_} (C) and in {Dy_2_Te_2_W_34_} (E).

#
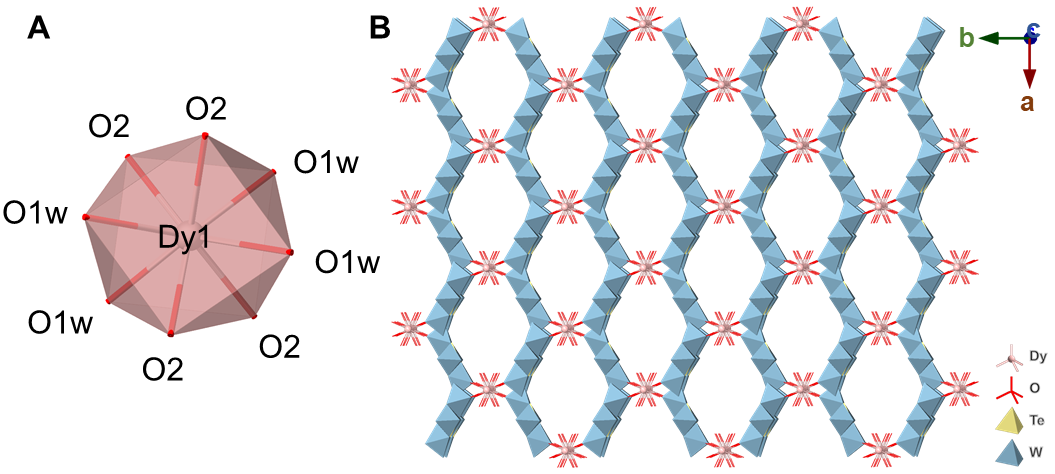


# Supplementary Figure 2. (A) The coordination geometry of Dy ion in {DyTeW_6_}. (B) View of 3D inorganic structure in {DyTeW_6_}.


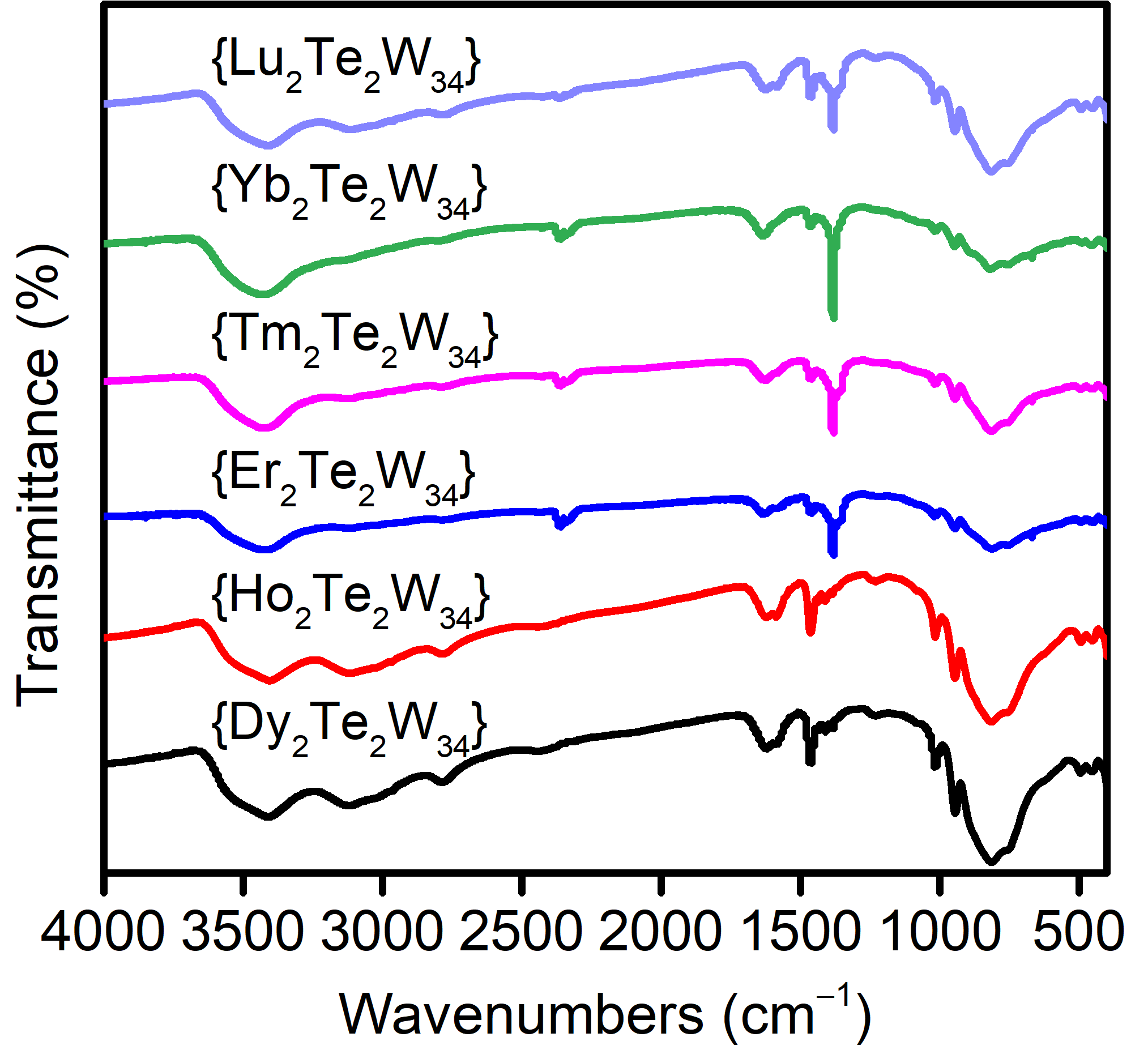


# Supplementary Figure 3. FT-IR spectra of {Ln_2_Te_2_W_34_}.


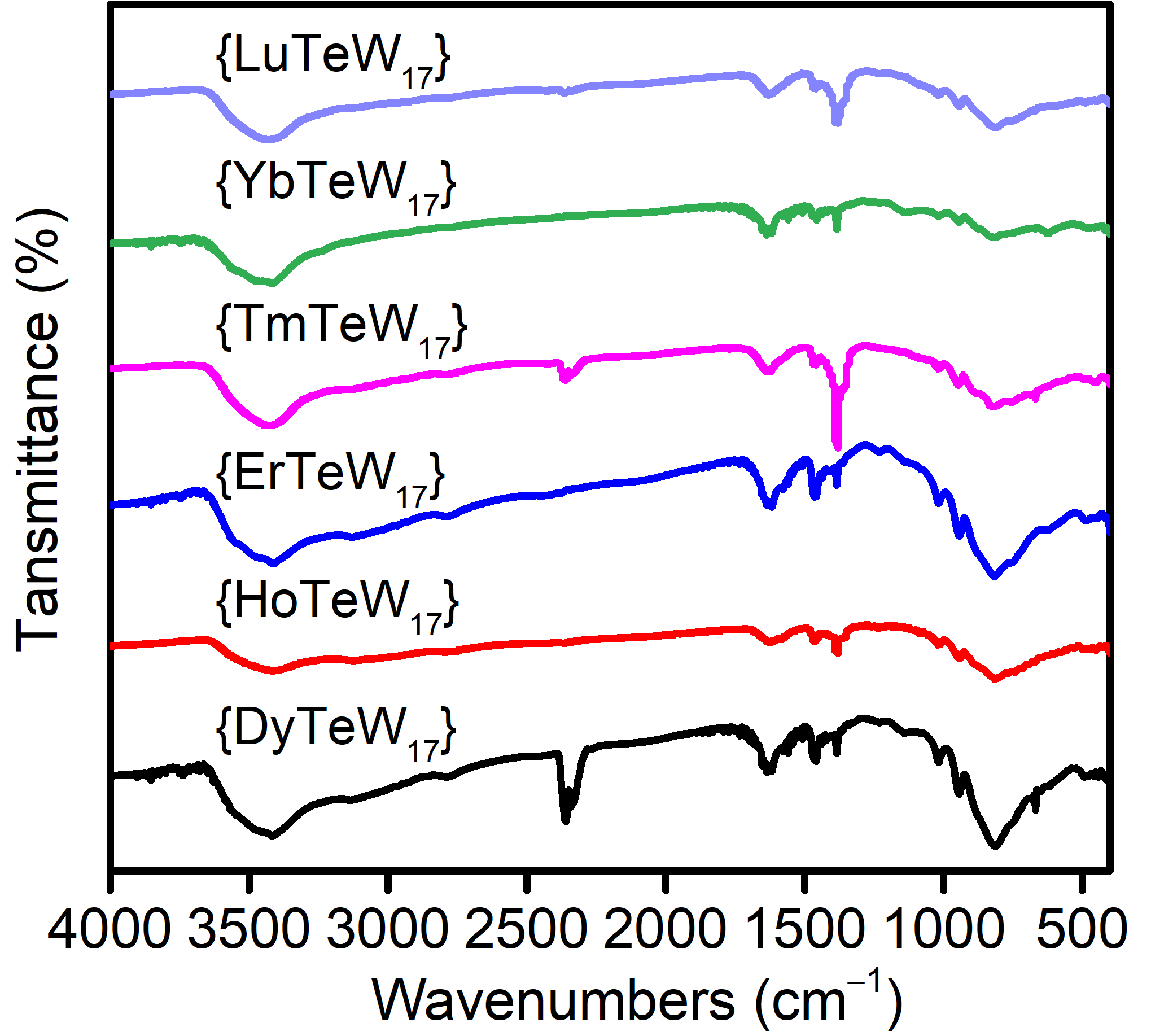


# Supplementary Figure 4. FT-IR spectra of {LnTeW_17_}.

**
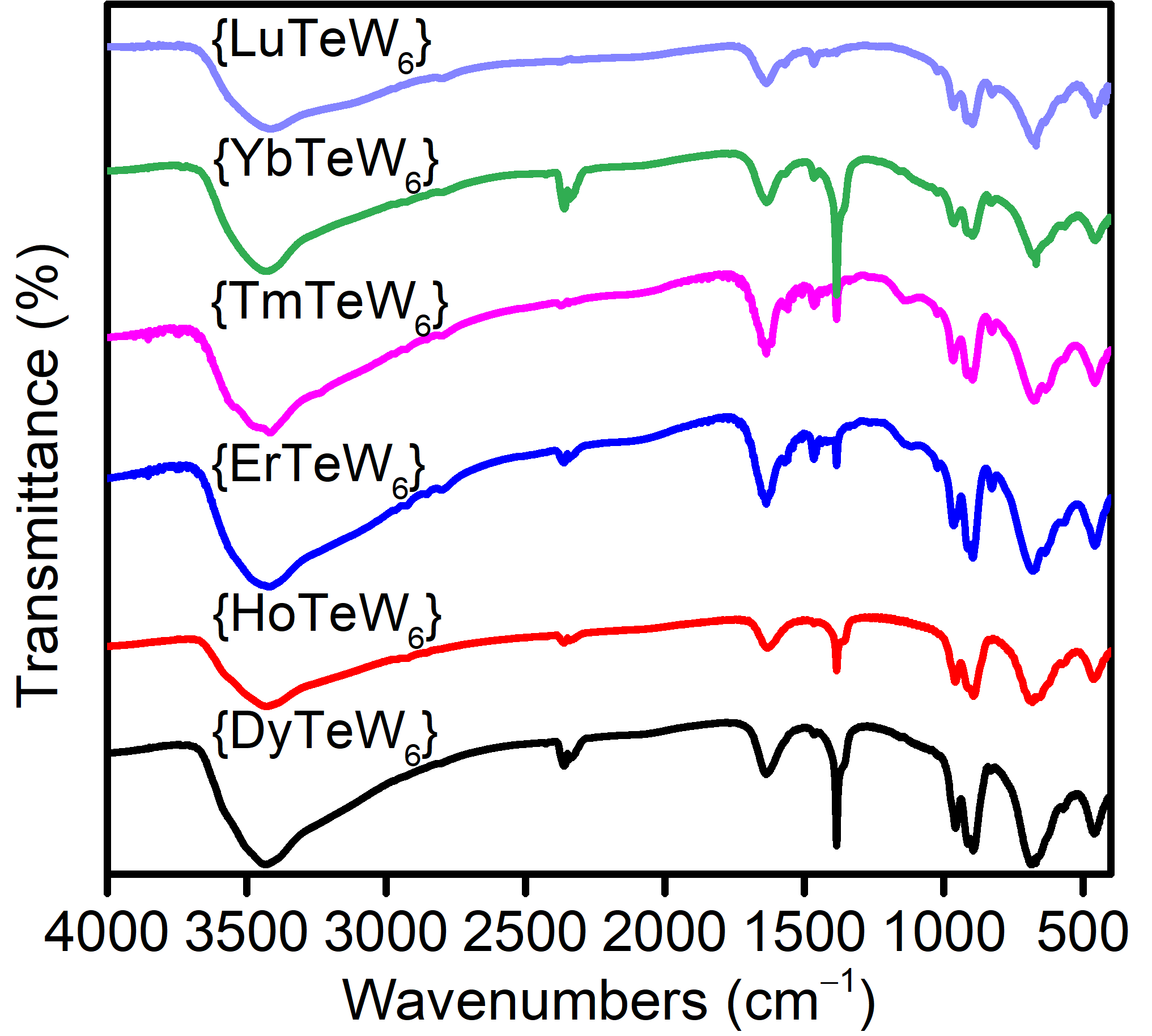
**

# Supplementary Figure 5. FT-IR spectra of {LnTeW_6_}.


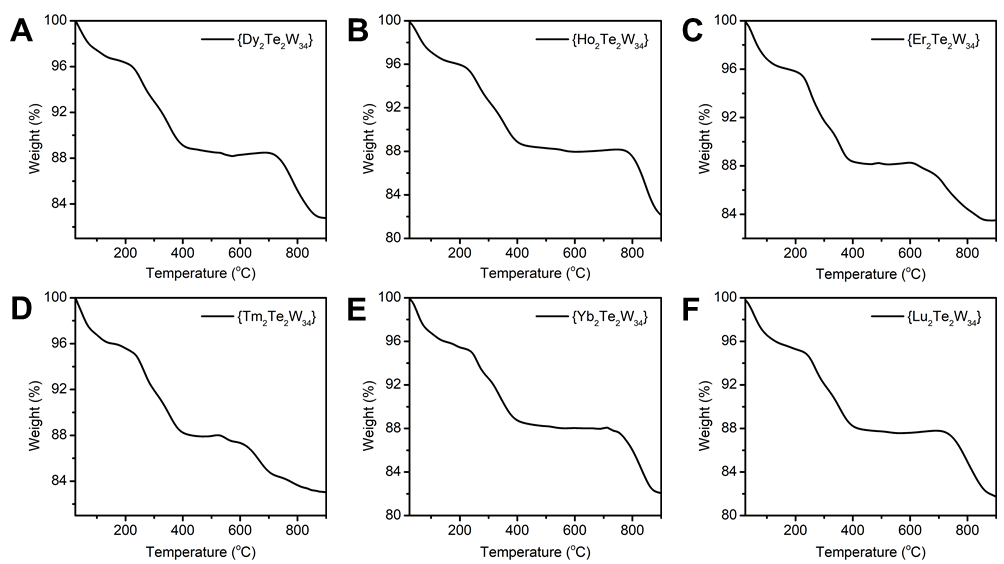


# Supplementary Figure 6. TGA curves of {Ln_2_Te_2_W_34_}. The TG analyses of {Ln_2_Te_2_W_34_} were carried out under a N_2_ atmosphere in the temperature range of 25-900 ^o^C. {Ln_2_Te_2_W_34_} exhibit four weight losses. The first weight losses between 25 and 220 ^o^C with 3.43% for {Dy_2_Te_2_W_34_}, 4.03% for {Ho_2_Te_2_W_34_}, 4.06% for {Er_2_Te_2_W_34_}, 4.86% for {Tm_2_Te_2_W_34_}, 4.68% for {Yb_2_Te_2_W_34_} and 4.87% for {Lu_2_Te_2_W_34_}, are ascribed to the loss of lattice H_2_O molecules. The following two continuous weight losses below 600 ^o^C correspond to the losses of four coordinated H_2_O molecules and DMAH^+^ countercations (weight loss: 8.39%, 8.00%, 7.82%, 7.63%, 7.58% and 7.57%). Finally, {Ln_2_Te_2_W_34_} skeletons start to collapse from 600 ^o^C.


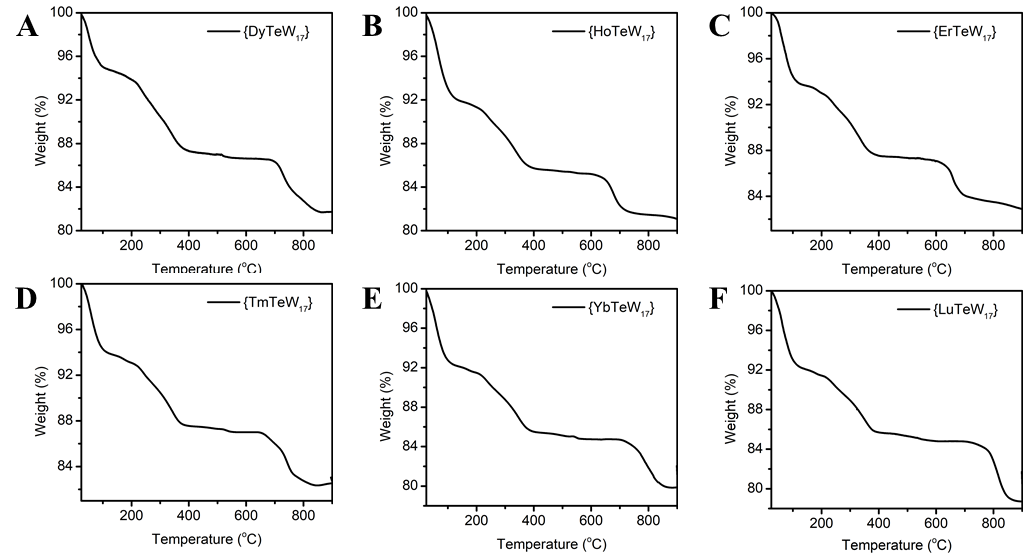


# Supplementary Figure 7. TGA curves of {LnTeW_17_}. The TG analyses of {LnTeW_17_} exhibit four weight losses. The first weight losses between 25 and 220 ^o^C with the loss of 4.96% for {GdTeW_17_}, 5.94% for {DyTeW_17_}, 7.92% for {HoTeW_17_}, 7.84% for {ErTeW_17_}, 6.83% for {TmTeW_17_}, 8.34% for {YbTeW_17_} and 8.81% for {LuTeW_17_}, correspond to the loss of lattice H_2_O molecules. The following two continuous weight losses below 600 ^o^C ascribe to the losses of four coordinated H_2_O molecules and DMAH^+^ countercations (weight loss: 7.30%, 7.45%, 7.05%, 7.84%, 7.18% 7.27% and 7.30%). Finally, {LnTeW_17_} skeletons start to collapse from 650 ^o^C.


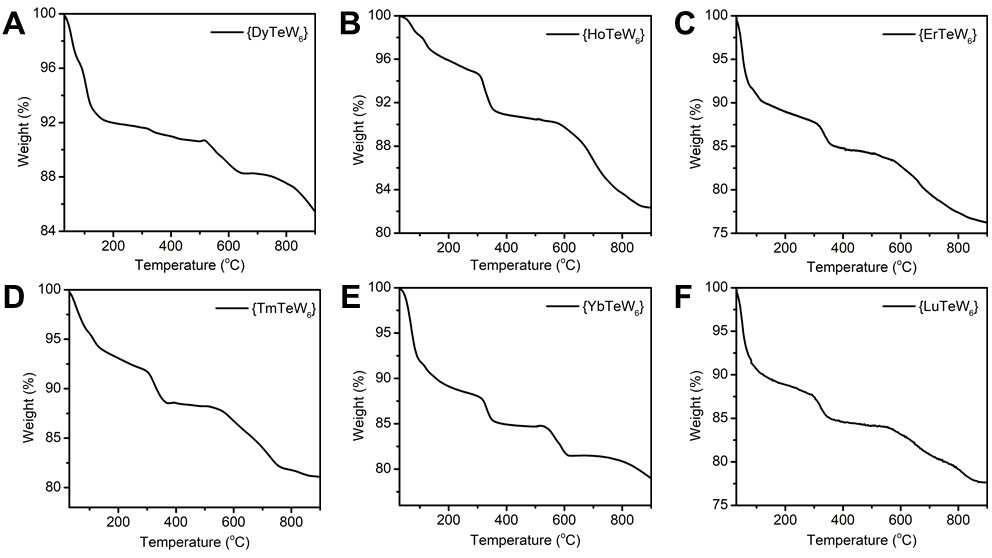


# Supplementary Figure 8. TGA curves of {LnTeW_6_}. The TG curves of {LnTeW_6_} exhibit four weight losses. The first three consecutive weight losses from 30 to 550 ^o^C with the loss of 9.38% for {DyTeW_6_}, 9.51% for {HoTeW_6_}, 14.85% for {ErTeW_6_}, 12.07% for {TmTeW_6_}, 13.20% for {YbTeW_6_} and 15.42% for {LuTeW_6_}, correspond to the loss of lattice H_2_O molecules, four coordinated H_2_O molecules and DMAH^+^ countercations, respectively. Then, the whole inorganic frameworks begins to collapse from 550 ^o^C.


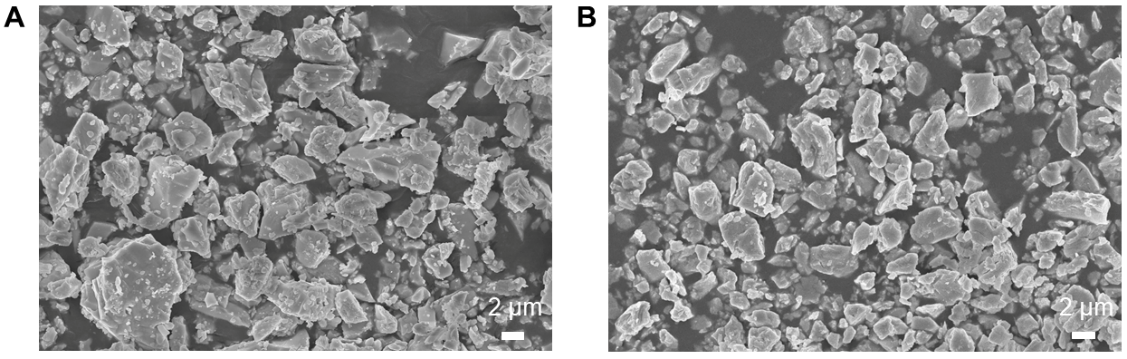


# Supplementary Figure 9. SEM images of {TbTeW_6_} catalyst (A) before reaction and (B) after five cycles.


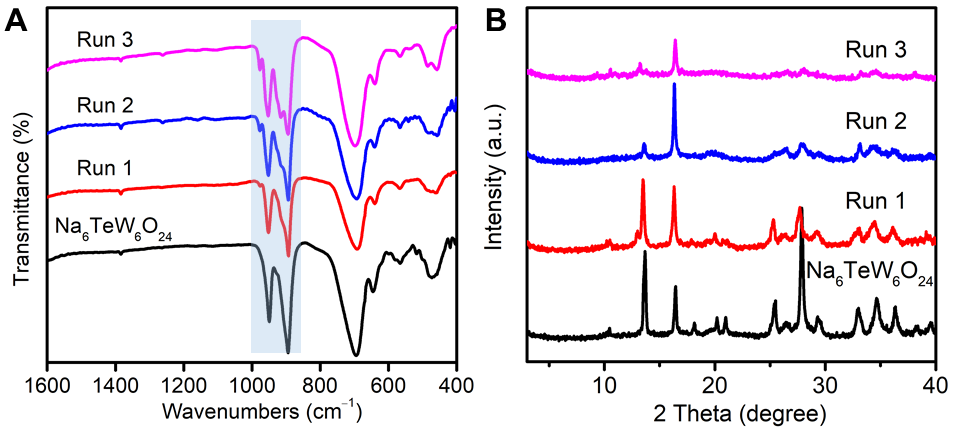


# Supplementary Figure 10. (A) FT-IR spectra and (B) PXRD patterns of Na_6_TeW_6_O_24_ catalyst before reaction and after three cycles. Reaction conditions: Benzaldehyde (1 mmol), TMSCN (2 mmol), Na_6_TeW_6_O_24_ (2 mol%, relative to the benzaldehyde), naphthalene (0.75 mmol) as internal standard under Ar atmosphere, 45 ^o^C, 12 h.

# Supplementary Table S1. Crystallographic data and structure refinements for {Ln_2_Te_2_W_34_}, {LnTeW_17_} and {LnTeW_6_}.

|  | {Dy_2_Te_2_W_34_} | {Ho_2_Te_2_W_34_} | {Er_2_Te_2_W_34_} | {Tm_2_Te_2_W_34_} | {Yb_2_Te_2_W_34_} | {Lu_2_Te_2_W_34_} |
| --- | --- | --- | --- | --- | --- | --- |
| Formula | C_32_H_192_N_16_Te_2_  Dy_2_W_34_O_151_ | C_30_H_187_N_15_Te_2_  Ho_2_W_34_O_152_ | C_30_H_189_N_15_Te_2_  Er_2_W_34_O_153_ | C_30_H_195_N_15_Te_2_  Tm_2_W_34_O_156_ | C_30_H_195_N_15_Te_2_  Yb_2_W_34_O_156_ | C_30_H_199_N_15_Te_2_  Lu_2_W_34_O_158_ |
| *M*r | 10048.64 | 10026.44 | 10049.11 | 10106.50 | 10114.7 | 10154.6 |
| Temperature (K) | 296(2) | 296(2) | 296(2) | 296(2) | 296(2) | 296(2) |
| Crystal system | Triclinic | Triclinic | Triclinic | Triclinic | Triclinic | Triclinic |
| Space group | *P*-*1* | *P*-*1* | *P*-*1* | *P*-*1* | *P*-*1* | *P*-*1* |
| *a* (Å) | 14.0216(3) | 14.0253(3) | 14.0161(3) | 14.1499(5) | 14.0328(3) | 14.0315(3) |
| *b* (Å) | 14.4472(2) | 14.4358(3) | 14.4504(2) | 14.5884(5) | 14.4419(3) | 14.4627(3) |
| *c* (Å) | 23.5468(4) | 23.5321(4) | 23.5104(3) | 23.7181(7) | 23.4762(4) | 23.4500(4) |
| *α* (deg) | 106.1480(10) | 106.1507(18) | 106.1960(10) | 106.195(3) | 106.2190(10) | 106.240(2) |
| *β* (deg) | 94.7260(10) | 94.6938(17) | 94.6860(10) | 94.581(3) | 94.5790(10) | 94.616(2) |
| *γ* (deg) | 110.5840(2) | 110.605(2) | 110.566(2) | 110.596(3) | 110.586(2) | 110.580(2) |
| *V* (Å^3^) | 4202.26(14) | 4197.21(17) | 4194.98(13) | 4313.7(3) | 4191.90(15) | 4191.86(16) |
| *Z* | 1 | 1 | 1 | 1 | 1 | 1 |
| *D*_calcd_ (g cm^−3^) | 3.660 | 3.658 | 3.664 | 3.540 | 3.665 | 3.679 |
| *μ* (mm^−1^) | 24.467 | 24.548 | 24.619 | 23.994 | 24.751 | 24.814 |
| *F* (000) | 3968.0 | 3959.0 | 3964.0 | 3934.0 | 3959.0 | 3978.0 |
| Reflections collected/unique | 53399/24685 | 55756/24778 | 50942/24370 | 38813/16039 | 51678/24399 | 54605/24701 |
| GOF on *F*^2^ | 1.066 | 1.053 | 1.064 | 1.070 | 1.072 | 1.026 |
| final *R* indexes (*I* > 2*σ*(*I*)) | *R*_1_ = 0.0477 ^a^  *wR*_2_ = 0.1165 ^b^ | *R*_1_ = 0.0494 ^a^  *wR*_2_ = 0.1033 ^b^ | *R*_1_ = 0.0503 ^a^  *wR*_2_ = 0.1314 ^b^ | *R*_1_ = 0.0463 ^a^  *wR*_2_ = 0.1167 ^b^ | *R*_1_ = 0.0533 ^a^  *wR*_2_ = 0.1326 ^b^ | *R*_1_ = 0.0440 ^a^  *wR*_2_ = 0.0960 ^b^ |
| *R* indexes (all data) | *R*_1_ = 0.0650 ^a^  *wR*_2_ = 0.1222 ^b^ | *R*_1_ = 0.0767 ^a^  *wR*_2_ = 0.1106 ^b^ | *R*_1_ = 0.0633 ^a^  *wR*_2_ = 0.1374 ^b^ | *R*_1_ = 0.0607 ^a^  *wR*_2_ = 0.1226 ^b^ | *R*_1_ = 0.0717 ^a^  *wR*_2_ = 0.1400 ^b^ | *R*_1_ = 0.0630 ^a^  *wR*_2_ = 0.1013 ^b^ |

|  | {DyTeW_17_} | {HoTeW_17_} | {ErTeW_17_} | {TmTeW_17_} | {YbTeW_17_} | {LuTeW_17_} |
| --- | --- | --- | --- | --- | --- | --- |
| Formula | C_14_H_112_N_7_Na_2_  TeDyW_17_O_88_ | C_14_H_120_N_7_Na_2_  TeHoW_17_O_92_ | C_14_H_110_N_7_Na_2_  TeErW_17_O_87_ | C_14_H_108_N_7_Na_2_  TeTmW_17_O_86_ | C_14_H_118_N_7_Na_2_  TeYbW_17_O_91_ | C_14_H_120_N_7_Na_2_  TeLuW_17_O_92_ |
| *M*r | 5248.39 | 5322.88 | 5235.14 | 5218.80 | 5312.98 | 5332.92 |
| Temperature (K) | 296(2) | 296(2) | 296(2) | 296(2) | 296(2) | 296(2) |
| Crystal system | Triclinic | Triclinic | Triclinic | Triclinic | Triclinic | Triclinic |
| Space group | *P*-*1* | *P*-*1* | *P*-*1* | *P*-*1* | *P*-*1* | *P*-*1* |
| *a* (Å) | 13.1380(2) | 13.1394(2) | 13.1527(3) | 13.1395(4) | 13.1532(3) | 13.1283(3) |
| *b* (Å) | 14.8767(3) | 14.8740(3) | 14.9285(4) | 14.8770(4) | 14.8728(3) | 14.8448(4) |
| *c* (Å) | 24.9758(4) | 24.9631(4) | 24.9988(6) | 24.9813(5) | 24.9642(4) | 24.9474(6) |
| *α* (deg) | 102.256(2) | 102.246(2) | 102.295(2) | 102.428(2) | 102.3920(10) | 102.527(2) |
| *β* (deg) | 96.2060(10) | 96.183(2) | 96.046(2) | 96.280(2) | 96.258(2) | 96.464(2) |
| *γ* (deg) | 90.5280(10) | 90.589(2) | 90.808(2) | 90.385(2) | 90.499(2) | 90.290(2) |
| *V* (Å^3^) | 4739.71(15) | 4737.17(15) | 4765.9(2) | 4737.9(2) | 4738.83(17) | 4713.8(2) |
| *Z* | 2 | 2 | 2 | 2 | 2 | 2 |
| *D*_calcd_ (g cm^−3^) | 3.149 | 3.207 | 3.181 | 3.198 | 3.206 | 3.186 |
| *μ* (mm^−1^) | 21.686 | 21.749 | 21.667 | 21.845 | 21.892 | 22.061 |
| *F* (000) | 3830.0 | 3910.0 | 3900.0 | 3898.0 | 3908.0 | 3856.0 |
| Reflections collected/unique | 63454/27973 | 61587/27751 | 60261/27774 | 60485/27838 | 60777/27699 | 61771/27781 |
| GOF on *F*^2^ | 1.036 | 1.083 | 1.062 | 1.100 | 1.093 | 1.045 |
| final *R* indexes (*I* > 2*σ*(*I*)) | *R*_1_ = 0.0428 ^a^  *wR*_2_ = 0.0945 ^b^ | *R*_1_ = 0.0474 ^a^  *wR*_2_ = 0.1095 ^b^ | *R*_1_ = 0.0641 ^a^  *wR*_2_ = 0.1528 ^b^ | *R*_1_ = 0.0452 ^a^  *wR*_2_ = 0.1111 ^b^ | *R*_1_ = 0.0448 ^a^  *wR*_2_ = 0.1042 ^b^ | *R*_1_ = 0.0557 ^a^  *wR*_2_ = 0.1219 ^b^ |
| *R* indexes (all data) | *R*_1_ = 0.0602 ^a^  *wR*_2_ = 0.0987 ^b^ | *R*_1_ = 0.0668 ^a^  *wR*_2_ = 0.1152 ^b^ | *R*_1_ = 0.0882 ^a^  *wR*_2_ = 0.1624 ^b^ | *R*_1_ = 0.0598 ^a^  *wR*_2_ = 0.1160 ^b^ | *R*_1_ = 0.0605 ^a^  *wR*_2_ = 0.1085 ^b^ | *R*_1_ = 0.0803 ^a^  *wR*_2_ = 0.1294 ^b^ |

|  | {DyTeW_6_} | {HoTeW_6_} | | {ErTeW_6_} | {TmTeW_6_} | {YbTeW_6_} | {LuTeW_6_} | |
| --- | --- | --- | --- | --- | --- | --- | --- | --- |
| Formula | C_2_H_26_NTeDyW_6_O_32_ | C_4_H_27_N_2_TeHoW_6_O_27_ | | C_2_H_38_NTeErW_6_O_38_ | C_4_H_41_N_2_TeTmW_6_O_36_ | C_2_H_33_NTeYbW_6_O_35.5_ | C_3_H_41.5_N_1.5_TeLuW_6_O_38_ | |
| *M*r | 1969.35 | 1930.82 | | 2082.19 | 2092.92 | 2042.95 | 2112.44 | |
| Temperature (K) | 296(2) | 296(2) | | 296(2) | 296(2) | 296(2) | 296(2) | |
| Crystal system | Orthorhombic | Orthorhombic | | Orthorhombic | Orthorhombic | Orthorhombic | Orthorhombic | |
| Space group | *Cccm* | *Cccm* | | *Cccm* | *Cccm* | *Cccm* | *Cccm* | |
| *a* (Å) | 13.6902(4) | 13.6839(4) | | 13.6976(6) | 13.7303(5) | 13.5540(8) | 13.5556(11) | |
| *b* (Å) | 17.6372(7) | 17.5681(5) | | 17.4564(6) | 17.5197(7) | 17.4268(8) | 17.4711(13) | |
| *c* (Å) | 15.6832(6) | 15.6754(4) | | 15.6222(6) | 15.6614(6) | 15.5745(9) | 15.5599(12) | |
| *α* (deg) | 90.00 | 90.00 | | 90.00 | 90.00 | 90.00 | 90.00 | |
| *β* (deg) | 90.00 | 90.00 | | 90.00 | 90.00 | 90.00 | 90.00 | |
| *γ* (deg) | 90.00 | 90.00 | | 90.00 | 90.00 | 90.00 | 90.00 | |
| *V* (Å^3^) | 3786.8(2) | 3768.37(18) | | 3735.4(3) | 3767.4(2) | 3678.7(3) | 3685.1(5) | |
| *Z* | 4 | 4 | | 4 | 4 | 4 | 4 | |
| *D*_calcd_ (g cm^−3^) | 3.324 | 3.345 | | 3.297 | 3.258 | 3.412 | 3.341 | |
| *μ* (mm^−1^) | 20.930 | 21.149 | | 21.458 | 21.402 | 22.052 | 22.151 | |
| *F* (000) | 3252.0 | 3256.0 | | 3168.0 | 3156.0 | 3236.0 | 3164.0 | |
| Reflections collected/unique | 24677/3124 | 23895/3017 | | 23875/3066 | 23520/3035 | 23721/3012 | 20734/2388 | |
| GOF on *F*^2^ | 1.053 | 1.141 | | 1.087 | 1.103 | 1.084 | 1.258 | |
| final *R* indexes (*I* > 2*σ*(*I*)) | *R*_1_ = 0.0276 ^a^  *wR*_2_ = 0.0635 ^b^ | *R*_1_ = 0.0436 ^a^  *wR*_2_ = 0.1111 ^b^ | | *R*_1_ = 0.0267 ^a^  *wR*_2_ = 0.0700 ^b^ | *R*_1_ = 0.0295 ^a^  *wR*_2_ = 0.0741 ^b^ | *R*_1_ = 0.0670 ^a^  *wR*_2_ = 0.1640 ^b^ | *R*_1_ = 0.0479 ^a^  *wR*_2_ = 0.1084 ^b^ | |
| *R* indexes (all data) | *R*_1_ = 0.0317 ^a^  *wR*_2_ = 0.0647 ^b^ | *R*_1_ = 0.0462 ^a^  *wR*_2_ = 0.1123 ^b^ | | *R*_1_ = 0.0282 ^a^  *wR*_2_ = 0.0706 ^b^ | *R*_1_ = 0.0336 ^a^  *wR*_2_ = 0.0789 ^b^ | *R*_1_ = 0.0726 ^a^  *wR*_2_ = 0.1671 ^b^ | *R*_1_ = 0.0480 ^a^  *wR*_2_ = 0.1085 ^b^ | |
| *^a^ R*_1_ = *∑*(\|\|*F*_o_\|-\|*F*_c_\|\|)/*∑*\|*F*_o_\|; | | | *^b^ wR*_2_ = [*∑w*(*F*_o_^2^-*F*_c_^2^)^2^/*∑w*(*F*_o_^2^)^2^]^1/2^. | | | | |  |

# Supplementary Table S2. The selected bond lengths and angles for {Ln_2_Te_2_W_34_}, {LnTeW_17_} and {LnTeW_6_}.

| Bond | Lengths (Å) | | | Bond | | | Lengths (Å) | Bond | Lengths (Å) | | | Bond | Lengths (Å) | |  |  |
| --- | --- | --- | --- | --- | --- | --- | --- | --- | --- | --- | --- | --- | --- | --- | --- | --- |
| **{Dy_2_Te_2_W_34_}** | | | | | | | | | | | | | | | | |
| Dy1-O1 | 2.373(9) | | | Dy1-O2 | | | 2.434(9) | Dy1-O3 | 2.370(8) | | | Dy1-O4 | 2.331(7) | |  |  |
| Dy1-O5 | 2.355(8) | | | Dy1-O6 | | | 2.305(6) | Dy1-O7 | 2.334(7) | | | Dy1-O8#1 | 2.374(7) | |  |  |
| Te1-O9 | 1.928(6) | | | Te1-O10 | | | 1.980(6) | Te1-O11 | 1.939(8) | | | Te1-O12 | 1.944(7) | |  |  |
| Te1-O13 | 1.952(6) | | | Te1-O14 | | | 2.012(6) |  |  | | |  |  | |  |  |
| **{Ho_2_Te_2_W_34_}** | | | | | | | | | | | | | | | | |
| Ho1-O1 | 2.367(9) | | | Ho1-O2 | | | 2.415(10) | Ho1-O3 | 2.368(8) | | | Ho1-O4 | 2.323(8) | |  |  |
| Ho1-O5 | 2.351(8) | | | Ho1-O6 | | | 2.299(7) | Ho1-O7 | 2.328(7) | | | Ho1-O8#1 | 2.360(8) | |  |  |
| Te1-O9 | 1.967(7) | | | Te1-O10 | | | 1.918(7) | Te1-O11 | 1.919(7) | | | Te1-O12 | 2.022(7) | |  |  |
| Te1-O13 | 1.945(7) | | | Te1-O14 | | | 1.955(8) |  |  | | |  |  | |  |  |
| **{Er_2_Te_2_W_34_}** | | | | | | | | | | | | | | | | |
| Er1-O1 | 2.353(9) | | | Er1-O2 | | | 2.430(9) | Er1-O3 | 2.348(9) | | | Er1-O4 | 2.315(8) | |  |  |
| Er1-O5 | 2.338(8) | | | Er1-O6 | | | 2.272(8) | Er1-O7 | 2.300(8) | | | Er1-O8#1 | 2.350(8) | |  |  |
| Te1-O9 | 1.931(8) | | | Te1-O10 | | | 1.921(7) | Te1-O11 | 1.963(7) | | | Te1-O12 | 1.940(7) | |  |  |
| Te1-O13 | 1.940(7) | | | Te1-O14 | | | 2.018(7) |  |  | | |  |  | |  |  |
| **{Tm_2_Te_2_W_34_}** | | | | | | | | | | | | | | | | |
| Tm1-O1 | 2.368(11) | | | Tm1-O2 | | | 2.445(13) | Tm1-O3 | 2.352(10) | | | Tm1-O4 | 2.348(9) | |  |  |
| Tm1-O5 | 2.356(10) | | | Tm1-O6 | | | 2.278(9) | Tm1-O7 | 2.348(10) | | | Tm1-O8#1 | 2.361(10) | |  |  |
| Te1-O9 | 2.000(9) | | | Te1-O10 | | | 2.021(8) | Te1-O11 | 1.975(9) | | | Te1-O12 | 1.990(9) | |  |  |
| Te1-O13 | 1.925(8) | | | Te1-O14 | | | 1.974(9) |  |  | | |  |  | |  |  |
| **{Yb_2_Te_2_W_34_}** | | | | | | | | | | | | | | | | |
| Yb1-O1 | 2.330(10) | | | Yb1-O2 | | | 2.408(13) | Yb1-O3 | 2.315(10) | | | Yb1-O4 | 2.296(8) | |  |  |
| Yb1-O5 | 2.313(9) | | | Yb1-O6 | | | 2.263(8) | Yb1-O7 | 2.300(9) | | | Yb1-O8#1 | 2.325(9) | |  |  |
| Te1-O9 | 1.914(8) | | | Te1-O10 | | | 1.938(9) | Te1-O11 | 1.977(7) | | | Te1-O12 | 2.011(8) | |  |  |
| Te1-O13 | 1.947(9) | | | Te1-O14 | | | 1.954(7) |  |  | | |  |  | |  |  |
| **{Lu_2_Te_2_W_34_}** | | | | | | | | | | | | | | | | |
| Lu1-O1 | 2.312(7) | | | Lu1-O2 | | | 2.302(7) | Lu1-O3 | 2.407(8) | | | Lu1-O4 | 2.307(6) | |  |  |
| Lu1-O5 | 2.243(6) | | | Lu1-O6 | | | 2.315(7) | Lu1-O7 | 2.294(6) | | | Lu1-O8#1 | 2.320(6) | |  |  |
| Te1-O9 | 1.920(6) | | | Te1-O10 | | | 1.926(7) | Te1-O11 | 1.972(6) | | | Te1-O12 | 1.945(5) | |  |  |
| Te1-O13 | 1.947(7) | | | Te1-O14 | | | 2.004(6) |  |  | | |  |  | |  |  |
| **{DyTeW_17_}** | | | | | | | | | | | | | | | | |
| Dy1-O1 | 2.346(7) | | | Dy1-O2 | | | 2.464(7) | Dy1-O3 | 2.464(7) | | | Dy1-O4 | 2.361(6) | |  |  |
| Dy1-O5 | 2.313(6) | | | Dy1-O6 | | | 2.292(6) | Dy1-O7 | 2.331(6) | | | Dy1-O8 | 2.315(6) | |  |  |
| Te1-O9 | 1.945(5) | | | Te1-O10 | | | 1.930(5) | Te1-O11 | 1.944(5) | | | Te1-O12 | 1.954(5) | |  |  |
| Te1-O13 | 1.962(5) | | | Te1-O14 | | | 2.010(6) |  |  | | |  |  | |  |  |
| **{HoTeW_17_}** | | | | | | | | | | | | | | | | |
| Ho1-O1 | 2.435(8) | | | Ho1-O2 | | | 2.334(9) | Ho1-O3 | 2.341(8) | | | Ho1-O4 | 2.447(9) | |  |  |
| Ho1-O5 | 2.303(7) | | | Ho1-O6 | | | 2.332(7) | Ho1-O7 | 2.304(7) | | | Ho1-O8 | 2.339(7) | |  |  |
| Te1-O9 | 1.949(6) | | | Te1-O10 | | | 1.953(6) | Te1-O11 | 1.971(7) | | | Te1-O12 | 1.958(7) | |  |  |
| Te1-O13 | 1.961(6) | | | Te1-O14 | | | 2.008(7) |  |  | | |  |  | |  |  |
| **{ErTeW_17_}** | | | | | | | | | | | | | | | | |
| Er1-O1 | 2.329(14) | | | Er1-O2 | | | 2.433(13) | Er1-O3 | 2.430(12) | | | Er1-O4 | 2.347(11) | |  |  |
| Er1-O5 | 2.322(10) | | | Er1-O6 | | | 2.274(11) | Er1-O7 | 2.328(12) | | | Er1-O8 | 2.330(10) | |  |  |
| Te1-O9 | 1.968(10) | | | Te1-O10 | | | 1.988(10) | Te1-O11 | 2.024(10) | | | Te1-O12 | 1.966(10) | |  |  |
| Te1-O13 | 1.971(10) | | | Te1-O14 | | | 1.954(9) |  |  | | |  |  | |  |  |
| **{TmTeW_17_}** | | | | | | | | | | | | | | | | |
| Tm1-O1 | 2.448(8) | | | Tm1-O2 | | | 2.297(8) | Tm1-O3 | 2.334(7) | | | Tm1-O4 | 2.427(8) | |  |  |
| Tm1-O5 | 2.286(6) | | | Tm1-O6 | | | 2.306(6) | Tm1-O7 | 2.275(7) | | | Tm1-O8 | 2.310(7) | |  |  |
| Te1-O9 | 1.935(6) | | | Te1-O10 | | | 1.947(6) | Te1-O11 | 1.957(6) | | | Te1-O12 | 2.009(6) | |  |  |
| Te1-O13 | 1.957(6) | | | Te1-O14 | | | 1.953(6) |  |  | | |  |  | |  |  |
| **{YbTeW_17_}** | | | | | | | | | | | | | | | | |
| Yb1-O1 | 2.305(7) | | | Yb1-O2 | | | 2.423(7) | Yb1-O3 | 2.436(8) | | | Yb1-O4 | 2.325(6) | |  |  |
| Yb1-O5 | 2.284(6) | | | Yb1-O6 | | | 2.281(6) | Yb1-O7 | 2.301(6) | | | Yb1-O8 | 2.299(6) | |  |  |
| Te1-O9 | 1.934(6) | | | Te1-O10 | | | 1.943(5) | Te1-O11 | 1.970(6) | | | Te1-O12 | 1.963(6) | |  |  |
| Te1-O13 | 1.953(5) | | | Te1-O14 | | | 2.015(6) |  |  | | |  |  | |  |  |
| **{LuTeW_17_}** | | | | | | | | | | | | | | | | |
| Lu1-O1 | 2.441(10) | | | Lu1-O2 | | | 2.297(9) | Lu1-O3 | 2.308(8) | | | Lu1-O4 | 2.414(10) | |  |  |
| Lu1-O5 | 2.271(8) | | | Lu1-O6 | | | 2.263(8) | Lu1-O7 | 2.266(8) | | | Lu1-O8 | 2.306(8) | |  |  |
| Te1-O9 | 1.933(7) | | | Te1-O10 | | | 1.944(7) | Te1-O11 | 1.973(8) | | | Te1-O12 | 1.947(7) | |  |  |
| Te1-O13 | 2.008(8) | | | Te1-O14 | | | 1.964(8) |  |  | | |  |  | |  |  |
| **{DyTeW_6_}** | | | | | | | | | | | | | | | | |
| Dy1-O1 | 2.410(4) | | | Dy1-O2 | | | 2.342(4) | Te1-O3 | 1.924(3) | | | Te1-O4 | 1.938(5) | |  |  |
| **{HoTeW_6_}** | | | | | | | | | | | | | | | | |
| Ho1-O1 | 2.400(6) | | | Ho1-O2 | | | 2.328(6) | Te1-O3 | 1.925(6) | | | Te1-O4 | 1.942(8) | |  |  |
| **{ErTeW_6_}** | | | | | | | | | | | | | | | | |
| Er1-O1 | 2.382(4) | | | Er1-O2 | | | 2.312(4) | Te1-O3 | 1.935(5) | | | Te1-O4 | 1.924(3) | |  |  |
| **{TmTeW_6_}** | | | | | | | | | | | | | | | | |
| Tm1-O1 | 2.377(5) | | | Tm1-O2 | | | 2.319(4) | Te1-O3 | 1.944(4) | | | Te1-O4 | 1.942(6) | |  |  |
| **{YbTeW_6_}** | | | | | | | | | | | | | | | | |
| Yb1-O1 | 2.361(10) | | | Yb1-O2 | | | 2.296(11) | Te1-O3 | 1.926(10) | | | Te1-O5 | 1.951(16) | |  |  |
| **{LuTeW_6_}** | | | | | | | | | | | | | | | | |
| Lu1-O1 | 2.369(9) | | | Lu1-O2 | | | 2.290(9) | Te1-O3 | 1.942(11) | | | Te1-O4 | 1.915(9) | |  |  |
| Bond | | Angle (°) | | | Bond | | | Angle (°) | | Bond | | | | Angle (°) | |  |
| **{Dy_2_Te_2_W_34_}** | | | | | | | | | | | | | | | | |
| O1-Dy1-O2 | | | 69.1(4) | | | O1-Dy1-O3 | | 101.2(4) | | | O1-Dy1-O6 | | | 88.3(3) | |  |
| O1-Dy1-O8#1 | | | 76.2(3) | | | O3-Dy1-O2 | | 76.3(4) | | | O4-Dy1-O1 | | | 138.4(3) | |  |
| O4-Dy1-O2 | | | 71.0(3) | | | O4-Dy1-O3 | | 80.1(3) | | | O4-Dy1-O6 | | | 74.2(3) | |  |
| O4-Dy1-O7 | | | 124.6(3) | | | O4-Dy1-O8#1 | | 139.1(3) | | | O5-Dy1-O1 | | | 148.3(3) | |  |
| O5-Dy1-O2 | | | 142.6(3) | | | O5-Dy1-O3 | | 91.6(3) | | | O5-Dy1-O4 | | | 72.1(3) | |  |
| O5-Dy1-O6 | | | 94.9(3) | | | O5-Dy1-O7 | | 74.1(3) | | | O5-Dy1-O8#1 | | | 81.3(3) | |  |
| O6-Dy1-O2 | | | 80.9(3) | | | O6-Dy1-O3 | | 150.0(3) | | | O7-Dy1-O1 | | | 78.5(3) | |  |
| O7-Dy1-O2 | | | 134.2(3) | | | O7-Dy1-O3 | | 143.2(3) | | | O7-Dy1-O6 | | | 66.4(3) | |  |
| O7-Dy1-O8#1 | | | 74.4(3) | | | O8#1-Dy1-O2 | | 124.9(3) | | | O8#1-Dy1-O3 | | | 70.0(3) | |  |
| O8#1-Dy1-O6 | | | 140.0(3) | | | O9-Te1-O10 | | 91.7(3) | | | O9-Te1-O11 | | | 94.6(3) | |  |
| O9-Te1-O12 | | | 91.3(3) | | | O9-Te1-O13 | | 94.5(3) | | | O9-Te1-O14 | | | 179.3(3) | |  |
| O10-Te1-O13 | | | 173.7(3) | | | O11-Te1-O10 | | 91.0(3) | | | O11-Te1-O12 | | | 174.1(3) | |  |
| O11-Te1-O13 | | | 88.7(3) | | | O11-Te1-O14 | | 85.3(3) | | | O12-Te1-O10 | | | 88.3(3) | |  |
| O12-Te1-O13 | | | 91.4(3) | | | O14-Te1-O10 | | 88.9(3) | | | O14-Te1-O12 | | | 88.9(3) | |  |
| O14-Te1-O13 | | | 84.8(3) | | |  | |  | | |  | | |  | |  |
| **{Ho_2_Te_2_W_34_}** | | | | | | | | | | | | | | | | |
| O1-Ho1-O3 | | | 101.5(3) | | | O2-Ho1-O1 | | 68.0(4) | | | O2-Ho1-O3 | | | 76.0(4) | |  |
| O2-Ho1-O8#1 | | | 123.6(3) | | | O4-Ho1-O1 | | 138.3(3) | | | O4-Ho1-O2 | | | 72.3(3) | |  |
| O4-Ho1-O3 | | | 80.5(3) | | | O4-Ho1-O5 | | 71.6(3) | | | O4-Ho1-O6 | | | 74.3(3) | |  |
| O4-Ho1-O7 | | | 123.8(3) | | | O4-Ho1-O8#1 | | 139.2(3) | | | O5-Ho1-O1 | | | 148.8(3) | |  |
| O5-Ho1-O2 | | | 143.1(3) | | | O5-Ho1-O3 | | 90.9(3) | | | O5-Ho1-O6 | | | 95.7(3) | |  |
| O5-Ho1-O8#1 | | | 81.3(3) | | | O6-Ho1-O1 | | 87.6(3) | | | O6-Ho1-O2 | | | 81.7(3) | |  |
| O6-Ho1-O3 | | | 150.4(3) | | | O6-Ho1-O8#1 | | 139.7(3) | | | O7-Ho1-O1 | | | 79.0(3) | |  |
| O7-Ho1-O2 | | | 134.6(3) | | | O7-Ho1-O3 | | 143.0(3) | | | O7-Ho1-O5 | | | 74.2(3) | |  |
| O7-Ho1-O6 | | | 66.2(3) | | | O7-Ho1-O8#1 | | 74.5(3) | | | O8#1-Ho1-O1 | | | 76.4(3) | |  |
| O8#1-Ho1-O3 | | | 69.8(3) | | | O9-Te1-O10 | | 88.9(3) | | | O9-Te1-O11 | | | 92.0(3) | |  |
| O9-Te1-O14 | | | 90.2(3) | | | O11-Te1-O10 | | 91.8(3) | | | O12-Te1-O9 | | | 88.9(3) | |  |
| O12-Te1-O10 | | | 89.0(3) | | | O12-Te1-O11 | | 178.8(3) | | | O12-Te1-O14 | | | 85.0(3) | |  |
| O13-Te1-O9 | | | 174.1(3) | | | O13-Te1-O10 | | 91.4(3) | | | O13-Te1-O11 | | | 93.9(3) | |  |
| O13-Te1-O12 | | | 85.3(3) | | | O13-Te1-O14 | | 88.9(3) | | | O14-Te1-O10 | | | 173.9(3) | |  |
| O14-Te1-O11 | | | 94.2(3) | | |  | |  | | |  | | |  | |  |
| **{Er_2_Te_2_W_34_}** | | | | | | | | | | | | | | | | |
| O1-Er1-O2 | | | 67.4(4) | | | O1-Er1-O3 | | 100.6(4) | | | O1-Er1-O4 | | | 137.5(3) | |  |
| O1-Er1-O6 | | | 87.9(3) | | | O1-Er1-O8#1 | | 76.2(3) | | | O2-Er1-O3 | | | 76.0(4) | |  |
| O4-Er1-O2 | | | 71.5(3) | | | O4-Er1-O3 | | 79.3(3) | | | O4-Er1-O6 | | | 74.7(3) | |  |
| O4-Er1-O8#1 | | | 139.1(3) | | | O5-Er1-O1 | | 149.4(3) | | | O5-Er1-O2 | | | 143.2(3) | |  |
| O5-Er1-O3 | | | 90.9(4) | | | O5-Er1-O4 | | 72.4(3) | | | O5-Er1-O6 | | | 96.7(3) | |  |
| O5-Er1-O7 | | | 75.1(3) | | | O5-Er1-O8#1 | | 81.3(3) | | | O6-Er1-O2 | | | 80.3(4) | |  |
| O6-Er1-O3 | | | 149.2(3) | | | O6-Er1-O8#1 | | 140.4(3) | | | O7-Er1-O1 | | | 79.0(3) | |  |
| O7-Er1-O2 | | | 133.5(3) | | | O7-Er1-O3 | | 143.7(3) | | | O7-Er1-O4 | | | 125.3(3) | |  |
| O7-Er1-O6 | | | 66.9(3) | | | O7-Er1-O8#1 | | 74.6(3) | | | O8#1-Er1-O2 | | | 123.8(3) | |  |
| O8#1-Er1-O3 | | | 70.2(3) | | | O9-Te1-O10 | | 90.9(3) | | | O9-Te1-O11 | | | 89.1(3) | |  |
| O9-Te1-O13 | | | 174.2(3) | | | O10-Te1-O11 | | 91.7(3) | | | O12-Te1-O9 | | | 92.5(3) | |  |
| O12-Te1-O10 | | | 94.6(3) | | | O12-Te1-O11 | | 173.4(3) | | | O12-Te1-O13 | | | 87.7(3) | |  |
| O13-Te1-O10 | | | 94.9(3) | | | O13-Te1-O11 | | 90.0(3) | | | O14-Te1-O9 | | | 89.2(3) | |  |
| O14-Te1-O10 | | | 179.5(3) | | | O14-Te1-O11 | | 88.7(3) | | | O14-Te1-O12 | | | 84.9(3) | |  |
| O14-Te1-O13 | | | 85.0(3) | | |  | |  | | |  | | |  | |  |
| **{Tm_2_Te_2_W_34_}** | | | | | | | | | | | | | | | | |
| O1-Tm1-O3 | | | 101.4(4) | | | O2-Tm1-O1 | | 67.7(5) | | | O2-Tm1-O3 | | | 75.4(4) | |  |
| O2-Tm1-O4 | | | 71.8(4) | | | O2-Tm1-O6 | | 81.8(4) | | | O2-Tm1-O7 | | | 134.1(4) | |  |
| O2-Tm1-O8#1 | | | 122.7(4) | | | O4-Tm1-O1 | | 137.3(4) | | | O4-Tm1-O3 | | | 80.5(4) | |  |
| O4-Tm1-O8#1 | | | 140.0(3) | | | O5-Tm1-O1 | | 148.1(4) | | | O5-Tm1-O2 | | | 144.2(4) | |  |
| O5-Tm1-O3 | | | 91.3(4) | | | O5-Tm1-O4 | | 73.3(3) | | | O5-Tm1-O6 | | | 96.4(3) | |  |
| O5-Tm1-O7 | | | 74.4(3) | | | O5-Tm1-O8#1 | | 81.0(4) | | | O6-Tm1-O1 | | | 87.0(4) | |  |
| O6-Tm1-O3 | | | 150.3(3) | | | O6-Tm1-O4 | | 74.4(3) | | | O6-Tm1-O8#1 | | | 139.7(3) | |  |
| O7-Tm1-O1 | | | 78.1(4) | | | O7-Tm1-O3 | | 143.1(4) | | | O7-Tm1-O4 | | | 124.9(3) | |  |
| O7-Tm1-O6 | | | 66.3(3) | | | O7-Tm1-O8#1 | | 74.4(3) | | | O8#1-Tm1-O1 | | | 76.3(4) | |  |
| O8#1-Tm1-O3 | | | 69.8(3) | | | O9-Te1-O10 | | 89.1(4) | | | O9-Te1-O11 | | | 90.0(4) | |  |
| O11-Te1-O10 | | | 86.1(4) | | | O12-Te1-O9 | | 174.0(3) | | | O12-Te1-O10 | | | 84.9(4) | |  |
| O12-Te1-O11 | | | 89.1(4) | | | O12-Te1-O13 | | 93.8(4) | | | O13-Te1-O9 | | | 92.2(4) | |  |
| O13-Te1-O10 | | | 178.7(4) | | | O13-Te1-O11 | | 94.3(4) | | | O14-Te1-O9 | | | 88.5(4) | |  |
| O14-Te1-O10 | | | 88.3(4) | | | O14-Te1-O11 | | 174.2(3) | | | O14-Te1-O12 | | | 91.9(4) | |  |
| O14-Te1-O13 | | | 91.4(4) | | |  | |  | | |  | | |  | |  |
| **{Yb_2_Te_2_W_34_}** | | | | | | | | | | | | | | | | |
| O1-Yb1-O3 | | | 101.1(4) | | | O2-Yb1-O1 | | 68.2(4) | | | O2-Yb1-O3 | | | 75.5(5) | |  |
| O2-Yb1-O4 | | | 71.3(4) | | | O2-Yb1-O6 | | 80.4(4) | | | O2-Yb1-O7 | | | 134.8(4) | |  |
| O2-Yb1-O8#1 | | | 123.7(4) | | | O4-Yb1-O1 | | 137.9(4) | | | O4-Yb1-O3 | | | 79.3(4) | |  |
| O4-Yb1-O8#1 | | | 139.1(3) | | | O5-Yb1-O1 | | 149.3(4) | | | O5-Yb1-O2 | | | 142.5(4) | |  |
| O5-Yb1-O3 | | | 90.7(4) | | | O5-Yb1-O4 | | 72.0(3) | | | O5-Yb1-O6 | | | 97.0(3) | |  |
| O5-Yb1-O7 | | | 74.7(3) | | | O5-Yb1-O8#1 | | 81.5(3) | | | O6-Yb1-O1 | | | 87.4(4) | |  |
| O6-Yb1-O3 | | | 149.0(4) | | | O6-Yb1-O4 | | 74.8(3) | | | O6-Yb1-O8#1 | | | 140.4(3) | |  |
| O7-Yb1-O1 | | | 79.1(4) | | | O7-Yb1-O3 | | 143.2(3) | | | O7-Yb1-O4 | | | 125.0(3) | |  |
| O7-Yb1-O6 | | | 67.4(3) | | | O7-Yb1-O8#1 | | 74.2(3) | | | O8#1-Yb1-O1 | | | 76.1(4) | |  |
| O8#1-Yb1-O3 | | | 70.4(3) | | | O9-Te1-O10 | | 91.6(4) | | | O9-Te1-O11 | | | 91.8(3) | |  |
| O11-Te1-O10 | | | 89.2(3) | | | O12-Te1-O9 | | 179.5(4) | | | O12-Te1-O10 | | | 88.6(4) | |  |
| O12-Te1-O11 | | | 88.6(3) | | | O12-Te1-O13 | | 85.1(4) | | | O13-Te1-O9 | | | 94.7(3) | |  |
| O13-Te1-O10 | | | 173.7(4) | | | O13-Te1-O11 | | 90.2(3) | | | O14-Te1-O9 | | | 94.0(3) | |  |
| O14-Te1-O10 | | | 91.7(4) | | | O14-Te1-O11 | | 174.1(3) | | | O14-Te1-O12 | | | 85.6(3) | |  |
| O14-Te1-O13 | | | 88.3(4) | | |  | |  | | |  | | |  | |  |
| **{Lu_2_Te_2_W_34_}** | | | | | | | | | | | | | | | | |
| O1-Lu1-O3 | | | 68.2(3) | | | O2-Lu1-O1 | | 100.2(3) | | | O2-Lu1-O3 | | | 75.4(3) | |  |
| O2-Lu1-O4 | | | 79.8(3) | | | O2-Lu1-O6 | | 91.6(3) | | | O2-Lu1-O7 | | | 143.6(3) | |  |
| O2-Lu1-O8#1 | | | 70.4(3) | | | O4-Lu1-O1 | | 137.6(3) | | | O4-Lu1-O3 | | | 70.9(3) | |  |
| O4-Lu1-O8#1 | | | 139.8(2) | | | O5-Lu1-O1 | | 88.4(3) | | | O5-Lu1-O2 | | | 149.0(3) | |  |
| O5-Lu1-O3 | | | 80.5(3) | | | O5-Lu1-O4 | | 74.1(2) | | | O5-Lu1-O6 | | | 96.3(2) | |  |
| O5-Lu1-O7 | | | 67.2(2) | | | O5-Lu1-O8#1 | | 140.4(2) | | | O6-Lu1-O1 | | | 148.7(3) | |  |
| O6-Lu1-O3 | | | 143.1(3) | | | O6-Lu1-O4 | | 72.9(2) | | | O6-Lu1-O8#1 | | | 81.4(2) | |  |
| O7-Lu1-O1 | | | 78.5(3) | | | O7-Lu1-O3 | | 133.9(3) | | | O7-Lu1-O4 | | | 125.6(2) | |  |
| O7-Lu1-O6 | | | 74.9(2) | | | O7-Lu1-O8#1 | | 74.2(2) | | | O8#1-Lu1-O1 | | | 75.5(3) | |  |
| O8#1-Lu1-O3 | | | 123.8(3) | | | O9-Te1-O10 | | 91.6(3) | | | O9-Te1-O11 | | | 91.7(2) | |  |
| O11-Te1-O10 | | | 88.7(3) | | | O12-Te1-O9 | | 94.3(3) | | | O12-Te1-O10 | | | 91.1(3) | |  |
| O12-Te1-O11 | | | 173.9(2) | | | O12-Te1-O13 | | 88.6(3) | | | O13-Te1-O9 | | | 94.3(3) | |  |
| O13-Te1-O10 | | | 174.1(2) | | | O13-Te1-O11 | | 91.0(3) | | | O14-Te1-O9 | | | 179.1(3) | |  |
| O14-Te1-O10 | | | 88.9(3) | | | O14-Te1-O11 | | 89.1(2) | | | O14-Te1-O12 | | | 84.9(2) | |  |
| O14-Te1-O13 | | | 85.2(3) | | |  | |  | | |  | | |  | |  |
| **{DyTeW_17_}** | | | | | | | | | | | | | | | | |
| O1-Dy1-O4 | | | 97.3(3) | | | O2-Dy1-O1 | | 76.7(3) | | | O2-Dy1-O4 | | | 69.7(2) | |  |
| O3-Dy1-O1 | | | 72.1(3) | | | O3-Dy1-O2 | | 127.1(3) | | | O3-Dy1-O4 | | | 73.2(3) | |  |
| O5-Dy1-O1 | | | 145.1(3) | | | O5-Dy1-O2 | | 129.9(2) | | | O5-Dy1-O3 | | | 73.4(2) | |  |
| O5-Dy1-O4 | | | 76.7(2) | | | O6-Dy1-O1 | | 142.9(3) | | | O6-Dy1-O2 | | | 76.9(2) | |  |
| O6-Dy1-O3 | | | 145.0(2) | | | O6-Dy1-O4 | | 97.4(2) | | | O6-Dy1-O5 | | | 71.6(2) | |  |
| O6-Dy1-O7 | | | 95.6(2) | | | O6-Dy1-O8 | | 69.0(2) | | | O7-Dy1-O1 | | | 93.2(3) | |  |
| O7-Dy1-O2 | | | 147.9(2) | | | O7-Dy1-O3 | | 76.0(2) | | | O7-Dy1-O4 | | | 142.3(2) | |  |
| O7-Dy1-O5 | | | 74.1(2) | | | O8-Dy1-O1 | | 79.0(3) | | | O8-Dy1-O2 | | | 74.0(2) | |  |
| O8-Dy1-O3 | | | 136.6(2) | | | O8-Dy1-O4 | | 143.4(2) | | | O8-Dy1-O5 | | | 125.8(2) | |  |
| O8-Dy1-O7 | | | 74.2(2) | | | O9-Te1-O10 | | 91.0(2) | | | O11-Te1-O9 | | | 88.6(2) | |  |
| O11-Te1-O10 | | | 92.1(2) | | | O11-Te1-O12 | | 173.9(2) | | | O12-Te1-O9 | | | 91.7(2) | |  |
| O12-Te1-O10 | | | 93.9(2) | | | O13-Te1-O9 | | 174.2(2) | | | O13-Te1-O10 | | | 94.7(2) | |  |
| O13-Te1-O11 | | | 90.3(2) | | | O13-Te1-O12 | | 88.8(2) | | | O14-Te1-O9 | | | 88.4(2) | |  |
| O14-Te1-O10 | | | 179.3(2) | | | O14-Te1-O11 | | 88.1(2) | | | O14-Te1-O12 | | | 85.8(2) | |  |
| O14-Te1-O13 | | | 86.0(2) | | |  | |  | | |  | | |  | |  |
| **{HoTeW_17_}** | | | | | | | | | | | | | | | | |
| O1-Ho1-O4 | | | 126.4(3) | | | O2-Ho1-O1 | | 76.8(4) | | | O2-Ho1-O4 | | | 71.1(4) | |  |
| O3-Ho1-O1 | | | 70.6(3) | | | O3-Ho1-O2 | | 97.7(4) | | | O3-Ho1-O4 | | | 72.6(3) | |  |
| O3-Ho1-O5 | | | 75.9(3) | | | O3-Ho1-O6 | | 141.6(3) | | | O3-Ho1-O7 | | | 97.4(3) | |  |
| O5-Ho1-O1 | | | 130.2(3) | | | O5-Ho1-O2 | | 144.5(3) | | | O5-Ho1-O4 | | | 73.8(3) | |  |
| O5-Ho1-O6 | | | 74.2(2) | | | O5-Ho1-O7 | | 71.1(3) | | | O6-Ho1-O1 | | | 147.8(3) | |  |
| O6-Ho1-O2 | | | 93.0(3) | | | O6-Ho1-O4 | | 76.3(3) | | | O7-Ho1-O1 | | | 77.8(3) | |  |
| O7-Ho1-O2 | | | 144.1(3) | | | O7-Ho1-O4 | | 144.8(3) | | | O7-Ho1-O6 | | | 95.1(3) | |  |
| O8-Ho1-O1 | | | 74.2(3) | | | O8-Ho1-O2 | | 79.7(3) | | | O8-Ho1-O3 | | | 144.3(3) | |  |
| O8-Ho1-O4 | | | 136.7(3) | | | O8-Ho1-O5 | | 125.5(3) | | | O8-Ho1-O6 | | | 73.9(3) | |  |
| O8-Ho1-O7 | | | 69.1(3) | | | O9-Te1-O10 | | 91.2(3) | | | O11-Te1-O9 | | | 92.3(3) | |  |
| O11-Te1-O10 | | | 88.9(3) | | | O12-Te1-O9 | | 93.6(3) | | | O12-Te1-O10 | | | 91.5(3) | |  |
| O12-Te1-O11 | | | 174.0(3) | | | O12-Te1-O14 | | 85.4(3) | | | O13-Te1-O9 | | | 94.1(3) | |  |
| O13-Te1-O10 | | | 174.6(3) | | | O13-Te1-O11 | | 89.9(3) | | | O13-Te1-O12 | | | 89.1(3) | |  |
| O13-Te1-O14 | | | 86.1(3) | | | O14-Te1-O9 | | 179.0(3) | | | O14-Te1-O10 | | | 88.7(3) | |  |
| O14-Te1-O11 | | | 88.7(3) | | |  | |  | | |  | | |  | |  |
| **{ErTeW_17_}** | | | | | | | | | | | | | | | | |
| O1-Er1-O4 | | | 97.6(6) | | | O2-Er1-O1 | | 76.8(6) | | | O2-Er1-O4 | | | 70.9(4) | |  |
| O2-Er1-O6 | | | 77.7(5) | | | O3-Er1-O1 | | 71.2(5) | | | O3-Er1-O2 | | | 126.3(5) | |  |
| O3-Er1-O4 | | | 71.9(5) | | | O3-Er1-O5 | | 73.2(4) | | | O3-Er1-O6 | | | 144.5(4) | |  |
| O3-Er1-O7 | | | 76.4(4) | | | O5-Er1-O1 | | 144.0(5) | | | O5-Er1-O2 | | | 130.9(5) | |  |
| O5-Er1-O4 | | | 76.2(4) | | | O5-Er1-O6 | | 71.4(4) | | | O5-Er1-O7 | | | 73.1(4) | |  |
| O6-Er1-O1 | | | 144.2(5) | | | O6-Er1-O4 | | 97.3(5) | | | O7-Er1-O1 | | | 93.3(5) | |  |
| O7-Er1-O2 | | | 148.1(4) | | | O7-Er1-O4 | | 140.9(4) | | | O7-Er1-O6 | | | 95.2(4) | |  |
| O8-Er1-O1 | | | 80.8(5) | | | O8-Er1-O2 | | 74.7(4) | | | O8-Er1-O3 | | | 137.4(4) | |  |
| O8-Er1-O4 | | | 145.0(4) | | | O8-Er1-O5 | | 124.3(4) | | | O8-Er1-O6 | | | 68.5(4) | |  |
| O8-Er1-O7 | | | 73.8(4) | | | O9-Te1-O10 | | 89.5(4) | | | O11-Te1-O9 | | | 86.4(4) | |  |
| O11-Te1-O10 | | | 86.4(4) | | | O12-Te1-O9 | | 174.9(4) | | | O12-Te1-O10 | | | 90.7(4) | |  |
| O12-Te1-O11 | | | 88.4(4) | | | O12-Te1-O14 | | 91.6(4) | | | O13-Te1-O9 | | | 90.0(4) | |  |
| O13-Te1-O10 | | | 174.4(4) | | | O13-Te1-O11 | | 88.1(4) | | | O13-Te1-O12 | | | 89.4(4) | |  |
| O13-Te1-O14 | | | 92.4(4) | | | O14-Te1-O9 | | 93.5(4) | | | O14-Te1-O10 | | | 93.2(4) | |  |
| O14-Te1-O11 | | | 179.6(4) | | |  | |  | | |  | | |  | |  |
| **{TmTeW_17_}** | | | | | | | | | | | | | | | | |
| O1-Tm1-O4 | | | 126.3(3) | | | O2-Tm1-O1 | | 77.9(3) | | | O2-Tm1-O4 | | | 71.1(3) | |  |
| O3-Tm1-O1 | | | 70.5(3) | | | O3-Tm1-O2 | | 98.1(3) | | | O3-Tm1-O4 | | | 71.9(3) | |  |
| O3-Tm1-O5 | | | 75.6(3) | | | O3-Tm1-O6 | | 141.9(2) | | | O3-Tm1-O7 | | | 97.2(3) | |  |
| O5-Tm1-O1 | | | 129.4(3) | | | O5-Tm1-O2 | | 144.4(3) | | | O5-Tm1-O4 | | | 73.7(3) | |  |
| O5-Tm1-O6 | | | 74.5(2) | | | O5-Tm1-O7 | | 71.9(2) | | | O6-Tm1-O1 | | | 147.6(2) | |  |
| O6-Tm1-O2 | | | 92.6(3) | | | O6-Tm1-O4 | | 77.4(3) | | | O7-Tm1-O1 | | | 76.3(3) | |  |
| O7-Tm1-O2 | | | 143.3(3) | | | O7-Tm1-O4 | | 145.5(3) | | | O7-Tm1-O6 | | | 95.5(2) | |  |
| O8-Tm1-O1 | | | 73.7(3) | | | O8-Tm1-O2 | | 79.3(3) | | | O8-Tm1-O3 | | | 77.4(3) | |  |
| O8-Tm1-O4 | | | 137.5(3) | | | O8-Tm1-O5 | | 126.1(2) | | | O8-Tm1-O6 | | | 74.2(2) | |  |
| O8-Tm1-O7 | | | 68.8(2) | | | O9-Te1-O10 | | 91.2(3) | | | O11-Te1-O9 | | | 91.7(3) | |  |
| O11-Te1-O10 | | | 88.4(3) | | | O12-Te1-O9 | | 179.5(2) | | | O12-Te1-O10 | | | 88.8(2) | |  |
| O12-Te1-O11 | | | 88.8(3) | | | O12-Te1-O14 | | 85.1(3) | | | O13-Te1-O9 | | | 94.3(2) | |  |
| O13-Te1-O10 | | | 174.4(2) | | | O13-Te1-O11 | | 90.4(2) | | | O13-Te1-O12 | | | 85.7(2) | |  |
| O13-Te1-O14 | | | 88.6(2) | | | O14-Te1-O9 | | 94.3(2) | | | O14-Te1-O10 | | | 92.0(3) | |  |
| O14-Te1-O11 | | | 173.9(3) | | |  | |  | | |  | | |  | |  |
| **{YbTeW_17_}** | | | | | | | | | | | | | | | | |
| O1-Yb1-O4 | | | 97.5(3) | | | O2-Yb1-O1 | | 75.8(3) | | | O2-Yb1-O4 | | | 70.3(2) | |  |
| O3-Yb1-O1 | | | 71.8(3) | | | O3-Yb1-O2 | | 125.5(3) | | | O3-Yb1-O4 | | | 72.0(3) | |  |
| O5-Yb1-O1 | | | 144.8(3) | | | O5-Yb1-O2 | | 130.4(3) | | | O5-Yb1-O3 | | | 73.3(3) | |  |
| O5-Yb1-O4 | | | 75.7(3) | | | O6-Yb1-O1 | | 143.4(3) | | | O6-Yb1-O2 | | | 77.5(3) | |  |
| O6-Yb1-O3 | | | 144.8(3) | | | O6-Yb1-O4 | | 96.7(3) | | | O6-Yb1-O5 | | | 69.6(2) | |  |
| O6-Yb1-O7 | | | 96.4(2) | | | O6-Yb1-O8 | | 69.6(2) | | | O7-Yb1-O1 | | | 93.2(3) | |  |
| O7-Yb1-O2 | | | 148.1(2) | | | O7-Yb1-O3 | | 76.6(2) | | | O7-Yb1-O4 | | | 141.5(2) | |  |
| O7-Yb1-O5 | | | 74.4(2) | | | O8-Yb1-O1 | | 79.2(3) | | | O8-Yb1-O2 | | | 74.2(2) | |  |
| O8-Yb1-O3 | | | 137.3(3) | | | O8-Yb1-O4 | | 143.9(3) | | | O8-Yb1-O5 | | | 126.2(2) | |  |
| O8-Yb1-O7 | | | 74.4(2) | | | O9-Te1-O10 | | 91.2(2) | | | O11-Te1-O9 | | | 92.2(2) | |  |
| O11-Te1-O10 | | | 88.8(2) | | | O11-Te1-O12 | | 173.5(2) | | | O12-Te1-O9 | | | 94.2(2) | |  |
| O12-Te1-O10 | | | 92.2(2) | | | O13-Te1-O9 | | 94.7(2) | | | O13-Te1-O10 | | | 174.1(2) | |  |
| O13-Te1-O11 | | | 89.9(2) | | | O13-Te1-O12 | | 88.5(2) | | | O14-Te1-O9 | | | 179.2(2) | |  |
| O14-Te1-O10 | | | 88.7(2) | | | O14-Te1-O11 | | 88.5(2) | | | O14-Te1-O12 | | | 85.1(2) | |  |
| O14-Te1-O13 | | | 85.5(2) | | |  | |  | | |  | | |  | |  |
| **{LuTeW_17_}** | | | | | | | | | | | | | | | | |
| O1-Lu1-O4 | | | 125.5(5) | | | O2-Lu1-O1 | | 71.9(5) | | | O2-Lu1-O4 | | | 70.0(5) | |  |
| O3-Lu1-O1 | | | 71.2(5) | | | O3-Lu1-O2 | | 97.4(5) | | | O3-Lu1-O4 | | | 76.7(5) | |  |
| O5-Lu1-O1 | | | 73.8(5) | | | O5-Lu1-O2 | | 76.1(5) | | | O5-Lu1-O3 | | | 144.7(5) | |  |
| O5-Lu1-O4 | | | 130.0(5) | | | O6-Lu1-O1 | | 77.2(5) | | | O6-Lu1-O2 | | | 142.1(5) | |  |
| O6-Lu1-O3 | | | 92.7(5) | | | O6-Lu1-O4 | | 147.7(5) | | | O6-Lu1-O5 | | | 74.7(4) | |  |
| O6-Lu1-O7 | | | 74.6(4) | | | O6-Lu1-O8 | | 95.8(4) | | | O7-Lu1-O1 | | | 138.0(5) | |  |
| O7-Lu1-O2 | | | 143.1(5) | | | O7-Lu1-O3 | | 79.7(5) | | | O7-Lu1-O4 | | | 73.6(5) | |  |
| O7-Lu1-O5 | | | 126.0(4) | | | O8-Lu1-O1 | | 145.0(5) | | | O8-Lu1-O2 | | | 97.1(5) | |  |
| O8-Lu1-O3 | | | 143.8(5) | | | O8-Lu1-O4 | | 77.5(5) | | | O8-Lu1-O5 | | | 71.2(4) | |  |
| O8-Lu1-O7 | | | 69.0(4) | | | O9-Te1-O10 | | 86.2(4) | | | O11-Te1-O9 | | | 90.2(4) | |  |
| O11-Te1-O10 | | | 88.1(4) | | | O11-Te1-O12 | | 89.2(5) | | | O12-Te1-O9 | | | 174.4(4) | |  |
| O12-Te1-O10 | | | 88.3(4) | | | O13-Te1-O9 | | 93.6(4) | | | O13-Te1-O10 | | | 179.5(4) | |  |
| O13-Te1-O11 | | | 92.4(4) | | | O13-Te1-O12 | | 92.0(4) | | | O14-Te1-O9 | | | 89.4(4) | |  |
| O14-Te1-O10 | | | 86.0(4) | | | O14-Te1-O11 | | 174.1(4) | | | O14-Te1-O12 | | | 90.7(4) | |  |
| O14-Te1-O13 | | | 93.5(4) | | |  | |  | | |  | | |  | |  |
| **{DyTeW_6_}** | | | | | | | | | | | | | | | | |
| O1-Dy1-O2#2 | | | 73.78(14) | | | O1-Dy1-O2 | | 144.46(15) | | | O1-Dy1-O2#3 | | | 74.70(14) | |  |
| O1-Dy1-O2#4 | | | 74.30(14) | | | O1-Dy1-O1#3 | | 177.2(2) | | | O1-Dy1-O1#2 | | | 139.7(2) | |  |
| O1-Dy1-O1#4 | | | 77.4(2) | | | O2-Dy1-O2#4 | | 139.29(19) | | | O2-Dy1-O2#3 | | | 116.34(19) | |  |
| O2-Dy1-O2#2 | | | 78.4(2) | | | O2#4-Dy1-O2#3 | | 78.4(2) | | | O2#3-Dy1-O2#2 | | | 139.29(19) | |  |
| O2#4-Dy1-O2#2 | | | 116.34(19) | | | O4-Te1-O4#5 | | 180.0 | | | O4-Te1-O3 | | | 85.04(14) | |  |
| O4-Te1-O3#5 | | | 94.96(14) | | | O4-Te1-O3#1 | | 94.96(14) | | | O4-Te1-O3#6 | | | 85.04(14) | |  |
| O3-Te1-O3#1 | | | 85.0(2) | | | O3-Te1-O3#5 | | 180.0(2) | | | O3-Te1-O3#6 | | | 95.0(2) | |  |
| O3#5-Te1-O3#1 | | | 95.0(2) | | | O3#6-Te1-O3#1 | | 180.0 | | | O3#5-Te1-O3#6 | | | 85.0(2) | |  |
| **{HoTeW_6_}** | | | | | | | | | | | | | | | | |
| O1-Ho1-O2#6 | | | 74.4(2) | | | O1-Ho1-O2 | | 74.6(2) | | | O1-Ho1-O2#5 | | | 73.8(2) | |  |
| O1-Ho1-O2#4 | | | 144.4(2) | | | O1-Ho1-O1#6 | | 139.6(3) | | | O1-Ho1-O1#5 | | | 77.0(4) | |  |
| O1-Ho1-O1#4 | | | 117.6(4) | | | O2-Ho1-O2#4 | | 116.2(3) | | | O2-Ho1-O2#6 | | | 78.4(3) | |  |
| O2-Ho1-O2#5 | | | 139.3(3) | | | O2#4-Ho1-O2#6 | | 139.3(3) | | | O2#6-Ho1-O2#5 | | | 116.2(3) | |  |
| O2#4-Ho1-O2#5 | | | 78.4(3) | | | O4-Te1-O3#3 | | 94.6(2) | | | O3#1-Te1-O3#2 | | | 180.0(2) | |  |
| O3-Te1-O3#2 | | | 84.9(3) | | | O4-Te1-O4#3 | | 180.0(5) | | | O3-Te1-O4 | | | 85.4(2) | |  |
| O3-Te1-O3#1 | | | 95.1(3) | | | O3#3-Te1-O3#1 | | 84.9(3) | | | O4-Te1-O3#1 | | | 85.4(2) | |  |
| O3-Te1-O3#3 | | | 180.0 | | | O3#3-Te1-O3#2 | | 95.1(3) | | | O4-Te1-O3#2 | | | 94.5(2) | |  |
| **{ErTeW_6_}** | | | | | | | | | | | | | | | | |
| O1-Er1-O2#4 | | | 74.37(14) | | | O1-Er1-O2 | | 75.23(14) | | | O1-Er1-O2#3 | | | 73.33(14) | |  |
| O1-Er1-O2#2 | | | 144.20(14) | | | O1-Er1-O1#3 | | 77.6(2) | | | O1-Er1-O1#2 | | | 116.3(2) | |  |
| O1-Er1-O1#4 | | | 140.49(19) | | | O2-Er1-O2#2 | | 116.41(19) | | | O2-Er1-O2#4 | | | 78.28(19) | |  |
| O2-Er1-O2#3 | | | 139.32(18) | | | O2#2-Er1-O2#3 | | 78.28(19) | | | O2#2-Er1-O2#4 | | | 139.32(18) | |  |
| O2#4-Er1-O2#3 | | | 116.41(19) | | | O4#6-Te1-O4#1 | | 180.0 | | | O3-Te1-O3#5 | | | 180.0 | |  |
| O3-Te1-O4#1 | | | 85.12(14) | | | O4-Te1-O4#1 | | 85.5(2) | | | O3-Te1-O4 | | | 94.89(14) | |  |
| O4-Te1-O4#5 | | | 180.0(16) | | | O4#5-Te1-O4#6 | | 85.5(2) | | | O4-Te1-O4#6 | | | 94.5(2) | |  |
| O3-Te1-O4#6 | | | 94.88(14) | | | O3-Te1-O4#5 | | 85.12(14) | | | O4#5-Te1-O4#1 | | | 94.5(2) | |  |
| **{TmTeW_6_}** | | | | | | | | | | | | | | | | |
| O1-Tm1-O2#3 | | | 74.67(17) | | | O1-Tm1-O2 | | 73.43(16) | | | O1-Tm1-O2#4 | | | 75.04(11) | |  |
| O1-Tm1-O2#2 | | | 143.88(16) | | | O1-Tm1-O1#2 | | 140.8(2) | | | O1-Tm1-O1#3 | | | 116.6(3) | |  |
| O1-Tm1-O1#4 | | | 77.2(3) | | | O2-Tm1-O2#4 | | 139.3(2) | | | O2-Tm1-O2#3 | | | 117.0(2) | |  |
| O2-Tm1-O2#2 | | | 77.8(2) | | | O2#2-Tm1-O2#3 | | 139.3(2) | | | O2#2-Tm1-O2#4 | | | 117.0(2) | |  |
| O2#3-Tm1-O2#4 | | | 77.8(2) | | | O3#6-Te1-O3#5 | | 94.4 (2) | | | O3-Te1-O3#5 | | | 85.6(2) | |  |
| O3-Te1-O3#1 | | | 94.4(2) | | | O4-Te1-O4#6 | | 180.0 | | | O3-Te1-O4 | | | 85.54(16) | |  |
| O4-Te1-O3#6 | | | 94.46(16) | | | O4-Te1-O3#5 | | 94.46(16) | | | O4-Te1-O3#1 | | | 85.54(16) | |  |
| O3#1-Te1-O3#5 | | | 180.0 | | | O3-Te1-O3#6 | | 180.0 | | | O3#6-Te1-O3#1 | | | 85.6(2) | |  |
| **{YbTeW_6_}** | | | | | | | | | | | | | | | | |
| O2-Yb1-O2#1 | | | 140.8(6) | | | O2-Yb1-O2#2 | | 115.5(6) | | | O2-Yb1-O2#3 | | | 78.1(6) | |  |
| O2-Yb1-O1#2 | | | 76.0(4) | | | O2-Yb1-O1#1 | | 73.9(4) | | | O2-Yb1-O1 | | | 143.3(4) | |  |
| O2-Yb1-O1#3 | | | 73.6(4) | | | O1-Yb1-O1#3 | | 141.0(5) | | | O1-Yb1-O1#2 | | | 116.7(7) | |  |
| O1-Yb1-O1#1 | | | 77.0(7) | | | O1#3-Yb1-O1#1 | | 116.7(7) | | | O1#2-Yb1-O1#1 | | | 141.0(5) | |  |
| O1#3-Yb1-O1#2 | | | 77.0(7) | | | O3-Te1-O3#4 | | 180.0 | | | O3-Te1-O3#6 | | | 85.3(6) | |  |
| O3-Te1-O3#5 | | | 94.7(6) | | | O4-Te1-O4#4 | | 180.0 | | | O3-Te1-O4 | | | 95.1(4) | |  |
| O4-Te1-O3#4 | | | 84.9(4) | | | O4-Te1-O3#5 | | 95.1(4) | | | O4-Te1-O3#6 | | | 84.9(4) | |  |
| O3#4-Te1-O3#6 | | | 94.7(6) | | | O3#4-Te1-O3#5 | | 85.3(6) | | | O3#5-Te1-O3#6 | | | 180.0(4) | |  |
| **{LuTeW_6_}** | | | | | | | | | | | | | | | |  |
| O1-Lu1-O1#1 | | | 140.9(5) | | | O1-Lu1-O1#2 | | 78.0(5) | | | O1-Lu1-O1#3 | | | 115.6(3) | |  |
| O1-Lu1-O2#3 | | | 75.5(3) | | | O1-Lu1-O2#1 | | 72.9(3) | | | O1-Lu1-O2 | | | 144.3(3) | |  |
| O1-Lu1-O2#2 | | | 74.4(3) | | | O2-Lu1-O2#3 | | 116.4(4) | | | O2-Lu1-O2#1 | | | 78.5(4) | |  |
| O2-Lu1-O2#2 | | | 139.0(5) | | | O2#2-Lu1-O2#3 | | 78.5(4) | | | O2#2-Lu1-O2#1 | | | 116.4(4) | |  |
| O2#3-Lu1-O2#1 | | | 139.0(5) | | | O3-Te1-O3#4 | | 180.0 | | | O3-Te1-O4#4 | | | 94.3(3) | |  |
| O3-Te1-O4 | | | 85.7(3) | | | O3-Te1-O4#6 | | 85.7(3) | | | O3-Te1-O4#5 | | | 94.3(3) | |  |
| O4-Te1-O4#4 | | | 180.0(4) | | | O4-Te1-O4#5 | | 84.8(5) | | | O4-Te1-O4#6 | | | 95.2(5) | |  |
| O4#5-Te1-O4#4 | | | 95.2(5) | | | O4#6-Te1-O4#5 | | 180.0 | | | O4#6-Te1-O4#4 | | | 84.8(5) | |  |
| {Dy_2_Te_2_W_34_}: ^1^1-X,1-Y,1-Z. {Ho_2_Te_2_W_34_}: ^1^1-X,1-Y,1-Z. {Er_2_Te_2_W_34_}: ^1^1-X,1-Y,1-Z. {Tm_2_Te_2_W_34_}: ^1^1-X,1-Y,1-Z. {Yb_2_Te_2_W_34_}: ^1^1-X,1-Y,1-Z. {Lu_2_Te_2_W_34_}: ^1^1-X,1-Y,1-Z. {DyTeW_6_}: ^1^+X,+Y,-Z; ^2^1/2-X,1/2-Y,+Z; ^3^1/2-X,1/2-Y,-Z; ^4^-X,+Y,1/2-Z; ^5^+X,-Y,1/2-Z; ^6^-X,-Y,+Z. {HoTeW_6_}: ^1^1/2-X,1/2-Y,+Z; ^2^+X,1-Y,1/2-Z; ^3^-X,1-Y,+Z; ^4^-X,+Y,1/2-Z; ^5^+X,+Y,-Z; ^6^1/2-X,1/2-Y,-Z. {ErTeW_6_}: ^1^1/2-X,1/2-Y,+Z; ^2^-X,-Y,+Z; ^3^+X,-Y,1/2-Z; ^4^-X,+Y,1/2-Z; ^5^1/2-X,1/2-Y,-Z; ^6^+X,+Y,-Z. {TmTeW_6_}: ^1^1/2-X,1/2-Y,+Z; ^2^-X,-Y,+Z; ^3^-X,+Y,1/2-Z; ^4^+X,-Y,1/2-Z; ^5^1/2-X,1/2-Y,-Z; ^6^+X,+Y,-Z. {YbTeW_6_}: ^1^1/2-X,1/2-Y,+Z; ^2^+X,-Y,1/2-Z; ^3^-X,+Y,1/2-Z; ^4^-X,-Y,+Z; ^5^1/2-X,1/2-Y,1-Z; ^6^+X,+Y,1-Z. {LuTeW_6_}: ^1^1-X,+Y,3/2-Z; ^2^+X,1-Y,3/2-Z; ^3^1-X,1-Y,+Z; ^4^1/2-X,1/2-Y,1-Z; ^5^1/2-X,1/2-Y,+Z; ^6^+X,+Y,1-Z. | | | | | | | | | | | | | | | | |
