# Supplementary material for: Assembly of Lanthanide-Containing Tungstotellurates(VI): Syntheses, Structures, and Catalytic Properties
Source: Front Chem. 2020 Nov 23;8:598961. doi: 10.3389/fchem.2020.598961 (PMC7719746; doi:10.3389/fchem.2020.598961)

checkCIF/PLATON report

You have not supplied any structure factors. As a result the full set of tests cannot be run.

THIS REPORT IS FOR GUIDANCE ONLY. IF USED AS PART OF A REVIEW PROCEDURE FOR PUBLICATION, IT SHOULD NOT REPLACE THE EXPERTISE OF AN EXPERIENCED CRYSTALLOGRAPHIC REFEREE.

No syntax errors found.   CIF dictionary   Interpreting this report

Datablock: compound_dimer_Dy

```
Bond precision:  N- C = 0.0261 A         Wavelength=0.71073

Cell:          a=14.0216(3)       b=14.4472(2)     c=23.5468(4)
               alpha=106.148(1)   beta=94.726(1)   gamma=110.584(2)
Temperature:   296 K
```

	Calculated	Reported
Volume	4202.26(15)	4202.26(14)
Space group	P -1	P -1
Hall group	-P 1	-P 1
Moiety formula	Dy2 O128 Te2 W34, 8(C2 N), 5(O) [+ solvent]	?
Sum formula	C16 Dy2 N8 O133 Te2 W34 [+ solvent]	C32 H192 Dy2 N16 O151 Te2 W34
Mr	9263.00	10048.64
Dx,g cm-3	3.660	3.970
Z	1	1
Mu (mm-1)	24.467	24.467
F000	3968.0	3968.0
F000’	3945.09	
h,k,lmax	20,21,34	20,20,34
Nref	28238	24685
Tmin,Tmax	0.004,0.087	0.004,0.087
Tmin’	0.000	

```
Correction method= # Reported T Limits: Tmin=0.004 Tmax=0.087
AbsCorr = MULTI-SCAN

Data completeness= 0.874          Theta(max)= 31.604

R(reflections)= 0.0477( 18635)    wR2(reflections)= 0.1222( 24685)

S = 1.066                   Npar= 883
```

The following ALERTS were generated. Each ALERT has the format
test-name_ALERT_alert-type_alert-level.
Click on the hyperlinks for more details of the test.

[IMAGE] Alert level B

```
PLAT097_ALERT_2_B Large Reported Max.  (Positive) Residual Density       7.57 eA-3
PLAT306_ALERT_2_B Isolated Oxygen Atom (H-atoms Missing ?) .......        O65 Check
PLAT306_ALERT_2_B Isolated Oxygen Atom (H-atoms Missing ?) .......        O66 Check
```

[IMAGE] Alert level C

```
DIFMX02_ALERT_1_C  The maximum difference density is > 0.1*ZMAX*0.75
            The relevant atom site should be identified.
PLAT094_ALERT_2_C Ratio of Maximum / Minimum Residual Density ....       2.68 Report
PLAT220_ALERT_2_C NonSolvent   Resd 1  O   Ueq(max)/Ueq(min) Range        3.3 Ratio
PLAT244_ALERT_4_C Low    'Solvent' Ueq as Compared to Neighbors of         N1 Check
PLAT244_ALERT_4_C Low    'Solvent' Ueq as Compared to Neighbors of         N2 Check
PLAT244_ALERT_4_C Low    'Solvent' Ueq as Compared to Neighbors of         N3 Check
```

[IMAGE] Alert level G

```
FORMU01_ALERT_2_G  There is a discrepancy between the atom counts in the
            _chemical_formula_sum and the formula from the _atom_site* data.
            Atom count from _chemical_formula_sum:C32 H192 Dy2 N16 O151 Te2 W34
            Atom count from the _atom_site data:  C16 Dy2 N8 O133 Te2 W34
CELLZ01_ALERT_1_G Difference between formula and atom_site contents detected.
CELLZ01_ALERT_1_G ALERT: Large difference may be due to a
            symmetry error - see SYMMG tests
           From the CIF: _cell_formula_units_Z    1
           From the CIF: _chemical_formula_sum  C32 H192 Dy2 N16 O151 Te2 W34
           TEST: Compare cell contents of formula and atom_site data
```

atom	Z*formula	cif sites	diff
C	32.00	16.00	16.00
H	192.00	0.00	192.00
Dy	2.00	2.00	0.00
N	16.00	8.00	8.00
O	151.00	133.00	18.00
Te	2.00	2.00	0.00
W	34.00	34.00	0.00

```
PLAT003_ALERT_2_G Number of Uiso or Uij Restrained non-H Atoms ...          2 Report
PLAT012_ALERT_1_G N.O.K.   _shelx_res_checksum Found in CIF ......     Please Check
PLAT040_ALERT_1_G No H-atoms in this Carbon Containing Compound ..     Please Check
PLAT041_ALERT_1_G Calc. and Reported SumFormula    Strings  Differ     Please Check
PLAT066_ALERT_1_G Predicted and Reported Tmin&Tmax Range Identical          ? Check
PLAT083_ALERT_2_G SHELXL Second Parameter in WGHT  Unusually Large      23.78 Why ?
PLAT186_ALERT_4_G The CIF-Embedded .res File Contains ISOR Records          3 Report
PLAT300_ALERT_4_G Atom Site Occupancy of O67       Constrained at         0.5 Check
PLAT302_ALERT_4_G Anion/Solvent/Minor-Residue Disorder (Resd  8  )       100% Note
PLAT304_ALERT_4_G Non-Integer Number of Atoms in ..... (Resd  8  )       0.50 Check
PLAT311_ALERT_2_G Isolated Disordered Oxygen Atom (No H's ?) .....        O67 Check
PLAT606_ALERT_4_G Solvent Accessible VOID(S) in Structure ........          ! Info
PLAT794_ALERT_5_G Tentative Bond Valency for W1       (VI)      .        6.26 Info
PLAT794_ALERT_5_G Tentative Bond Valency for W2       (VI)      .        6.16 Info
PLAT794_ALERT_5_G Tentative Bond Valency for W3       (VI)      .        6.11 Info
PLAT794_ALERT_5_G Tentative Bond Valency for W4       (VI)      .        6.10 Info
PLAT794_ALERT_5_G Tentative Bond Valency for W5       (VI)      .        6.25 Info
PLAT794_ALERT_5_G Tentative Bond Valency for W6       (VI)      .        6.16 Info
PLAT794_ALERT_5_G Tentative Bond Valency for W7       (VI)      .        6.18 Info
PLAT794_ALERT_5_G Tentative Bond Valency for W8       (VI)      .        6.07 Info
PLAT794_ALERT_5_G Tentative Bond Valency for W9       (VI)      .        6.26 Info
```

```
PLAT794_ALERT_5_G Tentative Bond Valency for W10       (VI)      .       6.19 Info
PLAT794_ALERT_5_G Tentative Bond Valency for W11       (VI)      .       6.08 Info
PLAT794_ALERT_5_G Tentative Bond Valency for W12       (VI)      .       6.15 Info
PLAT794_ALERT_5_G Tentative Bond Valency for W13       (VI)      .       6.20 Info
PLAT794_ALERT_5_G Tentative Bond Valency for W14       (VI)      .       6.18 Info
PLAT794_ALERT_5_G Tentative Bond Valency for W15       (VI)      .       6.21 Info
PLAT794_ALERT_5_G Tentative Bond Valency for W16       (VI)      .       6.17 Info
PLAT794_ALERT_5_G Tentative Bond Valency for W17       (VI)      .       6.07 Info
PLAT794_ALERT_5_G Tentative Bond Valency for Dy1       (III)     .       3.05 Info
PLAT794_ALERT_5_G Tentative Bond Valency for Te1       (VI)      .       5.37 Info
PLAT860_ALERT_3_G Number of Least-Squares Restraints .............         12 Note
PLAT868_ALERT_4_G ALERTS Due to the Use of _smtbx_masks Suppressed          ! Info
PLAT933_ALERT_2_G Number of OMIT Records in Embedded .res File ...          1 Note
PLAT941_ALERT_3_G Average HKL Measurement Multiplicity ...........        2.2 Low
```

---

 0 **ALERT level A** = Most likely a serious problem - resolve or explain
 3 **ALERT level B** = A potentially serious problem, consider carefully
 6 **ALERT level C** = Check. Ensure it is not caused by an omission or oversight
38 **ALERT level G** = General information/check it is not something unexpected

 7 ALERT type 1 CIF construction/syntax error, inconsistent or missing data
10 ALERT type 2 Indicator that the structure model may be wrong or deficient
 2 ALERT type 3 Indicator that the structure quality may be low
 9 ALERT type 4 Improvement, methodology, query or suggestion
19 ALERT type 5 Informative message, check

Datablock: compound_dimer_Ho

```
Bond precision:  N- C = 0.0250 A         Wavelength=0.71073

Cell:          a=14.0253(3)        b=14.4358(3)      c=23.5321(4)
               alpha=106.1507(18)  beta=94.6938(17)  gamma=110.605(2)
Temperature:  296 K
```

	Calculated	Reported
Volume	4197.21(17)	4197.21(17)
Space group	P -1	P -1
Hall group	-P 1	-P 1
Moiety formula	Ho2 O128 Te2 W34, 6(C2 N), 2(C2 N0.50), 4.5(O) [+ solvent]	?
Sum formula	C16 Ho2 N7 O132.50 Te2 W34 [+ solvent]	C30 H187 Ho2 N15 O152 Te2 W34
Mr	9245.85	10026.44
Dx,g cm-3	3.658	3.966
Z	1	1
Mu (mm-1)	24.548	24.548
F000	3959.0	3959.0
F000’	3936.07	
h,k,lmax	20,21,34	20,20,34
Nref	28150	24778
Tmin,Tmax	0.004,0.086	0.004,0.086
Tmin’	0.000	

Correction method= # Reported T Limits: Tmin=0.004 Tmax=0.086
AbsCorr = MULTI-SCAN

Data completeness= 0.880 Theta(max)= 31.585

R(reflections)= 0.0494(17357) wR2(reflections)= 0.1106(24778)

S = 1.053 Npar= 883

The following ALERTS were generated. Each ALERT has the format
test-name_ALERT_alert-type_alert-level.
Click on the hyperlinks for more details of the test.

[IMAGE] **Alert level B**

PLAT306_ALERT_2_B Isolated Oxygen Atom (H-atoms Missing ?) .... O65 Check

---

[IMAGE] **Alert level C**

PLAT094_ALERT_2_C Ratio of Maximum / Minimum Residual Density .... 2.57 Report
PLAT213_ALERT_2_C Atom O55 has ADP max/min Ratio ..... 3.3 oblate
PLAT220_ALERT_2_C NonSolvent Resd 1 O Ueq(max)/Ueq(min) Range 3.1 Ratio
PLAT244_ALERT_4_C Low ’Solvent’ Ueq as Compared to Neighbors of N3 Check

---

[IMAGE] **Alert level G**

FORMU01_ALERT_2_G There is a discrepancy between the atom counts in the
_chemical_formula_sum and the formula from the _atom_site* data.
Atom count from _chemical_formula_sum:C30 H187 Ho2 N15 O152 Te2 W34
Atom count from the _atom_site data: C16 Ho2 N7 O132.5 Te2 W34
CELLZ01_ALERT_1_G Difference between formula and atom_site contents detected.
CELLZ01_ALERT_1_G ALERT: Large difference may be due to a
symmetry error - see SYMMG tests

From the CIF: _cell_formula_units_Z 1
From the CIF: _chemical_formula_sum C30 H187 Ho2 N15 O152 Te2 W34
TEST: Compare cell contents of formula and atom_site data

atom	Z*formula	cif sites	diff
C	30.00	16.00	14.00
H	187.00	0.00	187.00
Ho	2.00	2.00	0.00
N	15.00	7.00	8.00
O	152.00	132.50	19.50
Te	2.00	2.00	0.00
W	34.00	34.00	0.00

```
PLAT003_ALERT_2_G Number of Uiso or Uij Restrained non-H Atoms ...         19 Report
PLAT012_ALERT_1_G N.O.K.   _shelx_res_checksum Found in CIF ......     Please Check
PLAT040_ALERT_1_G No H-atoms in this Carbon Containing Compound ..     Please Check
PLAT041_ALERT_1_G Calc. and Reported SumFormula    Strings  Differ     Please Check
PLAT066_ALERT_1_G Predicted and Reported Tmin&Tmax Range Identical          ? Check
PLAT083_ALERT_2_G SHELXL Second Parameter in WGHT  Unusually Large      22.26 Why ?
PLAT178_ALERT_4_G The CIF-Embedded .res File Contains SIMU Records          1 Report
PLAT186_ALERT_4_G The CIF-Embedded .res File Contains ISOR Records          4 Report
PLAT300_ALERT_4_G Atom Site Occupancy of N4         Constrained at        0.5 Check
PLAT300_ALERT_4_G Atom Site Occupancy of O66        Constrained at       0.75 Check
PLAT300_ALERT_4_G Atom Site Occupancy of O67        Constrained at        0.5 Check
PLAT302_ALERT_4_G Anion/Solvent/Minor-Residue Disorder (Resd  5  )        20% Note
PLAT302_ALERT_4_G Anion/Solvent/Minor-Residue Disorder (Resd  7  )       100% Note
PLAT302_ALERT_4_G Anion/Solvent/Minor-Residue Disorder (Resd  8  )       100% Note
PLAT304_ALERT_4_G Non-Integer Number of Atoms in ..... (Resd  5  )       2.50 Check
PLAT304_ALERT_4_G Non-Integer Number of Atoms in ..... (Resd  7  )       0.75 Check
PLAT304_ALERT_4_G Non-Integer Number of Atoms in ..... (Resd  8  )       0.50 Check
PLAT311_ALERT_2_G Isolated Disordered Oxygen Atom (No H’s ?) .....        O66 Check
PLAT311_ALERT_2_G Isolated Disordered Oxygen Atom (No H’s ?) .....        O67 Check
PLAT606_ALERT_4_G Solvent Accessible VOID(S) in Structure ........          ! Info
PLAT720_ALERT_4_G Number of Unusual/Non-Standard Labels ..........          1 Note
PLAT790_ALERT_4_G Centre of Gravity not Within Unit Cell: Resd.  #          3 Note
              C2 N
PLAT794_ALERT_5_G Tentative Bond Valency for W1        (VI)      .       6.14 Info
PLAT794_ALERT_5_G Tentative Bond Valency for W2        (VI)      .       6.17 Info
PLAT794_ALERT_5_G Tentative Bond Valency for W3        (VI)      .       6.24 Info
PLAT794_ALERT_5_G Tentative Bond Valency for W4        (VI)      .       6.17 Info
PLAT794_ALERT_5_G Tentative Bond Valency for W5        (VI)      .       6.11 Info
PLAT794_ALERT_5_G Tentative Bond Valency for W6        (VI)      .       6.09 Info
PLAT794_ALERT_5_G Tentative Bond Valency for W7        (VI)      .       6.23 Info
PLAT794_ALERT_5_G Tentative Bond Valency for W8        (VI)      .       6.08 Info
PLAT794_ALERT_5_G Tentative Bond Valency for W9        (VI)      .       6.12 Info
PLAT794_ALERT_5_G Tentative Bond Valency for W10       (VI)      .       6.13 Info
PLAT794_ALERT_5_G Tentative Bond Valency for W11       (VI)      .       6.15 Info
PLAT794_ALERT_5_G Tentative Bond Valency for W12       (VI)      .       6.28 Info
PLAT794_ALERT_5_G Tentative Bond Valency for W13       (VI)      .       6.14 Info
PLAT794_ALERT_5_G Tentative Bond Valency for W14       (VI)      .       6.17 Info
PLAT794_ALERT_5_G Tentative Bond Valency for W15       (VI)      .       6.21 Info
PLAT794_ALERT_5_G Tentative Bond Valency for W16       (VI)      .       6.12 Info
PLAT794_ALERT_5_G Tentative Bond Valency for W17       (VI)      .       6.28 Info
PLAT794_ALERT_5_G Tentative Bond Valency for Ho1       (III)     .       3.33 Info
PLAT794_ALERT_5_G Tentative Bond Valency for Te1       (VI)      .       5.45 Info
PLAT860_ALERT_3_G Number of Least-Squares Restraints .............        102 Note
PLAT868_ALERT_4_G ALERTS Due to the Use of _smtbx_masks Suppressed          ! Info
PLAT933_ALERT_2_G Number of OMIT Records in Embedded .res File ...          4 Note
PLAT941_ALERT_3_G Average HKL Measurement Multiplicity ...........        2.3 Low
```

---

0 **ALERT level A** = Most likely a serious problem - resolve or explain
1 **ALERT level B** = A potentially serious problem, consider carefully

4 **ALERT level C** = Check. Ensure it is not caused by an omission or oversight
48 **ALERT level G** = General information/check it is not something unexpected

6 ALERT type 1 CIF construction/syntax error, inconsistent or missing data
10 ALERT type 2 Indicator that the structure model may be wrong or deficient
2 ALERT type 3 Indicator that the structure quality may be low
16 ALERT type 4 Improvement, methodology, query or suggestion
19 ALERT type 5 Informative message, check

Datablock: compound_dimer_Er

Bond precision: N- C = 0.0275 A Wavelength=0.71073

Cell:	a=14.0161(3)	b=14.4504(2)	c=23.5104(3)
	alpha=106.196(1)	beta=94.686(1)	gamma=110.566(2)
Temperature:	296 K		

	Calculated	Reported
Volume	4194.99(14)	4194.98(13)
Space group	P -1	P -1
Hall group	-P 1	-P 1
Moiety formula	Er2 O128 Te2 W34, 8(C2 N), 4(O) [+ solvent]	?
Sum formula	C16 Er2 N8 O132 Te2 W34 [+ solvent]	C30 H189 Er2 N15 O153 Te2 W34
Mr	9256.52	10049.11
Dx,g cm-3	3.664	3.978
Z	1	1
Mu (mm-1)	24.619	24.619
F000	3964.0	3964.0
F000’	3941.01	
h,k,lmax	20,21,34	20,20,33
Nref	27769	24370
Tmin,Tmax	0.004,0.085	0.004,0.085
Tmin’	0.000	

Correction method= # Reported T Limits: Tmin=0.004 Tmax=0.085
AbsCorr = MULTI-SCAN

Data completeness= 0.878 Theta(max)= 31.428

R(reflections)= 0.0503(19388) wR2(reflections)= 0.1374(24370)

S = 1.064 Npar= 874

The following ALERTS were generated. Each ALERT has the format
test-name_ALERT_alert-type_alert-level.
Click on the hyperlinks for more details of the test.

[IMAGE] **Alert level B**

PLAT306_ALERT_2_B Isolated Oxygen Atom (H-atoms Missing ?) .... O65 Check
PLAT306_ALERT_2_B Isolated Oxygen Atom (H-atoms Missing ?) .... O66 Check

---

[IMAGE] **Alert level C**

PLAT220_ALERT_2_C NonSolvent Resd 1 O Ueq(max)/Ueq(min) Range 3.1 Ratio
PLAT244_ALERT_4_C Low 'Solvent' Ueq as Compared to Neighbors of N1 Check
PLAT244_ALERT_4_C Low 'Solvent' Ueq as Compared to Neighbors of N3 Check

---

[IMAGE] **Alert level G**

FORMU01_ALERT_2_G There is a discrepancy between the atom counts in the
_chemical_formula_sum and the formula from the _atom_site* data.
Atom count from _chemical_formula_sum:C30 H189 Er2 N15 O153 Te2 W34
Atom count from the _atom_site data: C16 Er2 N8 O132 Te2 W34
CELLZ01_ALERT_1_G Difference between formula and atom_site contents detected.
CELLZ01_ALERT_1_G ALERT: Large difference may be due to a
symmetry error - see SYMMG tests
From the CIF: _cell_formula_units_Z 1
From the CIF: _chemical_formula_sum C30 H189 Er2 N15 O153 Te2 W34
TEST: Compare cell contents of formula and atom_site data

atom	Z*formula	cif sites	diff
C	30.00	16.00	14.00
H	189.00	0.00	189.00
Er	2.00	2.00	0.00
N	15.00	8.00	7.00
O	153.00	132.00	21.00
Te	2.00	2.00	0.00
W	34.00	34.00	0.00

PLAT003_ALERT_2_G Number of Uiso or Uij Restrained non-H Atoms ... 11 Report
PLAT012_ALERT_1_G N.O.K. _shelx_res_checksum Found in CIF ...... Please Check
PLAT040_ALERT_1_G No H-atoms in this Carbon Containing Compound .. Please Check
PLAT041_ALERT_1_G Calc. and Reported SumFormula Strings Differ Please Check
PLAT066_ALERT_1_G Predicted and Reported Tmin&Tmax Range Identical ? Check
PLAT083_ALERT_2_G SHELXL Second Parameter in WGHT Unusually Large 55.28 Why ?
PLAT186_ALERT_4_G The CIF-Embedded .res File Contains ISOR Records 6 Report
PLAT606_ALERT_4_G Solvent Accessible VOID(S) in Structure .... ! Info
PLAT794_ALERT_5_G Tentative Bond Valency for W1 (VI) . 6.07 Info
PLAT794_ALERT_5_G Tentative Bond Valency for W2 (VI) . 6.18 Info
PLAT794_ALERT_5_G Tentative Bond Valency for W3 (VI) . 6.26 Info
PLAT794_ALERT_5_G Tentative Bond Valency for W4 (VI) . 6.16 Info
PLAT794_ALERT_5_G Tentative Bond Valency for W5 (VI) . 6.17 Info
PLAT794_ALERT_5_G Tentative Bond Valency for W6 (VI) . 6.21 Info
PLAT794_ALERT_5_G Tentative Bond Valency for W7 (VI) . 6.07 Info
PLAT794_ALERT_5_G Tentative Bond Valency for W8 (VI) . 6.25 Info
PLAT794_ALERT_5_G Tentative Bond Valency for W9 (VI) . 6.22 Info
PLAT794_ALERT_5_G Tentative Bond Valency for W10 (VI) . 6.17 Info
PLAT794_ALERT_5_G Tentative Bond Valency for W11 (VI) . 6.18 Info
PLAT794_ALERT_5_G Tentative Bond Valency for W12 (VI) . 6.21 Info
PLAT794_ALERT_5_G Tentative Bond Valency for W13 (VI) . 6.35 Info
PLAT794_ALERT_5_G Tentative Bond Valency for W14 (VI) . 6.07 Info
PLAT794_ALERT_5_G Tentative Bond Valency for W15 (VI) . 6.07 Info
PLAT794_ALERT_5_G Tentative Bond Valency for W16 (VI) . 6.01 Info
PLAT794_ALERT_5_G Tentative Bond Valency for W17 (VI) . 6.13 Info
PLAT794_ALERT_5_G Tentative Bond Valency for Er1 (III) . 3.13 Info

```
PLAT794_ALERT_5_G Tentative Bond Valency for Te1       (VI)      .       5.48 Info
PLAT860_ALERT_3_G Number of Least-Squares Restraints .............         66 Note
PLAT868_ALERT_4_G ALERTS Due to the Use of _smtbx_masks Suppressed          ! Info
PLAT933_ALERT_2_G Number of OMIT Records in Embedded .res File ...          4 Note
PLAT941_ALERT_3_G Average HKL Measurement Multiplicity ...........        2.1 Low
```

---

0 **ALERT level A** = Most likely a serious problem - resolve or explain
2 **ALERT level B** = A potentially serious problem, consider carefully
3 **ALERT level C** = Check. Ensure it is not caused by an omission or oversight
34 **ALERT level G** = General information/check it is not something unexpected

6 ALERT type 1 CIF construction/syntax error, inconsistent or missing data
7 ALERT type 2 Indicator that the structure model may be wrong or deficient
2 ALERT type 3 Indicator that the structure quality may be low
5 ALERT type 4 Improvement, methodology, query or suggestion
19 ALERT type 5 Informative message, check

Datablock: compound_dimer_Tm

Bond precision: N- C = 0.0337 A   Wavelength=0.71073

Cell:	a=14.1499(5)	b=14.5884(5)	c=23.7181(7)
	alpha=106.195(3)	beta=94.601(3)	gamma=110.596(3)
Temperature:	296 K		

	Calculated	Reported
Volume	4313.7(3)	4313.7(3)
Space group	P -1	P -1
Hall group	-P 1	-P 1
Moiety formula	O128 Te2 Tm2 W34, 8(C2 N) [+ solvent]	?
Sum formula	C16 N8 O128 Te2 Tm2 W34 [+ solvent]	C30 H195 N15 O156 Te2 Tm2 W34
Mr	9195.86	10106.50
Dx,g cm-3	3.540	3.890
Z	1	1
Mu (mm-1)	23.994	23.994
F000	3934.0	3934.0
F000'	3910.95	
h,k,lmax	17,17,28	17,17,28
Nref	16061	16039
Tmin,Tmax	0.004,0.091	0.004,0.091
Tmin'	0.001	

Correction method= # Reported T Limits: Tmin=0.004 Tmax=0.091
AbsCorr = MULTI-SCAN

Data completeness= 0.999   Theta(max)= 25.500

```
R(reflections)= 0.0463( 12441)    wR2(reflections)= 0.1226( 16039)

S = 1.070                    Npar= 856
```

The following ALERTS were generated. Each ALERT has the format
test-name_ALERT_alert-type_alert-level.
Click on the hyperlinks for more details of the test.

[IMAGE] **Alert level C**

```
PLAT220_ALERT_2_C NonSolvent   Resd 1  O   Ueq(max)/Ueq(min) Range        3.3 Ratio
PLAT242_ALERT_2_C Low    'MainMol' Ueq as Compared to Neighbors of        Tm1 Check
```

[IMAGE] **Alert level G**

```
FORMU01_ALERT_2_G  There is a discrepancy between the atom counts in the
            _chemical_formula_sum and the formula from the _atom_site* data.
            Atom count from _chemical_formula_sum:C30 H195 N15 O156 Te2 Tm2 W34
            Atom count from the _atom_site data:  C16 N8 O128 Te2 Tm2 W34
CELLZ01_ALERT_1_G Difference between formula and atom_site contents detected.
CELLZ01_ALERT_1_G ALERT: Large difference may be due to a
            symmetry error - see SYMMG tests
           From the CIF: _cell_formula_units_Z    1
           From the CIF: _chemical_formula_sum  C30 H195 N15 O156 Te2 Tm2 W34
           TEST: Compare cell contents of formula and atom_site data

           atom    Z*formula  cif sites diff
           C         30.00     16.00   14.00
           H        195.00      0.00  195.00
           N         15.00      8.00    7.00
           O        156.00    128.00   28.00
           Te         2.00      2.00    0.00
           Tm         2.00      2.00    0.00
           W         34.00     34.00    0.00
PLAT003_ALERT_2_G Number of Uiso or Uij Restrained non-H Atoms ...         11 Report
PLAT012_ALERT_1_G N.O.K.   _shelx_res_checksum Found in CIF ......     Please Check
PLAT040_ALERT_1_G No H-atoms in this Carbon Containing Compound ..     Please Check
PLAT041_ALERT_1_G Calc. and Reported SumFormula    Strings  Differ     Please Check
PLAT066_ALERT_1_G Predicted and Reported Tmin&Tmax Range Identical          ? Check
PLAT154_ALERT_1_G The s.u.'s on the Cell Angles are Equal ..(Note)      0.003 Degree
PLAT186_ALERT_4_G The CIF-Embedded .res File Contains ISOR Records          5 Report
PLAT606_ALERT_4_G Solvent Accessible VOID(S) in Structure ........          ! Info
PLAT794_ALERT_5_G Tentative Bond Valency for W1       (VI)      .        5.90 Info
PLAT794_ALERT_5_G Tentative Bond Valency for W2       (VI)      .        5.89 Info
PLAT794_ALERT_5_G Tentative Bond Valency for W3       (VI)      .        5.91 Info
PLAT794_ALERT_5_G Tentative Bond Valency for W4       (VI)      .        5.81 Info
PLAT794_ALERT_5_G Tentative Bond Valency for W5       (VI)      .        5.91 Info
PLAT794_ALERT_5_G Tentative Bond Valency for W6       (VI)      .        6.01 Info
PLAT794_ALERT_5_G Tentative Bond Valency for W7       (VI)      .        5.76 Info
PLAT794_ALERT_5_G Tentative Bond Valency for W8       (VI)      .        5.80 Info
PLAT794_ALERT_5_G Tentative Bond Valency for W9       (VI)      .        5.95 Info
PLAT794_ALERT_5_G Tentative Bond Valency for W10      (VI)      .        5.99 Info
PLAT794_ALERT_5_G Tentative Bond Valency for W11      (VI)      .        5.96 Info
PLAT794_ALERT_5_G Tentative Bond Valency for W12      (VI)      .        5.89 Info
PLAT794_ALERT_5_G Tentative Bond Valency for W13      (VI)      .        5.81 Info
PLAT794_ALERT_5_G Tentative Bond Valency for W14      (VI)      .        5.98 Info
PLAT794_ALERT_5_G Tentative Bond Valency for W15      (VI)      .        5.73 Info
PLAT794_ALERT_5_G Tentative Bond Valency for W16      (VI)      .        5.84 Info
PLAT794_ALERT_5_G Tentative Bond Valency for W17      (VI)      .        5.82 Info
PLAT794_ALERT_5_G Tentative Bond Valency for Tm1      (III)     .        3.07 Info
```

```
PLAT794_ALERT_5_G Tentative Bond Valency for Te1       (VI)      .       5.07 Info
PLAT860_ALERT_3_G Number of Least-Squares Restraints .............         60 Note
PLAT868_ALERT_4_G ALERTS Due to the Use of _smtbx_masks Suppressed          ! Info
PLAT933_ALERT_2_G Number of OMIT Records in Embedded .res File ...         12 Note
PLAT941_ALERT_3_G Average HKL Measurement Multiplicity ...........        2.4 Low
```

---

0 **ALERT level A** = Most likely a serious problem - resolve or explain
0 **ALERT level B** = A potentially serious problem, consider carefully
2 **ALERT level C** = Check. Ensure it is not caused by an omission or oversight
34 **ALERT level G** = General information/check it is not something unexpected

7 ALERT type 1 CIF construction/syntax error, inconsistent or missing data
5 ALERT type 2 Indicator that the structure model may be wrong or deficient
2 ALERT type 3 Indicator that the structure quality may be low
3 ALERT type 4 Improvement, methodology, query or suggestion
19 ALERT type 5 Informative message, check

Datablock: compound_dimer_Yb

Bond precision: N- C = 0.0283 A Wavelength=0.71073

Cell:	a=14.0328(3)	b=14.4419(3)	c=23.4762(4)
	alpha=106.219(1)	beta=94.579(1)	gamma=110.586(2)
Temperature:	296 K		

	Calculated	Reported
Volume	4191.91(16)	4191.90(15)
Space group	P -1	P -1
Hall group	-P 1	-P 1
Moiety formula	O128 Te2 W34 Yb2, 4(C1.50 N), 4(C2 N), 4.4(O) [+ solvent]	?
Sum formula	C14 N8 O132.40 Te2 W34 Yb2 [+ solvent]	C30 H195 N15 O156 Te2 W34 Yb2
Mr	9250.46	10114.70
Dx,g cm-3	3.664	4.006
Z	1	1
Mu (mm-1)	24.751	24.751
F000	3959.2	3959.0
F000’	3936.04	
h,k,lmax	20,21,34	20,20,34
Nref	27980	24399
Tmin,Tmax	0.004,0.084	0.004,0.084
Tmin’	0.000	

Correction method= # Reported T Limits: Tmin=0.004 Tmax=0.084
AbsCorr = MULTI-SCAN

Data completeness= 0.872 Theta(max)= 31.525

R(reflections)= 0.0533(18492) wR2(reflections)= 0.1400(24399)

S = 1.072 Npar= 883

The following ALERTS were generated. Each ALERT has the format
test-name_ALERT_alert-type_alert-level.
Click on the hyperlinks for more details of the test.

[IMAGE] **Alert level B**

```
PLAT306_ALERT_2_B Isolated Oxygen Atom (H-atoms Missing ?) .......        O65 Check
```

[IMAGE] **Alert level C**

```
PLAT220_ALERT_2_C NonSolvent   Resd 1  O   Ueq(max)/Ueq(min) Range        3.7 Ratio
PLAT244_ALERT_4_C Low    'Solvent' Ueq as Compared to Neighbors of         N2 Check
```

[IMAGE] **Alert level G**

```
FORMU01_ALERT_2_G  There is a discrepancy between the atom counts in the
            _chemical_formula_sum and the formula from the _atom_site* data.
            Atom count from _chemical_formula_sum:C30 H195 N15 O156 Te2 W34 Yb2
            Atom count from the _atom_site data:  C14 N8 O132.4 Te2 W34 Yb2
CELLZ01_ALERT_1_G Difference between formula and atom_site contents detected.
CELLZ01_ALERT_1_G ALERT: Large difference may be due to a
            symmetry error - see SYMMG tests
           From the CIF: _cell_formula_units_Z    1
           From the CIF: _chemical_formula_sum  C30 H195 N15 O156 Te2 W34 Yb2
           TEST: Compare cell contents of formula and atom_site data

           atom    Z*formula  cif sites diff
           C          30.00     14.00   16.00
           H         195.00      0.00  195.00
           N          15.00      8.00    7.00
           O         156.00    132.40   23.60
           Te          2.00      2.00    0.00
           W          34.00     34.00    0.00
           Yb          2.00      2.00    0.00
PLAT003_ALERT_2_G Number of Uiso or Uij Restrained non-H Atoms ...         20 Report
PLAT012_ALERT_1_G N.O.K.   _shelx_res_checksum Found in CIF ......     Please Check
PLAT040_ALERT_1_G No H-atoms in this Carbon Containing Compound ..     Please Check
PLAT041_ALERT_1_G Calc. and Reported SumFormula    Strings  Differ     Please Check
PLAT066_ALERT_1_G Predicted and Reported Tmin&Tmax Range Identical          ? Check
PLAT068_ALERT_1_G Reported F000 Differs from Calcd (or Missing)...     Please Check
PLAT083_ALERT_2_G SHELXL Second Parameter in WGHT  Unusually Large      84.85 Why ?
PLAT186_ALERT_4_G The CIF-Embedded .res File Contains ISOR Records         11 Report
PLAT300_ALERT_4_G Atom Site Occupancy of C2         Constrained at        0.5 Check
PLAT300_ALERT_4_G Atom Site Occupancy of C7         Constrained at        0.5 Check
PLAT300_ALERT_4_G Atom Site Occupancy of O66        Constrained at        0.7 Check
PLAT300_ALERT_4_G Atom Site Occupancy of O67        Constrained at        0.5 Check
PLAT302_ALERT_4_G Anion/Solvent/Minor-Residue Disorder (Resd  2  )        20% Note
PLAT302_ALERT_4_G Anion/Solvent/Minor-Residue Disorder (Resd  5  )        20% Note
PLAT302_ALERT_4_G Anion/Solvent/Minor-Residue Disorder (Resd  7  )       100% Note
PLAT302_ALERT_4_G Anion/Solvent/Minor-Residue Disorder (Resd  8  )       100% Note
PLAT304_ALERT_4_G Non-Integer Number of Atoms in ..... (Resd  2  )       2.50 Check
PLAT304_ALERT_4_G Non-Integer Number of Atoms in ..... (Resd  5  )       2.50 Check
PLAT304_ALERT_4_G Non-Integer Number of Atoms in ..... (Resd  7  )       0.70 Check
```

```
PLAT304_ALERT_4_G Non-Integer Number of Atoms in ..... (Resd  8  )       0.50 Check
PLAT311_ALERT_2_G Isolated Disordered Oxygen Atom (No H's ?) .....        O66 Check
PLAT311_ALERT_2_G Isolated Disordered Oxygen Atom (No H's ?) .....        O67 Check
PLAT606_ALERT_4_G Solvent Accessible VOID(S) in Structure ........          ! Info
PLAT720_ALERT_4_G Number of Unusual/Non-Standard Labels ..........          1 Note
PLAT790_ALERT_4_G Centre of Gravity not Within Unit Cell: Resd.  #          2 Note
              C1.50 N
PLAT790_ALERT_4_G Centre of Gravity not Within Unit Cell: Resd.  #          4 Note
              C2 N
PLAT790_ALERT_4_G Centre of Gravity not Within Unit Cell: Resd.  #          7 Note
              O
PLAT794_ALERT_5_G Tentative Bond Valency for W1       (VI)      .       6.19 Info
PLAT794_ALERT_5_G Tentative Bond Valency for W2       (VI)      .       6.11 Info
PLAT794_ALERT_5_G Tentative Bond Valency for W3       (VI)      .       6.02 Info
PLAT794_ALERT_5_G Tentative Bond Valency for W4       (VI)      .       6.12 Info
PLAT794_ALERT_5_G Tentative Bond Valency for W5       (VI)      .       6.15 Info
PLAT794_ALERT_5_G Tentative Bond Valency for W6       (VI)      .       6.27 Info
PLAT794_ALERT_5_G Tentative Bond Valency for W7       (VI)      .       6.18 Info
PLAT794_ALERT_5_G Tentative Bond Valency for W8       (VI)      .       6.09 Info
PLAT794_ALERT_5_G Tentative Bond Valency for W9       (VI)      .       6.20 Info
PLAT794_ALERT_5_G Tentative Bond Valency for W10      (VI)      .       6.22 Info
PLAT794_ALERT_5_G Tentative Bond Valency for W11      (VI)      .       6.01 Info
PLAT794_ALERT_5_G Tentative Bond Valency for W12      (VI)      .       6.22 Info
PLAT794_ALERT_5_G Tentative Bond Valency for W13      (VI)      .       6.30 Info
PLAT794_ALERT_5_G Tentative Bond Valency for W14      (VI)      .       6.15 Info
PLAT794_ALERT_5_G Tentative Bond Valency for W15      (VI)      .       6.05 Info
PLAT794_ALERT_5_G Tentative Bond Valency for W16      (VI)      .       6.07 Info
PLAT794_ALERT_5_G Tentative Bond Valency for W17      (VI)      .       5.96 Info
PLAT794_ALERT_5_G Tentative Bond Valency for Yb1      (III)     .       3.09 Info
PLAT794_ALERT_5_G Tentative Bond Valency for Te1      (VI)      .       5.41 Info
PLAT860_ALERT_3_G Number of Least-Squares Restraints .............        120 Note
PLAT868_ALERT_4_G ALERTS Due to the Use of _smtbx_masks Suppressed          ! Info
PLAT933_ALERT_2_G Number of OMIT Records in Embedded .res File ...          1 Note
PLAT941_ALERT_3_G Average HKL Measurement Multiplicity ...........        2.1 Low
```

---

0 **ALERT level A** = Most likely a serious problem - resolve or explain
1 **ALERT level B** = A potentially serious problem, consider carefully
2 **ALERT level C** = Check. Ensure it is not caused by an omission or oversight
53 **ALERT level G** = General information/check it is not something unexpected

7 ALERT type 1 CIF construction/syntax error, inconsistent or missing data
8 ALERT type 2 Indicator that the structure model may be wrong or deficient
2 ALERT type 3 Indicator that the structure quality may be low
20 ALERT type 4 Improvement, methodology, query or suggestion
19 ALERT type 5 Informative message, check

Datablock: compound_dimer_Lu

Bond precision: N- C = 0.0205 A   Wavelength=0.71073

Cell:	a=14.0315(3)	b=14.4627(3)	c=23.4500(4)
	alpha=106.240(2)	beta=94.616(2)	gamma=110.580(2)
Temperature:	296 K		

	Calculated	Reported
Volume	4191.86(17)	4191.86(16)
Space group	P -1	P -1
Hall group	-P 1	-P 1
Moiety formula	Lu2 O128 Te2 W34, 8(C2 N), 5(O) [+ solvent]	?
Sum formula	C16 Lu2 N8 O133 Te2 W34 [+ solvent]	C30 H199 Lu2 N15 O158 Te2 W34
Mr	9287.94	10154.60
Dx,g cm-3	3.679	4.022
Z	1	1
Mu (mm-1)	24.814	24.814
F000	3978.0	3978.0
F000’	3954.67	
h,k,lmax	20,21,34	20,20,34
Nref	27973	24701
Tmin,Tmax	0.004,0.084	0.004,0.084
Tmin’	0.000	

Correction method= # Reported T Limits: Tmin=0.004 Tmax=0.084
AbsCorr = MULTI-SCAN

Data completeness= 0.883 Theta(max)= 31.523

R(reflections)= 0.0440(18390) wR2(reflections)= 0.1013(24701)

S = 1.026 Npar= 883

The following ALERTS were generated. Each ALERT has the format
test-name_ALERT_alert-type_alert-level.
Click on the hyperlinks for more details of the test.

[IMAGE] **Alert level B**

```
PLAT306_ALERT_2_B Isolated Oxygen Atom (H-atoms Missing ?) .......        O65 Check
PLAT306_ALERT_2_B Isolated Oxygen Atom (H-atoms Missing ?) .......        O66 Check
```

[IMAGE] **Alert level C**

```
PLAT213_ALERT_2_C Atom O33             has ADP max/min Ratio .....        3.3 oblate
PLAT244_ALERT_4_C Low    'Solvent' Ueq as Compared to Neighbors of         N2 Check
PLAT244_ALERT_4_C Low    'Solvent' Ueq as Compared to Neighbors of         N4 Check
```

[IMAGE] **Alert level G**

```
FORMU01_ALERT_2_G  There is a discrepancy between the atom counts in the
            _chemical_formula_sum and the formula from the _atom_site* data.
            Atom count from _chemical_formula_sum:C30 H199 Lu2 N15 O158 Te2 W34
            Atom count from the _atom_site data:  C16 Lu2 N8 O133 Te2 W34
CELLZ01_ALERT_1_G Difference between formula and atom_site contents detected.
CELLZ01_ALERT_1_G ALERT: Large difference may be due to a
            symmetry error - see SYMMG tests
           From the CIF: _cell_formula_units_Z    1
```

```
From the CIF: _chemical_formula_sum  C30 H199 Lu2 N15 O158 Te2 W34
TEST: Compare cell contents of formula and atom_site data

atom    Z*formula  cif sites diff
C         30.00     16.00   14.00
H        199.00      0.00  199.00
Lu         2.00      2.00    0.00
N         15.00      8.00    7.00
O        158.00    133.00   25.00
Te         2.00      2.00    0.00
W         34.00     34.00    0.00
```

```
PLAT003_ALERT_2_G Number of Uiso or Uij Restrained non-H Atoms ...          6 Report
PLAT012_ALERT_1_G N.O.K.   _shelx_res_checksum Found in CIF ......     Please Check
PLAT040_ALERT_1_G No H-atoms in this Carbon Containing Compound ..     Please Check
PLAT041_ALERT_1_G Calc. and Reported SumFormula    Strings  Differ     Please Check
PLAT066_ALERT_1_G Predicted and Reported Tmin&Tmax Range Identical          ? Check
PLAT154_ALERT_1_G The s.u.'s on the Cell Angles are Equal ..(Note)      0.002 Degree
PLAT180_ALERT_4_G Check Cell Rounding: # of Values Ending with 0 =          3 Note
PLAT186_ALERT_4_G The CIF-Embedded .res File Contains ISOR Records          6 Report
PLAT300_ALERT_4_G Atom Site Occupancy of O67        Constrained at        0.5 Check
PLAT302_ALERT_4_G Anion/Solvent/Minor-Residue Disorder (Resd  8  )       100% Note
PLAT304_ALERT_4_G Non-Integer Number of Atoms in ..... (Resd  8  )       0.50 Check
PLAT311_ALERT_2_G Isolated Disordered Oxygen Atom (No H's ?) .....        O67 Check
PLAT606_ALERT_4_G Solvent Accessible VOID(S) in Structure ........          ! Info
PLAT794_ALERT_5_G Tentative Bond Valency for W1       (VI)      .       6.08 Info
PLAT794_ALERT_5_G Tentative Bond Valency for W2       (VI)      .       5.97 Info
PLAT794_ALERT_5_G Tentative Bond Valency for W3       (VI)      .       6.08 Info
PLAT794_ALERT_5_G Tentative Bond Valency for W4       (VI)      .       6.20 Info
PLAT794_ALERT_5_G Tentative Bond Valency for W5       (VI)      .       6.14 Info
PLAT794_ALERT_5_G Tentative Bond Valency for W6       (VI)      .       6.06 Info
PLAT794_ALERT_5_G Tentative Bond Valency for W7       (VI)      .       6.16 Info
PLAT794_ALERT_5_G Tentative Bond Valency for W8       (VI)      .       6.09 Info
PLAT794_ALERT_5_G Tentative Bond Valency for W9       (VI)      .       6.27 Info
PLAT794_ALERT_5_G Tentative Bond Valency for W10      (VI)      .       6.16 Info
PLAT794_ALERT_5_G Tentative Bond Valency for W11      (VI)      .       6.23 Info
PLAT794_ALERT_5_G Tentative Bond Valency for W12      (VI)      .       6.14 Info
PLAT794_ALERT_5_G Tentative Bond Valency for W13      (VI)      .       6.18 Info
PLAT794_ALERT_5_G Tentative Bond Valency for W14      (VI)      .       6.14 Info
PLAT794_ALERT_5_G Tentative Bond Valency for W15      (VI)      .       6.10 Info
PLAT794_ALERT_5_G Tentative Bond Valency for W16      (VI)      .       6.19 Info
PLAT794_ALERT_5_G Tentative Bond Valency for W17      (VI)      .       6.09 Info
PLAT794_ALERT_5_G Tentative Bond Valency for Lu1      (III)     .       3.20 Info
PLAT794_ALERT_5_G Tentative Bond Valency for Te1      (VI)      .       5.48 Info
PLAT860_ALERT_3_G Number of Least-Squares Restraints .............         36 Note
PLAT868_ALERT_4_G ALERTS Due to the Use of _smtbx_masks Suppressed          ! Info
PLAT933_ALERT_2_G Number of OMIT Records in Embedded .res File ...         12 Note
PLAT941_ALERT_3_G Average HKL Measurement Multiplicity ..........         2.2 Low
```

---

0 **ALERT level A** = Most likely a serious problem - resolve or explain
2 **ALERT level B** = A potentially serious problem, consider carefully
3 **ALERT level C** = Check. Ensure it is not caused by an omission or oversight
39 **ALERT level G** = General information/check it is not something unexpected

7 ALERT type 1 CIF construction/syntax error, inconsistent or missing data
7 ALERT type 2 Indicator that the structure model may be wrong or deficient
2 ALERT type 3 Indicator that the structure quality may be low
9 ALERT type 4 Improvement, methodology, query or suggestion
19 ALERT type 5 Informative message, check

It is advisable to attempt to resolve as many as possible of the alerts in all categories. Often the minor alerts point to easily fixed oversights, errors and omissions in your CIF or refinement strategy, so attention to these fine details can be worthwhile. In order to resolve some of the more serious problems it may be necessary to carry out additional measurements or structure refinements. However, the purpose of your study may justify the reported deviations and the more serious of these should normally be commented upon in the discussion or experimental section of a paper or in the "special_details" fields of the CIF. checkCIF was carefully designed to identify outliers and unusual parameters, but every test has its limitations and alerts that are not important in a particular case may appear. Conversely, the absence of alerts does not guarantee there are no aspects of the results needing attention. It is up to the individual to critically assess their own results and, if necessary, seek expert advice.

Publication of your CIF in IUCr journals

A basic structural check has been run on your CIF. These basic checks will be run on all CIFs submitted for publication in IUCr journals (*Acta Crystallographica*, *Journal of Applied Crystallography*, *Journal of Synchrotron Radiation*); however, if you intend to submit to *Acta Crystallographica Section C* or *E* or *IUCrData*, you should make sure that full publication checks are run on the final version of your CIF prior to submission.

Publication of your CIF in other journals

Please refer to the *Notes for Authors* of the relevant journal for any special instructions relating to CIF submission.

PLATON version of 10/08/2020; check.def file version of 06/08/2020

Datablock compound_dimer_Dy - ellipsoid plot

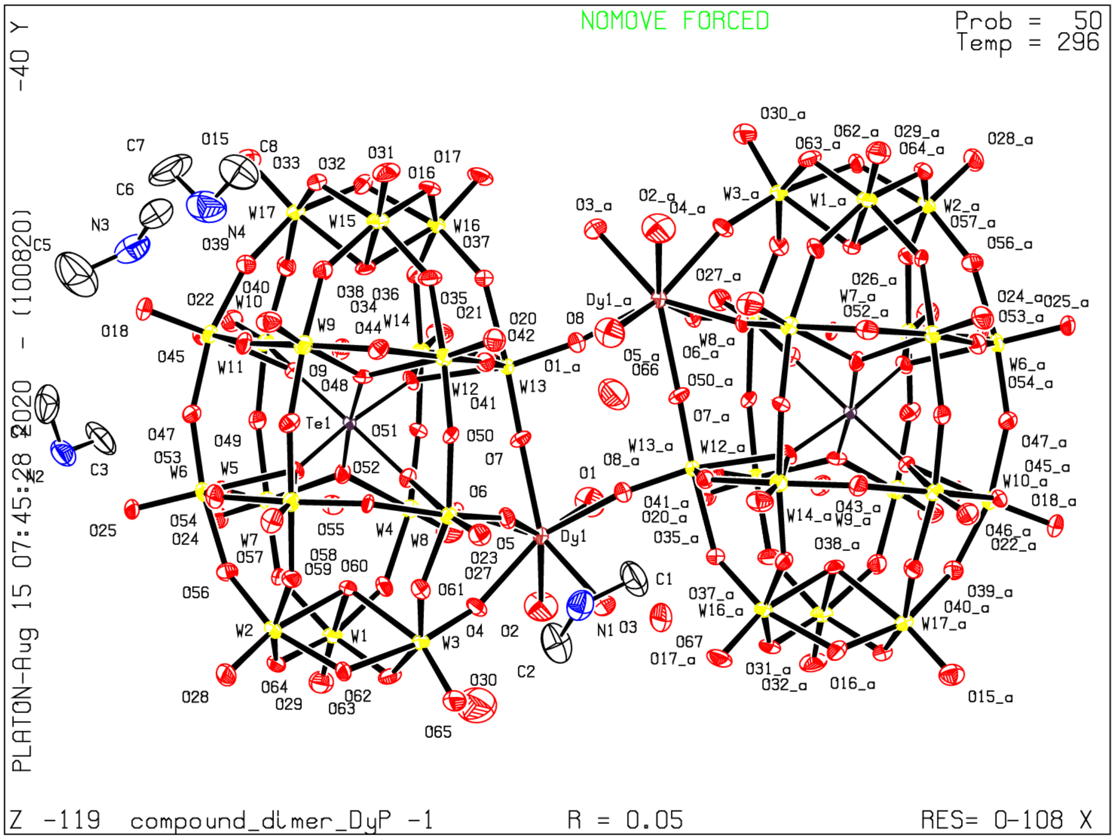

Datablock compound_dimer_Ho - ellipsoid plot

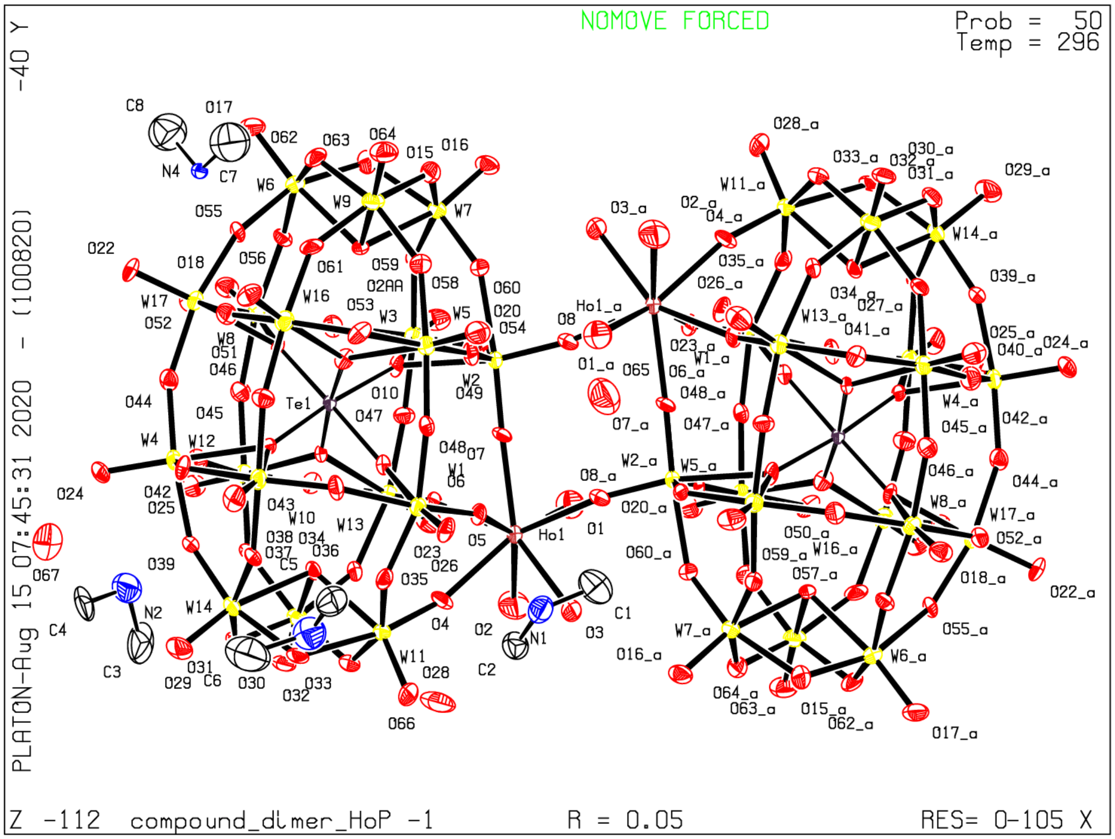

Datablock compound_dimer_Er - ellipsoid plot

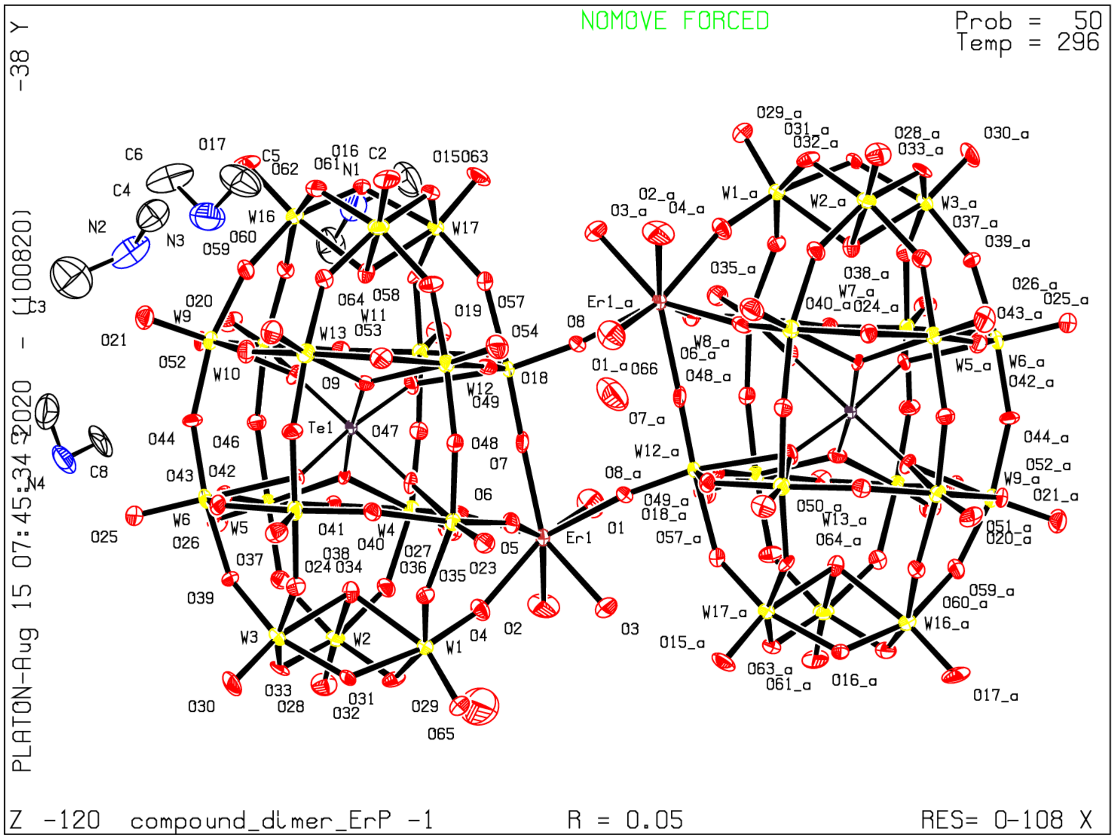

Datablock compound_dimer_Tm - ellipsoid plot

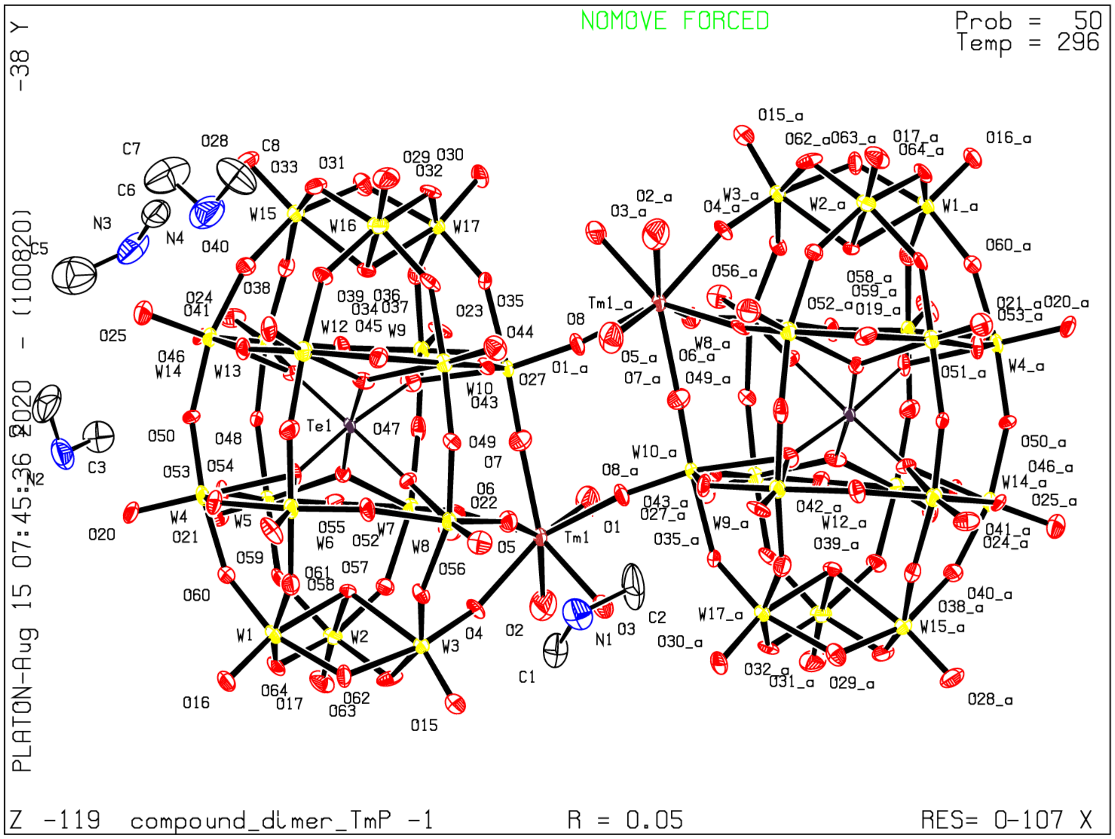

Datablock compound_dimer_Yb - ellipsoid plot

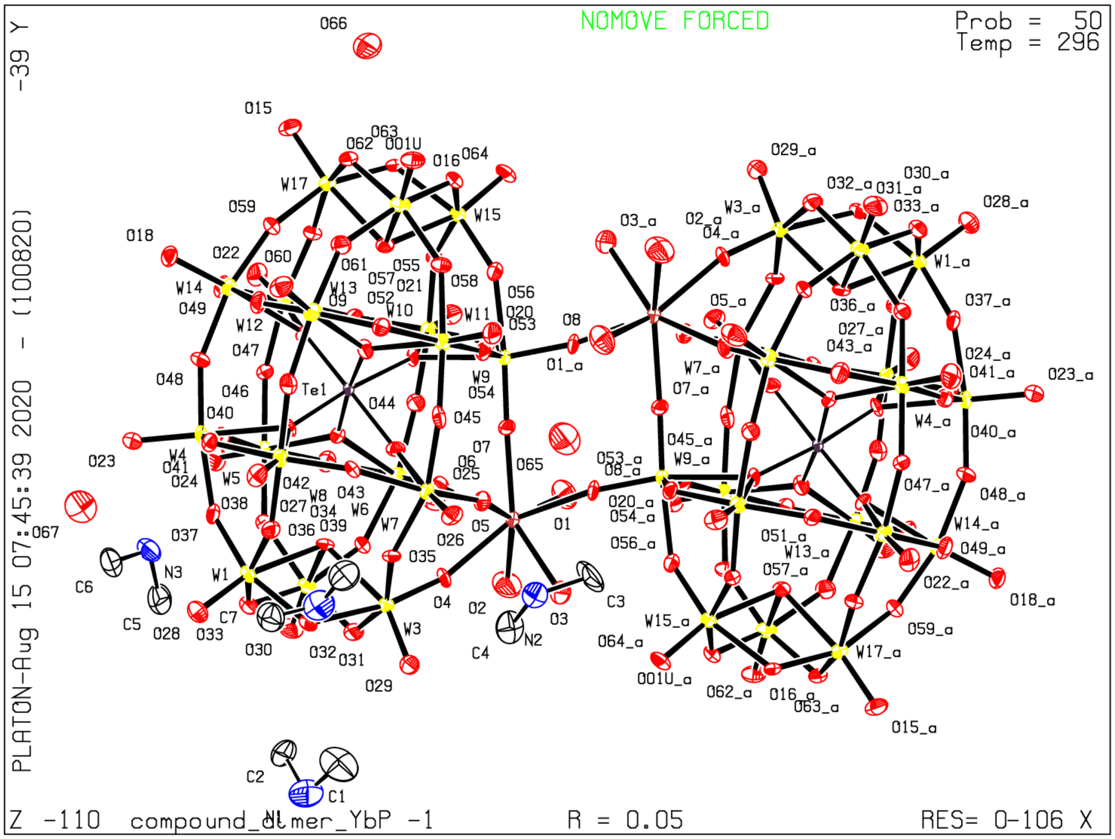

Datablock compound_dimer_Lu - ellipsoid plot

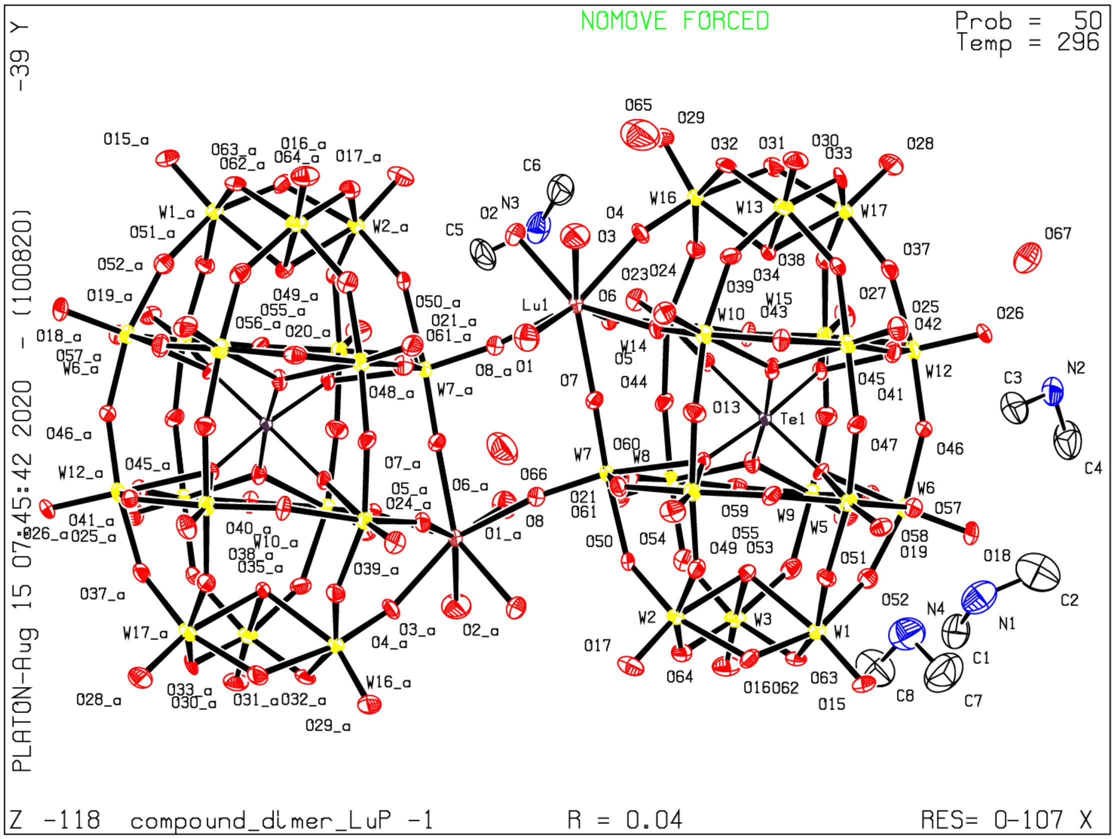

Supplement: Supplementary file 2 [file Data_Sheet_1.PDF]
