# Supplementary material for: Assembly of Lanthanide-Containing Tungstotellurates(VI): Syntheses, Structures, and Catalytic Properties
Source: Front Chem. 2020 Nov 23;8:598961. doi: 10.3389/fchem.2020.598961 (PMC7719746; doi:10.3389/fchem.2020.598961)

checkCIF/PLATON report

You have not supplied any structure factors. As a result the full set of tests cannot be run.

THIS REPORT IS FOR GUIDANCE ONLY. IF USED AS PART OF A REVIEW PROCEDURE FOR PUBLICATION, IT SHOULD NOT REPLACE THE EXPERTISE OF AN EXPERIENCED CRYSTALLOGRAPHIC REFEREE.

No syntax errors found.   CIF dictionary   Interpreting this report

Datablock: compound_monosubstituted_Dy

```
Bond precision:  N- C = 0.0150 A         Wavelength=0.71073

Cell:          a=13.1380(2)       b=14.8767(3)     c=24.9758(4)
               alpha=102.256(2)   beta=96.206(1)   gamma=90.528(1)
Temperature:   296 K
```

	Calculated	Reported
Volume	4739.71(15)	4739.71(15)
Space group	P -1	P -1
Hall group	-P 1	-P 1
Moiety formula	Dy O65 Te W17, C2 N [+ solvent]	?
Sum formula	C2 Dy N O65 Te W17 [+ solvent]	C14 H112 Dy N7 O88 Te W17 Na2
Mr	4493.41	5248.39
Dx,g cm-3	3.148	3.677
Z	2	2
Mu (mm-1)	21.686	21.686
F000	3830.0	3830.0
F000’	3807.30	
h,k,lmax	19,21,36	19,21,36
Nref	31970	27973
Tmin,Tmax	0.008,0.114	0.008,0.114
Tmin’	0.001	

```
Correction method= # Reported T Limits: Tmin=0.008 Tmax=0.114
AbsCorr = MULTI-SCAN

Data completeness= 0.875          Theta(max)= 31.656

R(reflections)= 0.0428( 20093)    wR2(reflections)= 0.0987( 27973)

S = 1.036                 Npar= 784
```

The following ALERTS were generated. Each ALERT has the format
test-name_ALERT_alert-type_alert-level.
Click on the hyperlinks for more details of the test.

[IMAGE] Alert level B

PLAT097_ALERT_2_B Large Reported Max. (Positive) Residual Density 7.67 eA-3

[IMAGE] Alert level C

DIFMX02_ALERT_1_C The maximum difference density is > 0.1*ZMAX*0.75
The relevant atom site should be identified.
PLAT094_ALERT_2_C Ratio of Maximum / Minimum Residual Density .... 3.14 Report
PLAT220_ALERT_2_C NonSolvent Resd 1 O Ueq(max)/Ueq(min) Range 3.1 Ratio
PLAT242_ALERT_2_C Low 'MainMol' Ueq as Compared to Neighbors of Dy1 Check

---

[IMAGE] Alert level G

FORMU01_ALERT_2_G There is a discrepancy between the atom counts in the
_chemical_formula_sum and the formula from the _atom_site* data.
Atom count from _chemical_formula_sum:C14 H112 Dy1 N7 Na2 O88 Te1 W17
Atom count from the _atom_site data: C2 Dy1 N1 O65 Te1 W17
CELLZ01_ALERT_1_G Difference between formula and atom_site contents detected.
CELLZ01_ALERT_1_G ALERT: Large difference may be due to a
symmetry error - see SYMMG tests
From the CIF: _cell_formula_units_Z 2
From the CIF: _chemical_formula_sum C14 H112 Dy N7 O88 Te W17 Na2
TEST: Compare cell contents of formula and atom_site data

atom	Z*formula	cif sites	diff
C	28.00	4.00	24.00
H	224.00	0.00	224.00
Dy	2.00	2.00	0.00
N	14.00	2.00	12.00
O	176.00	130.00	46.00
Te	2.00	2.00	0.00
W	34.00	34.00	0.00
Na	4.00	0.00	4.00

PLAT012_ALERT_1_G N.O.K. _shelx_res_checksum Found in CIF ...... Please Check
PLAT040_ALERT_1_G No H-atoms in this Carbon Containing Compound .. Please Check
PLAT041_ALERT_1_G Calc. and Reported SumFormula Strings Differ Please Check
PLAT066_ALERT_1_G Predicted and Reported Tmin&Tmax Range Identical ? Check
PLAT606_ALERT_4_G Solvent Accessible VOID(S) in Structure .... ! Info
PLAT794_ALERT_5_G Tentative Bond Valency for W1 (VI) . 5.98 Info
PLAT794_ALERT_5_G Tentative Bond Valency for W2 (VI) . 6.21 Info
PLAT794_ALERT_5_G Tentative Bond Valency for W3 (VI) . 6.14 Info
PLAT794_ALERT_5_G Tentative Bond Valency for W4 (VI) . 6.16 Info
PLAT794_ALERT_5_G Tentative Bond Valency for W5 (VI) . 6.17 Info
PLAT794_ALERT_5_G Tentative Bond Valency for W6 (VI) . 6.17 Info
PLAT794_ALERT_5_G Tentative Bond Valency for W7 (VI) . 6.22 Info
PLAT794_ALERT_5_G Tentative Bond Valency for W8 (VI) . 6.26 Info
PLAT794_ALERT_5_G Tentative Bond Valency for W9 (VI) . 6.09 Info
PLAT794_ALERT_5_G Tentative Bond Valency for W10 (VI) . 6.24 Info
PLAT794_ALERT_5_G Tentative Bond Valency for W11 (VI) . 6.20 Info
PLAT794_ALERT_5_G Tentative Bond Valency for W12 (VI) . 6.14 Info
PLAT794_ALERT_5_G Tentative Bond Valency for W13 (VI) . 6.24 Info
PLAT794_ALERT_5_G Tentative Bond Valency for W14 (VI) . 6.21 Info
PLAT794_ALERT_5_G Tentative Bond Valency for W15 (VI) . 6.21 Info
PLAT794_ALERT_5_G Tentative Bond Valency for W16 (VI) . 6.19 Info
PLAT794_ALERT_5_G Tentative Bond Valency for W17 (VI) . 6.29 Info
PLAT794_ALERT_5_G Tentative Bond Valency for Dy1 (III) . 3.07 Info
PLAT794_ALERT_5_G Tentative Bond Valency for Te1 (VI) . 5.39 Info

```
PLAT868_ALERT_4_G ALERTS Due to the Use of _smtbx_masks Suppressed          ! Info
PLAT933_ALERT_2_G Number of OMIT Records in Embedded .res File ...          30 Note
PLAT941_ALERT_3_G Average HKL Measurement Multiplicity ...........         2.3 Low
```

```
   0 ALERT level A = Most likely a serious problem - resolve or explain
   1 ALERT level B = A potentially serious problem, consider carefully
   4 ALERT level C = Check. Ensure it is not caused by an omission or oversight
  30 ALERT level G = General information/check it is not something unexpected

   7 ALERT type 1 CIF construction/syntax error, inconsistent or missing data
   6 ALERT type 2 Indicator that the structure model may be wrong or deficient
   1 ALERT type 3 Indicator that the structure quality may be low
   2 ALERT type 4 Improvement, methodology, query or suggestion
  19 ALERT type 5 Informative message, check
```

Datablock: compound_monosubstituted-Ho

Bond precision: N- C = 0.0197 A   Wavelength=0.71073

Cell: a=13.1394(2) b=14.8740(3) c=24.9631(4)
alpha=102.246(2) beta=96.183(2) gamma=90.589(2)
Temperature: 296 K

	Calculated	Reported
Volume	4737.18(15)	4737.17(15)
Space group	P -1	P -1
Hall group	-P 1	-P 1
Moiety formula	2(Ho O65 Te W17), 4(C2 N), 5(O) [+ solvent]	?
Sum formula	C8 Ho2 N4 O135 Te2 W34 [+ solvent]	C14 H120 Ho N7 O92 Te W17 Na2
Mr	9147.74	5322.88
Dx,g cm-3	3.207	3.731
Z	1	2
Mu (mm-1)	21.749	21.749
F000	3910.0	3910.0
F000’	3887.17	
h,k,lmax	19,21,36	19,21,36
Nref	31739	27751
Tmin,Tmax	0.008,0.114	0.008,0.114
Tmin’	0.001	

Correction method= # Reported T Limits: Tmin=0.008 Tmax=0.114
AbsCorr = MULTI-SCAN

Data completeness= 0.874   Theta(max)= 31.576

R(reflections)= 0.0474(20251)   wR2(reflections)= 0.1152(27751)

S = 1.083 Npar= 838

The following ALERTS were generated. Each ALERT has the format
test-name_ALERT_alert-type_alert-level.
Click on the hyperlinks for more details of the test.

[IMAGE] Alert level B

```
PLAT306_ALERT_2_B Isolated Oxygen Atom (H-atoms Missing ?) .......        O66 Check
PLAT306_ALERT_2_B Isolated Oxygen Atom (H-atoms Missing ?) .......        O67 Check
```

[IMAGE] Alert level C

```
DIFMX02_ALERT_1_C  The maximum difference density is > 0.1*ZMAX*0.75
            The relevant atom site should be identified.
PLAT094_ALERT_2_C Ratio of Maximum / Minimum Residual Density ....       2.26 Report
PLAT097_ALERT_2_C Large Reported Max.  (Positive) Residual Density       6.30 eA-3
PLAT220_ALERT_2_C NonSolvent   Resd 1  O   Ueq(max)/Ueq(min) Range        3.2 Ratio
PLAT242_ALERT_2_C Low    'MainMol' Ueq as Compared to Neighbors of        Ho1 Check
PLAT244_ALERT_4_C Low    'Solvent' Ueq as Compared to Neighbors of         N1 Check
PLAT260_ALERT_2_C Large Average Ueq of Residue Including       O67      0.113 Check
```

[IMAGE] Alert level G

```
FORMU01_ALERT_2_G  There is a discrepancy between the atom counts in the
            _chemical_formula_sum and the formula from the _atom_site* data.
            Atom count from _chemical_formula_sum:C14 H120 Ho1 N7 Na2 O92 Te1 W17
            Atom count from the _atom_site data:  C4 Ho1 N2 O67.5 Te1 W17
CELLZ01_ALERT_1_G Difference between formula and atom_site contents detected.
CELLZ01_ALERT_1_G ALERT: Large difference may be due to a
            symmetry error - see SYMMG tests
           From the CIF: _cell_formula_units_Z    2
           From the CIF: _chemical_formula_sum  C14 H120 Ho N7 O92 Te W17 Na2
           TEST: Compare cell contents of formula and atom_site data
```

atom	Z*formula	cif sites	diff
C	28.00	8.00	20.00
H	240.00	0.00	240.00
Ho	2.00	2.00	0.00
N	14.00	4.00	10.00
O	184.00	135.00	49.00
Te	2.00	2.00	0.00
W	34.00	34.00	0.00
Na	4.00	0.00	4.00

```
PLAT003_ALERT_2_G Number of Uiso or Uij Restrained non-H Atoms ...          4 Report
PLAT012_ALERT_1_G N.O.K.   _shelx_res_checksum Found in CIF ......     Please Check
PLAT040_ALERT_1_G No H-atoms in this Carbon Containing Compound ..     Please Check
PLAT041_ALERT_1_G Calc. and Reported SumFormula    Strings  Differ     Please Check
PLAT045_ALERT_1_G Calculated and Reported Z Differ by a Factor ...       0.50 Check
PLAT066_ALERT_1_G Predicted and Reported Tmin&Tmax Range Identical          ? Check
PLAT083_ALERT_2_G SHELXL Second Parameter in WGHT  Unusually Large      23.95 Why ?
PLAT154_ALERT_1_G The s.u.'s on the Cell Angles are Equal ..(Note)      0.002 Degree
PLAT186_ALERT_4_G The CIF-Embedded .res File Contains ISOR Records          3 Report
PLAT300_ALERT_4_G Atom Site Occupancy of O68       Constrained at         0.5 Check
PLAT302_ALERT_4_G Anion/Solvent/Minor-Residue Disorder (Resd  6  )       100% Note
PLAT304_ALERT_4_G Non-Integer Number of Atoms in ..... (Resd  6  )       0.50 Check
PLAT311_ALERT_2_G Isolated Disordered Oxygen Atom (No H's ?) .....        O68 Check
PLAT606_ALERT_4_G Solvent Accessible VOID(S) in Structure ........          ! Info
PLAT794_ALERT_5_G Tentative Bond Valency for W1        (VI)      .       6.30 Info
PLAT794_ALERT_5_G Tentative Bond Valency for W2        (VI)      .       6.23 Info
```

```
PLAT794_ALERT_5_G Tentative Bond Valency for W3        (VI)      .       6.13 Info
PLAT794_ALERT_5_G Tentative Bond Valency for W4        (VI)      .       6.18 Info
PLAT794_ALERT_5_G Tentative Bond Valency for W5        (VI)      .       6.21 Info
PLAT794_ALERT_5_G Tentative Bond Valency for W6        (VI)      .       6.21 Info
PLAT794_ALERT_5_G Tentative Bond Valency for W7        (VI)      .       6.24 Info
PLAT794_ALERT_5_G Tentative Bond Valency for W8        (VI)      .       6.40 Info
PLAT794_ALERT_5_G Tentative Bond Valency for W9        (VI)      .       6.15 Info
PLAT794_ALERT_5_G Tentative Bond Valency for W10       (VI)      .       6.23 Info
PLAT794_ALERT_5_G Tentative Bond Valency for W11       (VI)      .       6.20 Info
PLAT794_ALERT_5_G Tentative Bond Valency for W12       (VI)      .       6.17 Info
PLAT794_ALERT_5_G Tentative Bond Valency for W13       (VI)      .       6.17 Info
PLAT794_ALERT_5_G Tentative Bond Valency for W14       (VI)      .       6.15 Info
PLAT794_ALERT_5_G Tentative Bond Valency for W15       (VI)      .       6.09 Info
PLAT794_ALERT_5_G Tentative Bond Valency for W16       (VI)      .       6.15 Info
PLAT794_ALERT_5_G Tentative Bond Valency for W17       (VI)      .       6.22 Info
PLAT794_ALERT_5_G Tentative Bond Valency for Ho1       (III)     .       3.32 Info
PLAT794_ALERT_5_G Tentative Bond Valency for Te1       (VI)      .       5.25 Info
PLAT860_ALERT_3_G Number of Least-Squares Restraints .............         24 Note
PLAT868_ALERT_4_G ALERTS Due to the Use of _smtbx_masks Suppressed          ! Info
PLAT933_ALERT_2_G Number of OMIT Records in Embedded .res File ...          3 Note
PLAT941_ALERT_3_G Average HKL Measurement Multiplicity ...........        2.2 Low
```

```
   0 ALERT level A = Most likely a serious problem - resolve or explain
   2 ALERT level B = A potentially serious problem, consider carefully
   7 ALERT level C = Check. Ensure it is not caused by an omission or oversight
  40 ALERT level G = General information/check it is not something unexpected

   9 ALERT type 1 CIF construction/syntax error, inconsistent or missing data
  12 ALERT type 2 Indicator that the structure model may be wrong or deficient
   2 ALERT type 3 Indicator that the structure quality may be low
   7 ALERT type 4 Improvement, methodology, query or suggestion
  19 ALERT type 5 Informative message, check
```

Datablock: compound_monosubstituted_Er

```
Bond precision:  N- C = 0.0300 A         Wavelength=0.71073

Cell:            a=13.1527(3)        b=14.9285(4)     c=24.9988(6)
                 alpha=102.295(2)    beta=96.046(2)   gamma=90.808(2)
Temperature:     296 K
```

	Calculated	Reported
Volume	4765.9(2)	4765.9(2)
Space group	P -1	P -1
Hall group	-P 1	-P 1
Moiety formula	Er O65 Te W17, 2(C2 N), 1.75(O) [+ solvent]	?
Sum formula	C4 Er N2 O66.75 Te W17 [+ solvent]	C14 H110 Na2 Er N7 O87 Te W17
Mr	4564.20	5235.14
Dx,g cm-3	3.181	3.648
Z	2	2
Mu (mm-1)	21.667	21.667
F000	3900.0	3900.0
F000’	3877.13	
h,k,lmax	19,21,36	19,21,36
Nref	31728	27774
Tmin,Tmax	0.008,0.115	0.008,0.115
Tmin’	0.001	

Correction method= # Reported T Limits: Tmin=0.008 Tmax=0.115
AbsCorr = MULTI-SCAN

Data completeness= 0.875 Theta(max)= 31.500

R(reflections)= 0.0641(19476) wR2(reflections)= 0.1624(27774)

S = 1.062 Npar= 829

The following ALERTS were generated. Each ALERT has the format
test-name_ALERT_alert-type_alert-level.
Click on the hyperlinks for more details of the test.

[IMAGE] **Alert level B**

```
PLAT097_ALERT_2_B Large Reported Max.  (Positive) Residual Density      14.35 eA-3
PLAT306_ALERT_2_B Isolated Oxygen Atom (H-atoms Missing ?) .......        O67 Check
```

[IMAGE] **Alert level C**

```
DIFMX02_ALERT_1_C  The maximum difference density is > 0.1*ZMAX*0.75
            The relevant atom site should be identified.
PLAT094_ALERT_2_C Ratio of Maximum / Minimum Residual Density ....       3.20 Report
PLAT213_ALERT_2_C Atom O3              has ADP max/min Ratio .....        3.1 oblate
PLAT213_ALERT_2_C Atom O29             has ADP max/min Ratio .....        3.4 prolat
PLAT220_ALERT_2_C NonSolvent   Resd 1  O   Ueq(max)/Ueq(min) Range        4.4 Ratio
PLAT242_ALERT_2_C Low    'MainMol' Ueq as Compared to Neighbors of        Er1 Check
PLAT244_ALERT_4_C Low    'Solvent' Ueq as Compared to Neighbors of         N1 Check
```

[IMAGE] **Alert level G**

```
FORMU01_ALERT_2_G  There is a discrepancy between the atom counts in the
            _chemical_formula_sum and the formula from the _atom_site* data.
            Atom count from _chemical_formula_sum:C14 H110 Er1 N7 Na2 O87 Te1 W17
```

```
            Atom count from the _atom_site data:  C4 Er1 N2 O66.75 Te1 W17
CELLZ01_ALERT_1_G Difference between formula and atom_site contents detected.
CELLZ01_ALERT_1_G ALERT: Large difference may be due to a
            symmetry error - see SYMMG tests
           From the CIF: _cell_formula_units_Z    2
           From the CIF: _chemical_formula_sum  C14 H110 Na2 Er N7 O87 Te W17
           TEST: Compare cell contents of formula and atom_site data

           atom    Z*formula  cif sites diff
           C         28.00      8.00   20.00
           H        220.00      0.00  220.00
           Na         4.00      0.00    4.00
           Er         2.00      2.00    0.00
           N         14.00      4.00   10.00
           O        174.00    133.50   40.50
           Te         2.00      2.00    0.00
           W         34.00     34.00    0.00
PLAT003_ALERT_2_G Number of Uiso or Uij Restrained non-H Atoms ...          4 Report
PLAT012_ALERT_1_G N.O.K.   _shelx_res_checksum Found in CIF ......     Please Check
PLAT040_ALERT_1_G No H-atoms in this Carbon Containing Compound ..     Please Check
PLAT041_ALERT_1_G Calc. and Reported SumFormula    Strings  Differ     Please Check
PLAT066_ALERT_1_G Predicted and Reported Tmin&Tmax Range Identical          ? Check
PLAT083_ALERT_2_G SHELXL Second Parameter in WGHT  Unusually Large     119.04 Why ?
PLAT154_ALERT_1_G The s.u.'s on the Cell Angles are Equal ..(Note)      0.002 Degree
PLAT186_ALERT_4_G The CIF-Embedded .res File Contains ISOR Records          3 Report
PLAT300_ALERT_4_G Atom Site Occupancy of O66       Constrained at       0.75 Check
PLAT302_ALERT_4_G Anion/Solvent/Minor-Residue Disorder (Resd  4  )       100% Note
PLAT304_ALERT_4_G Non-Integer Number of Atoms in ..... (Resd  4  )       0.75 Check
PLAT311_ALERT_2_G Isolated Disordered Oxygen Atom (No H's ?) .....        O66 Check
PLAT606_ALERT_4_G Solvent Accessible VOID(S) in Structure ........          ! Info
PLAT794_ALERT_5_G Tentative Bond Valency for W1       (VI)      .       6.09 Info
PLAT794_ALERT_5_G Tentative Bond Valency for W2       (VI)      .       6.11 Info
PLAT794_ALERT_5_G Tentative Bond Valency for W3       (VI)      .       6.12 Info
PLAT794_ALERT_5_G Tentative Bond Valency for W4       (VI)      .       6.06 Info
PLAT794_ALERT_5_G Tentative Bond Valency for W5       (VI)      .       6.12 Info
PLAT794_ALERT_5_G Tentative Bond Valency for W6       (VI)      .       6.06 Info
PLAT794_ALERT_5_G Tentative Bond Valency for W7       (VI)      .       6.29 Info
PLAT794_ALERT_5_G Tentative Bond Valency for W8       (VI)      .       6.15 Info
PLAT794_ALERT_5_G Tentative Bond Valency for W9       (VI)      .       6.26 Info
PLAT794_ALERT_5_G Tentative Bond Valency for W10      (VI)      .       6.19 Info
PLAT794_ALERT_5_G Tentative Bond Valency for W11      (VI)      .       6.12 Info
PLAT794_ALERT_5_G Tentative Bond Valency for W12      (VI)      .       6.14 Info
PLAT794_ALERT_5_G Tentative Bond Valency for W13      (VI)      .       6.06 Info
PLAT794_ALERT_5_G Tentative Bond Valency for W14      (VI)      .       6.21 Info
PLAT794_ALERT_5_G Tentative Bond Valency for W15      (VI)      .       6.20 Info
PLAT794_ALERT_5_G Tentative Bond Valency for W16      (VI)      .       6.31 Info
PLAT794_ALERT_5_G Tentative Bond Valency for W17      (VI)      .       6.04 Info
PLAT794_ALERT_5_G Tentative Bond Valency for Er1      (III)     .       3.04 Info
PLAT794_ALERT_5_G Tentative Bond Valency for Te1      (VI)      .       5.09 Info
PLAT860_ALERT_3_G Number of Least-Squares Restraints .............         24 Note
PLAT868_ALERT_4_G ALERTS Due to the Use of _smtbx_masks Suppressed          ! Info
PLAT933_ALERT_2_G Number of OMIT Records in Embedded .res File ...          3 Note
PLAT941_ALERT_3_G Average HKL Measurement Multiplicity ...........        2.2 Low
```

---

0 **ALERT level A** = Most likely a serious problem - resolve or explain
2 **ALERT level B** = A potentially serious problem, consider carefully
7 **ALERT level C** = Check. Ensure it is not caused by an omission or oversight
39 **ALERT level G** = General information/check it is not something unexpected

8 ALERT type 1 CIF construction/syntax error, inconsistent or missing data

12 ALERT type 2 Indicator that the structure model may be wrong or deficient
2 ALERT type 3 Indicator that the structure quality may be low
7 ALERT type 4 Improvement, methodology, query or suggestion
19 ALERT type 5 Informative message, check

Datablock: compound_monosubstituted_Tm

Bond precision: N- C = 0.0192 A Wavelength=0.71073

Cell:	a=13.1395(4)	b=14.8770(4)	c=24.9813(5)
	alpha=102.428(2)	beta=96.280(2)	gamma=90.385(2)
Temperature:	296 K		

	Calculated	Reported
Volume	4737.9(2)	4737.9(2)
Space group	P -1	P -1
Hall group	-P 1	-P 1
Moiety formula	2(O65 Te Tm W17), 4(C2 N), 3(O) [+ solvent]	?
Sum formula	C8 N4 O133 Te2 Tm2 W34 [+ solvent]	C14 H108 N7 O86 Te Tm W17 Na2
Mr	9123.74	5218.80
Dx,g cm-3	3.198	3.658
Z	1	2
Mu (mm-1)	21.845	21.845
F000	3898.0	3898.0
F000’	3875.05	
h,k,lmax	19,21,36	19,21,35
Nref	31848	27838
Tmin,Tmax	0.007,0.113	0.007,0.113
Tmin’	0.001	

Correction method= # Reported T Limits: Tmin=0.007 Tmax=0.113
AbsCorr = MULTI-SCAN

Data completeness= 0.874 Theta(max)= 31.613

R(reflections)= 0.0452(20966) wR2(reflections)= 0.1160(27838)

S = 1.100 Npar= 829

The following ALERTS were generated. Each ALERT has the format
test-name_ALERT_alert-type_alert-level.
Click on the hyperlinks for more details of the test.

[IMAGE] **Alert level B**
PLAT097_ALERT_2_B Large Reported Max. (Positive) Residual Density 7.59 eA-3

PLAT306_ALERT_2_B Isolated Oxygen Atom (H-atoms Missing ?) .... O66 Check

---

[IMAGE] **Alert level C**

DIFMX02_ALERT_1_C The maximum difference density is > 0.1*ZMAX*0.75
The relevant atom site should be identified.
PLAT094_ALERT_2_C Ratio of Maximum / Minimum Residual Density .... 2.96 Report
PLAT242_ALERT_2_C Low 'MainMol' Ueq as Compared to Neighbors of Tm1 Check
PLAT244_ALERT_4_C Low 'Solvent' Ueq as Compared to Neighbors of N1 Check

---

[IMAGE] **Alert level G**

FORMU01_ALERT_2_G There is a discrepancy between the atom counts in the
_chemical_formula_sum and the formula from the _atom_site* data.
Atom count from _chemical_formula_sum:C14 H108 N7 Na2 O86 Te1 Tm1 W17
Atom count from the _atom_site data: C4 N2 O66.5 Te1 Tm1 W17
CELLZ01_ALERT_1_G Difference between formula and atom_site contents detected.
CELLZ01_ALERT_1_G ALERT: Large difference may be due to a
symmetry error - see SYMMG tests
From the CIF: _cell_formula_units_Z 2
From the CIF: _chemical_formula_sum C14 H108 N7 O86 Te Tm W17 Na2
TEST: Compare cell contents of formula and atom_site data

atom	Z*formula	cif sites	diff
C	28.00	8.00	20.00
H	216.00	0.00	216.00
N	14.00	4.00	10.00
O	172.00	133.00	39.00
Te	2.00	2.00	0.00
Tm	2.00	2.00	0.00
W	34.00	34.00	0.00
Na	4.00	0.00	4.00

PLAT003_ALERT_2_G Number of Uiso or Uij Restrained non-H Atoms ... 1 Report
PLAT012_ALERT_1_G N.O.K. _shelx_res_checksum Found in CIF ...... Please Check
PLAT040_ALERT_1_G No H-atoms in this Carbon Containing Compound .. Please Check
PLAT041_ALERT_1_G Calc. and Reported SumFormula Strings Differ Please Check
PLAT045_ALERT_1_G Calculated and Reported Z Differ by a Factor ... 0.50 Check
PLAT066_ALERT_1_G Predicted and Reported Tmin&Tmax Range Identical ? Check
PLAT083_ALERT_2_G SHELXL Second Parameter in WGHT Unusually Large 8.60 Why ?
PLAT154_ALERT_1_G The s.u.'s on the Cell Angles are Equal ..(Note) 0.002 Degree
PLAT186_ALERT_4_G The CIF-Embedded .res File Contains ISOR Records 1 Report
PLAT300_ALERT_4_G Atom Site Occupancy of O67 Constrained at 0.5 Check
PLAT302_ALERT_4_G Anion/Solvent/Minor-Residue Disorder (Resd 5) 100% Note
PLAT304_ALERT_4_G Non-Integer Number of Atoms in ..... (Resd 5) 0.50 Check
PLAT311_ALERT_2_G Isolated Disordered Oxygen Atom (No H's ?) ..... O67 Check
PLAT606_ALERT_4_G Solvent Accessible VOID(S) in Structure .... ! Info
PLAT720_ALERT_4_G Number of Unusual/Non-Standard Labels .... 1 Note
PLAT790_ALERT_4_G Centre of Gravity not Within Unit Cell: Resd. # 5 Note
O
PLAT794_ALERT_5_G Tentative Bond Valency for W1 (VI) . 6.16 Info
PLAT794_ALERT_5_G Tentative Bond Valency for W2 (VI) . 6.17 Info
PLAT794_ALERT_5_G Tentative Bond Valency for W3 (VI) . 6.06 Info
PLAT794_ALERT_5_G Tentative Bond Valency for W4 (VI) . 6.18 Info
PLAT794_ALERT_5_G Tentative Bond Valency for W5 (VI) . 6.06 Info
PLAT794_ALERT_5_G Tentative Bond Valency for W6 (VI) . 6.20 Info
PLAT794_ALERT_5_G Tentative Bond Valency for W7 (VI) . 6.14 Info
PLAT794_ALERT_5_G Tentative Bond Valency for W8 (VI) . 6.22 Info
PLAT794_ALERT_5_G Tentative Bond Valency for W9 (VI) . 6.24 Info
PLAT794_ALERT_5_G Tentative Bond Valency for W10 (VI) . 6.16 Info
PLAT794_ALERT_5_G Tentative Bond Valency for W11 (VI) . 6.30 Info
PLAT794_ALERT_5_G Tentative Bond Valency for W12 (VI) . 6.19 Info
PLAT794_ALERT_5_G Tentative Bond Valency for W13 (VI) . 6.08 Info

```
PLAT794_ALERT_5_G Tentative Bond Valency for W14       (VI)      .       6.25 Info
PLAT794_ALERT_5_G Tentative Bond Valency for W15       (VI)      .       5.96 Info
PLAT794_ALERT_5_G Tentative Bond Valency for W16       (VI)      .       6.19 Info
PLAT794_ALERT_5_G Tentative Bond Valency for W17       (VI)      .       6.13 Info
PLAT794_ALERT_5_G Tentative Bond Valency for Tm1       (III)     .       3.27 Info
PLAT794_ALERT_5_G Tentative Bond Valency for Te1       (VI)      .       5.36 Info
PLAT860_ALERT_3_G Number of Least-Squares Restraints .............          6 Note
PLAT868_ALERT_4_G ALERTS Due to the Use of _smtbx_masks Suppressed          ! Info
PLAT933_ALERT_2_G Number of OMIT Records in Embedded .res File ...          3 Note
PLAT941_ALERT_3_G Average HKL Measurement Multiplicity ...........        2.2 Low
```

---

 0 **ALERT level A** = Most likely a serious problem - resolve or explain
 2 **ALERT level B** = A potentially serious problem, consider carefully
 4 **ALERT level C** = Check. Ensure it is not caused by an omission or oversight
42 **ALERT level G** = General information/check it is not something unexpected

 9 ALERT type 1 CIF construction/syntax error, inconsistent or missing data
 9 ALERT type 2 Indicator that the structure model may be wrong or deficient
 2 ALERT type 3 Indicator that the structure quality may be low
 9 ALERT type 4 Improvement, methodology, query or suggestion
19 ALERT type 5 Informative message, check

Datablock: compound_monosubstituted_Yb

Bond precision: N- C = 0.0188 A   Wavelength=0.71073

Cell:	a=13.1532(3)	b=14.8728(3)	c=24.9642(4)
	alpha=102.392(1)	beta=96.258(2)	gamma=90.499(2)
Temperature:	296 K		

	Calculated	Reported
Volume	4738.83(17)	4738.83(17)
Space group	P -1	P -1
Hall group	-P 1	-P 1
Moiety formula	O65 Te W17 Yb, 2(C2 N), 2(O) [+ solvent]	?
Sum formula	C4 N2 O67 Te W17 Yb [+ solvent]	C14 H118 N7 O91 Te W17 Yb Na2
Mr	4573.98	5312.98
Dx,g cm-3	3.206	3.723
Z	2	2
Mu (mm-1)	21.892	21.892
F000	3908.0	3908.0
F000’	3884.94	
h,k,lmax	19,21,36	19,21,35
Nref	31691	27699
Tmin,Tmax	0.007,0.112	0.007,0.112
Tmin’	0.001	

Correction method= # Reported T Limits: Tmin=0.007 Tmax=0.112
AbsCorr = MULTI-SCAN

Data completeness= 0.874   Theta(max)= 31.555

R(reflections)= 0.0448(20691)   wR2(reflections)= 0.1085(27699)

S = 1.093   Npar= 829

The following ALERTS were generated. Each ALERT has the format
test-name_ALERT_alert-type_alert-level.
Click on the hyperlinks for more details of the test.

[IMAGE] **Alert level B**

```
PLAT306_ALERT_2_B Isolated Oxygen Atom (H-atoms Missing ?) .......        O66 Check
PLAT306_ALERT_2_B Isolated Oxygen Atom (H-atoms Missing ?) .......        O67 Check
```

[IMAGE] **Alert level C**

```
DIFMX02_ALERT_1_C  The maximum difference density is > 0.1*ZMAX*0.75
            The relevant atom site should be identified.
PLAT097_ALERT_2_C Large Reported Max.  (Positive) Residual Density       5.74 eA-3
PLAT220_ALERT_2_C NonSolvent   Resd 1  O   Ueq(max)/Ueq(min) Range        3.2 Ratio
PLAT242_ALERT_2_C Low    'MainMol' Ueq as Compared to Neighbors of        Yb1 Check
```

[IMAGE] **Alert level G**

```
FORMU01_ALERT_2_G  There is a discrepancy between the atom counts in the
            _chemical_formula_sum and the formula from the _atom_site* data.
            Atom count from _chemical_formula_sum:C14 H118 N7 Na2 O91 Te1 W17 Yb1
            Atom count from the _atom_site data:  C4 N2 O67 Te1 W17 Yb1
CELLZ01_ALERT_1_G Difference between formula and atom_site contents detected.
CELLZ01_ALERT_1_G ALERT: Large difference may be due to a
            symmetry error - see SYMMG tests
           From the CIF: _cell_formula_units_Z    2
           From the CIF: _chemical_formula_sum  C14 H118 N7 O91 Te W17 Yb Na2
           TEST: Compare cell contents of formula and atom_site data

           atom    Z*formula  cif sites diff
           C         28.00      8.00   20.00
           H        236.00      0.00  236.00
           N         14.00      4.00   10.00
           O        182.00    134.00   48.00
           Te         2.00      2.00    0.00
           W         34.00     34.00    0.00
           Yb         2.00      2.00    0.00
           Na         4.00      0.00    4.00
PLAT003_ALERT_2_G Number of Uiso or Uij Restrained non-H Atoms ...          1 Report
PLAT012_ALERT_1_G N.O.K.   _shelx_res_checksum Found in CIF ......     Please Check
PLAT040_ALERT_1_G No H-atoms in this Carbon Containing Compound ..     Please Check
PLAT041_ALERT_1_G Calc. and Reported SumFormula    Strings  Differ     Please Check
PLAT066_ALERT_1_G Predicted and Reported Tmin&Tmax Range Identical          ? Check
PLAT186_ALERT_4_G The CIF-Embedded .res File Contains ISOR Records          1 Report
PLAT606_ALERT_4_G Solvent Accessible VOID(S) in Structure ........          ! Info
PLAT790_ALERT_4_G Centre of Gravity not Within Unit Cell: Resd.  #          4 Note
              O
PLAT794_ALERT_5_G Tentative Bond Valency for W1        (VI)      .       6.31 Info
```

```
PLAT794_ALERT_5_G Tentative Bond Valency for W2        (VI)      .       6.19 Info
PLAT794_ALERT_5_G Tentative Bond Valency for W3        (VI)      .       6.07 Info
PLAT794_ALERT_5_G Tentative Bond Valency for W4        (VI)      .       6.22 Info
PLAT794_ALERT_5_G Tentative Bond Valency for W5        (VI)      .       6.16 Info
PLAT794_ALERT_5_G Tentative Bond Valency for W6        (VI)      .       6.12 Info
PLAT794_ALERT_5_G Tentative Bond Valency for W7        (VI)      .       6.13 Info
PLAT794_ALERT_5_G Tentative Bond Valency for W8        (VI)      .       6.15 Info
PLAT794_ALERT_5_G Tentative Bond Valency for W9        (VI)      .       6.13 Info
PLAT794_ALERT_5_G Tentative Bond Valency for W10       (VI)      .       6.12 Info
PLAT794_ALERT_5_G Tentative Bond Valency for W11       (VI)      .       6.25 Info
PLAT794_ALERT_5_G Tentative Bond Valency for W12       (VI)      .       6.24 Info
PLAT794_ALERT_5_G Tentative Bond Valency for W13       (VI)      .       6.18 Info
PLAT794_ALERT_5_G Tentative Bond Valency for W14       (VI)      .       6.08 Info
PLAT794_ALERT_5_G Tentative Bond Valency for W15       (VI)      .       5.90 Info
PLAT794_ALERT_5_G Tentative Bond Valency for W16       (VI)      .       6.10 Info
PLAT794_ALERT_5_G Tentative Bond Valency for W17       (VI)      .       6.19 Info
PLAT794_ALERT_5_G Tentative Bond Valency for Yb1       (III)     .       3.00 Info
PLAT794_ALERT_5_G Tentative Bond Valency for Te1       (VI)      .       5.31 Info
PLAT860_ALERT_3_G Number of Least-Squares Restraints .............          6 Note
PLAT868_ALERT_4_G ALERTS Due to the Use of _smtbx_masks Suppressed          ! Info
PLAT933_ALERT_2_G Number of OMIT Records in Embedded .res File ...         13 Note
PLAT941_ALERT_3_G Average HKL Measurement Multiplicity ...........        2.2 Low
```

---

   0 **ALERT level A** = Most likely a serious problem - resolve or explain
   2 **ALERT level B** = A potentially serious problem, consider carefully
   4 **ALERT level C** = Check. Ensure it is not caused by an omission or oversight
  34 **ALERT level G** = General information/check it is not something unexpected

   7 ALERT type 1 CIF construction/syntax error, inconsistent or missing data
   8 ALERT type 2 Indicator that the structure model may be wrong or deficient
   2 ALERT type 3 Indicator that the structure quality may be low
   4 ALERT type 4 Improvement, methodology, query or suggestion
  19 ALERT type 5 Informative message, check

Datablock: compound_monosubstituted_Lu

Bond precision: N- C = 0.0200 A Wavelength=0.71073

Cell:	a=13.1283(3)	b=14.8448(4)	c=24.9474(6)
	alpha=102.527(2)	beta=96.464(2)	gamma=90.290(2)
Temperature:	296 K		

	Calculated	Reported
Volume	4713.8(2)	4713.8(2)
Space group	P -1	P -1
Hall group	-P 1	-P 1
Moiety formula	Lu O65 Te W17, C2 N, O [+ solvent]	?
Sum formula	C2 Lu N O66 Te W17 [+ solvent]	C14 H120 Lu N7 O92 Te W17 Na2
Mr	4521.88	5332.92
Dx,g cm-3	3.186	3.757
Z	2	2
Mu (mm-1)	22.061	22.061
F000	3856.0	3856.0
F000'	3832.88	
h,k,lmax	19,21,36	19,21,36
Nref	31735	27781
Tmin,Tmax	0.007,0.110	0.007,0.110
Tmin'	0.001	

Correction method= # Reported T Limits: Tmin=0.007 Tmax=0.110
AbsCorr = MULTI-SCAN

Data completeness= 0.875 Theta(max)= 31.633

R(reflections)= 0.0557(18880) wR2(reflections)= 0.1294(27781)

S = 1.045 Npar= 793

The following ALERTS were generated. Each ALERT has the format
test-name_ALERT_alert-type_alert-level.
Click on the hyperlinks for more details of the test.

[IMAGE] **Alert level B**

```
PLAT097_ALERT_2_B Large Reported Max.  (Positive) Residual Density       7.88 eA-3
PLAT306_ALERT_2_B Isolated Oxygen Atom (H-atoms Missing ?) .......         O66 Check
```

[IMAGE] **Alert level C**

```
DIFMX02_ALERT_1_C  The maximum difference density is > 0.1*ZMAX*0.75
            The relevant atom site should be identified.
PLAT094_ALERT_2_C Ratio of Maximum / Minimum Residual Density ....       2.04 Report
PLAT213_ALERT_2_C Atom O31             has ADP max/min Ratio .....        3.9 oblate
PLAT220_ALERT_2_C NonSolvent   Resd 1  O   Ueq(max)/Ueq(min) Range        3.6 Ratio
```

[IMAGE] **Alert level G**

```
FORMU01_ALERT_2_G  There is a discrepancy between the atom counts in the
            _chemical_formula_sum and the formula from the _atom_site* data.
            Atom count from _chemical_formula_sum:C14 H120 Lu1 N7 Na2 O92 Te1 W17
            Atom count from the _atom_site data:  C2 Lu1 N1 O66 Te1 W17
CELLZ01_ALERT_1_G Difference between formula and atom_site contents detected.
CELLZ01_ALERT_1_G ALERT: Large difference may be due to a
```

```
            symmetry error - see SYMMG tests
           From the CIF: _cell_formula_units_Z    2
           From the CIF: _chemical_formula_sum  C14 H120 Lu N7 O92 Te W17 Na2
           TEST: Compare cell contents of formula and atom_site data

           atom    Z*formula  cif sites diff
           C         28.00      4.00   24.00
           H        240.00      0.00  240.00
           Lu         2.00      2.00    0.00
           N         14.00      2.00   12.00
           O        184.00    132.00   52.00
           Te         2.00      2.00    0.00
           W         34.00     34.00    0.00
           Na         4.00      0.00    4.00
PLAT003_ALERT_2_G Number of Uiso or Uij Restrained non-H Atoms ...          4 Report
PLAT012_ALERT_1_G N.O.K.   _shelx_res_checksum Found in CIF ......     Please Check
PLAT040_ALERT_1_G No H-atoms in this Carbon Containing Compound ..     Please Check
PLAT041_ALERT_1_G Calc. and Reported SumFormula    Strings  Differ     Please Check
PLAT066_ALERT_1_G Predicted and Reported Tmin&Tmax Range Identical          ? Check
PLAT154_ALERT_1_G The s.u.'s on the Cell Angles are Equal ..(Note)      0.002 Degree
PLAT186_ALERT_4_G The CIF-Embedded .res File Contains ISOR Records          4 Report
PLAT606_ALERT_4_G Solvent Accessible VOID(S) in Structure ........          ! Info
PLAT794_ALERT_5_G Tentative Bond Valency for W1       (VI)      .       6.42 Info
PLAT794_ALERT_5_G Tentative Bond Valency for W2       (VI)      .       6.27 Info
PLAT794_ALERT_5_G Tentative Bond Valency for W3       (VI)      .       6.05 Info
PLAT794_ALERT_5_G Tentative Bond Valency for W4       (VI)      .       6.23 Info
PLAT794_ALERT_5_G Tentative Bond Valency for W5       (VI)      .       6.19 Info
PLAT794_ALERT_5_G Tentative Bond Valency for W6       (VI)      .       6.15 Info
PLAT794_ALERT_5_G Tentative Bond Valency for W7       (VI)      .       6.14 Info
PLAT794_ALERT_5_G Tentative Bond Valency for W8       (VI)      .       6.31 Info
PLAT794_ALERT_5_G Tentative Bond Valency for W9       (VI)      .       6.20 Info
PLAT794_ALERT_5_G Tentative Bond Valency for W10      (VI)      .       6.37 Info
PLAT794_ALERT_5_G Tentative Bond Valency for W11      (VI)      .       6.22 Info
PLAT794_ALERT_5_G Tentative Bond Valency for W12      (VI)      .       6.15 Info
PLAT794_ALERT_5_G Tentative Bond Valency for W13      (VI)      .       6.17 Info
PLAT794_ALERT_5_G Tentative Bond Valency for W14      (VI)      .       6.09 Info
PLAT794_ALERT_5_G Tentative Bond Valency for W15      (VI)      .       6.19 Info
PLAT794_ALERT_5_G Tentative Bond Valency for W16      (VI)      .       6.20 Info
PLAT794_ALERT_5_G Tentative Bond Valency for W17      (VI)      .       6.11 Info
PLAT794_ALERT_5_G Tentative Bond Valency for Lu1      (III)     .       3.15 Info
PLAT794_ALERT_5_G Tentative Bond Valency for Te1      (VI)      .       5.33 Info
PLAT860_ALERT_3_G Number of Least-Squares Restraints .............         24 Note
PLAT868_ALERT_4_G ALERTS Due to the Use of _smtbx_masks Suppressed          ! Info
PLAT933_ALERT_2_G Number of OMIT Records in Embedded .res File ...          2 Note
PLAT941_ALERT_3_G Average HKL Measurement Multiplicity ...........        2.2 Low
```

---

   0 **ALERT level A** = Most likely a serious problem - resolve or explain
   2 **ALERT level B** = A potentially serious problem, consider carefully
   4 **ALERT level C** = Check. Ensure it is not caused by an omission or oversight
  34 **ALERT level G** = General information/check it is not something unexpected

```
   8 ALERT type 1 CIF construction/syntax error, inconsistent or missing data
   8 ALERT type 2 Indicator that the structure model may be wrong or deficient
   2 ALERT type 3 Indicator that the structure quality may be low
   3 ALERT type 4 Improvement, methodology, query or suggestion
  19 ALERT type 5 Informative message, check
```

It is advisable to attempt to resolve as many as possible of the alerts in all categories. Often the minor alerts point to easily fixed oversights, errors and omissions in your CIF or refinement strategy, so attention to these fine details can be worthwhile. In order to resolve some of the more serious problems it may be necessary to carry out additional measurements or structure refinements. However, the purpose of your study may justify the reported deviations and the more serious of these should normally be commented upon in the discussion or experimental section of a paper or in the "special_details" fields of the CIF. checkCIF was carefully designed to identify outliers and unusual parameters, but every test has its limitations and alerts that are not important in a particular case may appear. Conversely, the absence of alerts does not guarantee there are no aspects of the results needing attention. It is up to the individual to critically assess their own results and, if necessary, seek expert advice.

Publication of your CIF in IUCr journals

A basic structural check has been run on your CIF. These basic checks will be run on all CIFs submitted for publication in IUCr journals (*Acta Crystallographica*, *Journal of Applied Crystallography*, *Journal of Synchrotron Radiation*); however, if you intend to submit to *Acta Crystallographica Section C* or *E* or *IUCrData*, you should make sure that full publication checks are run on the final version of your CIF prior to submission.

Publication of your CIF in other journals

Please refer to the *Notes for Authors* of the relevant journal for any special instructions relating to CIF submission.

PLATON version of 10/08/2020; check.def file version of 06/08/2020

Datablock compound_monosubstituted_Dy - ellipsoid plot

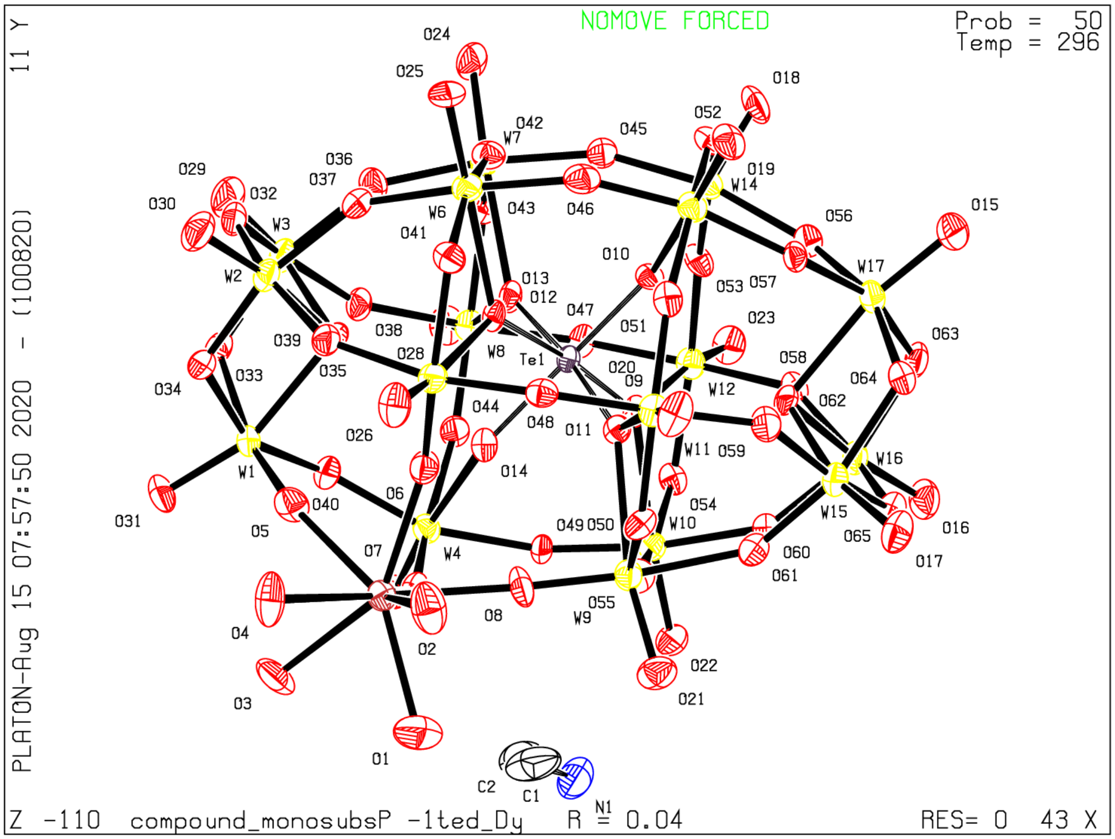

Datablock compound_monosubstituted-Ho - ellipsoid plot

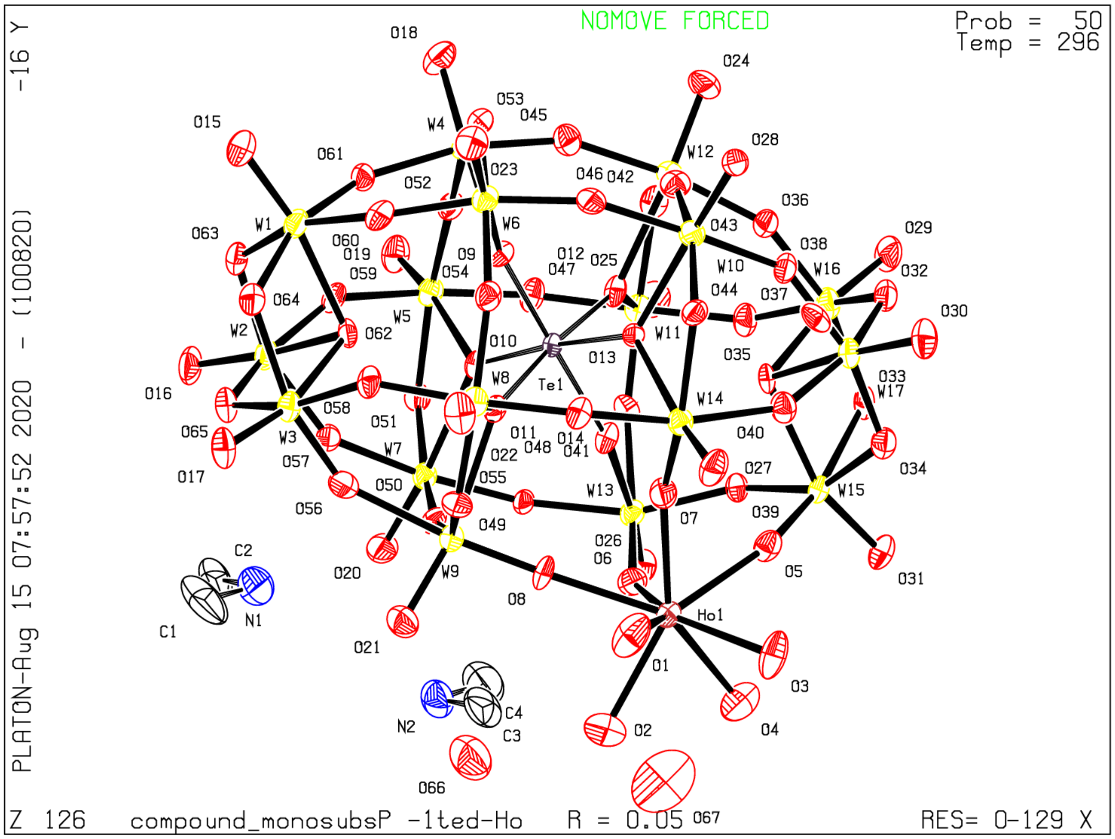

Datablock compound_monosubstituted_Er - ellipsoid plot

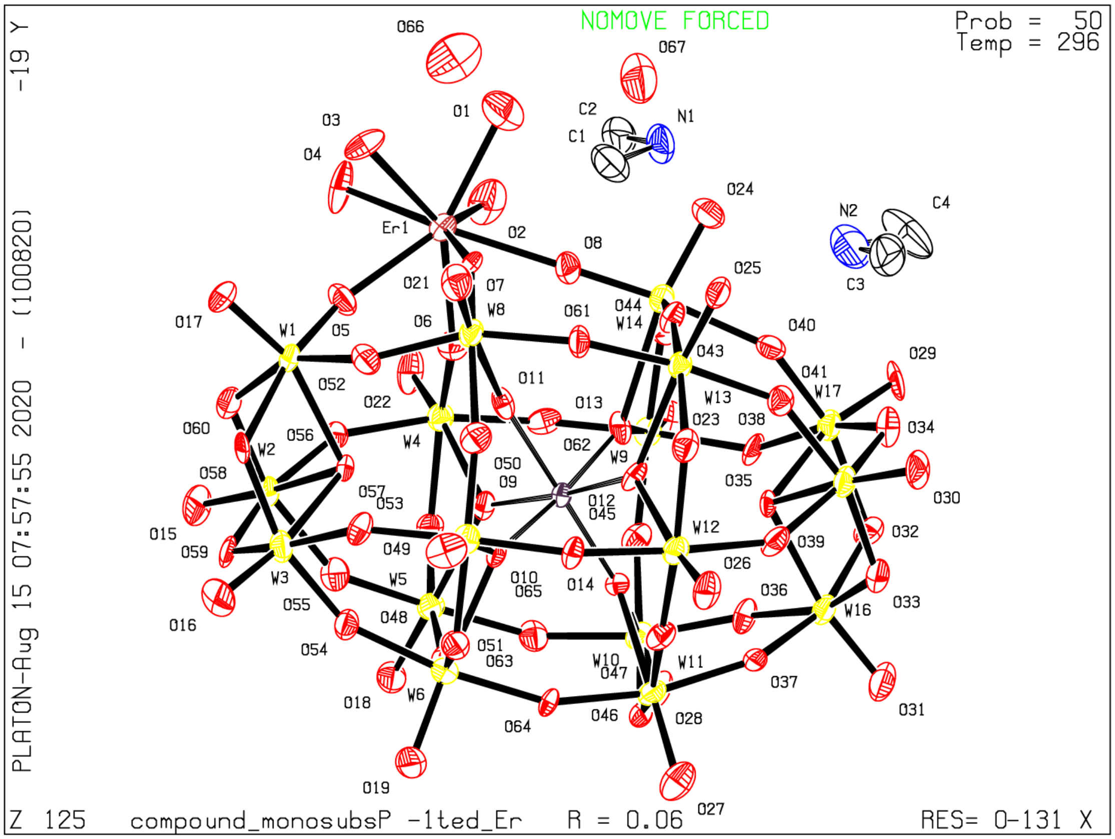

Datablock compound_monosubstituted_Tm - ellipsoid plot

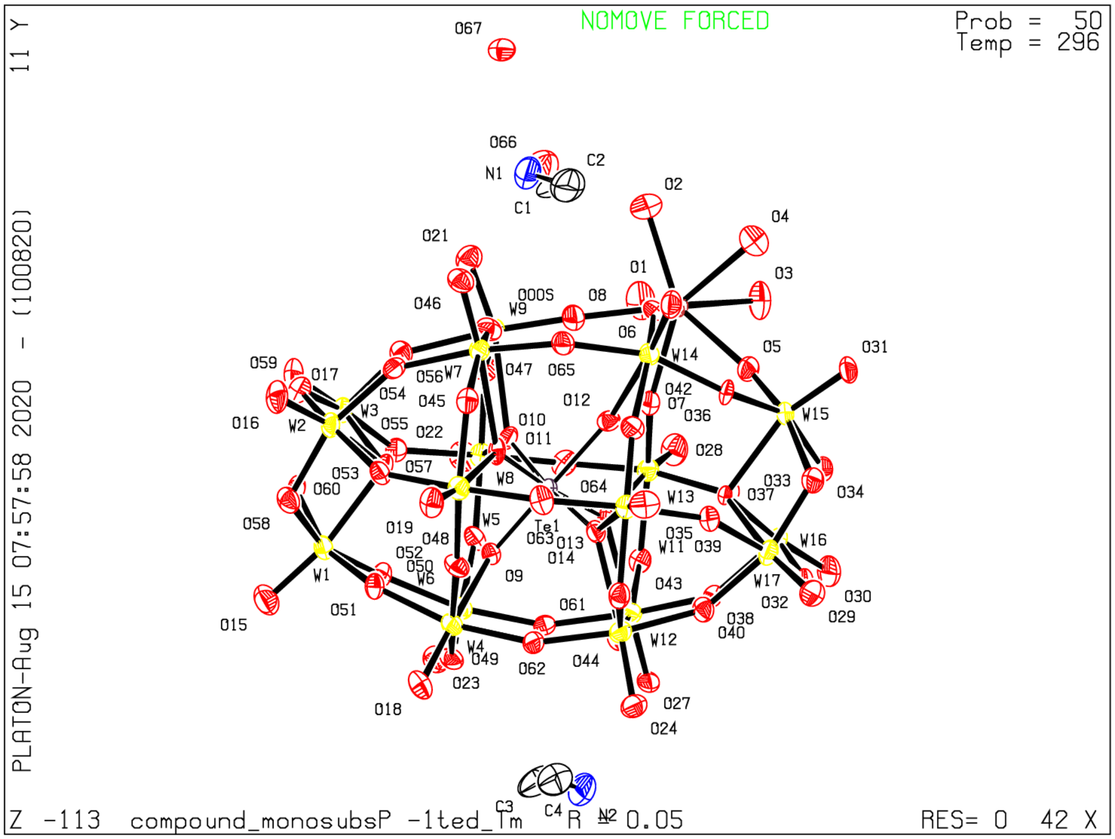

Datablock compound_monosubstituted_Yb - ellipsoid plot

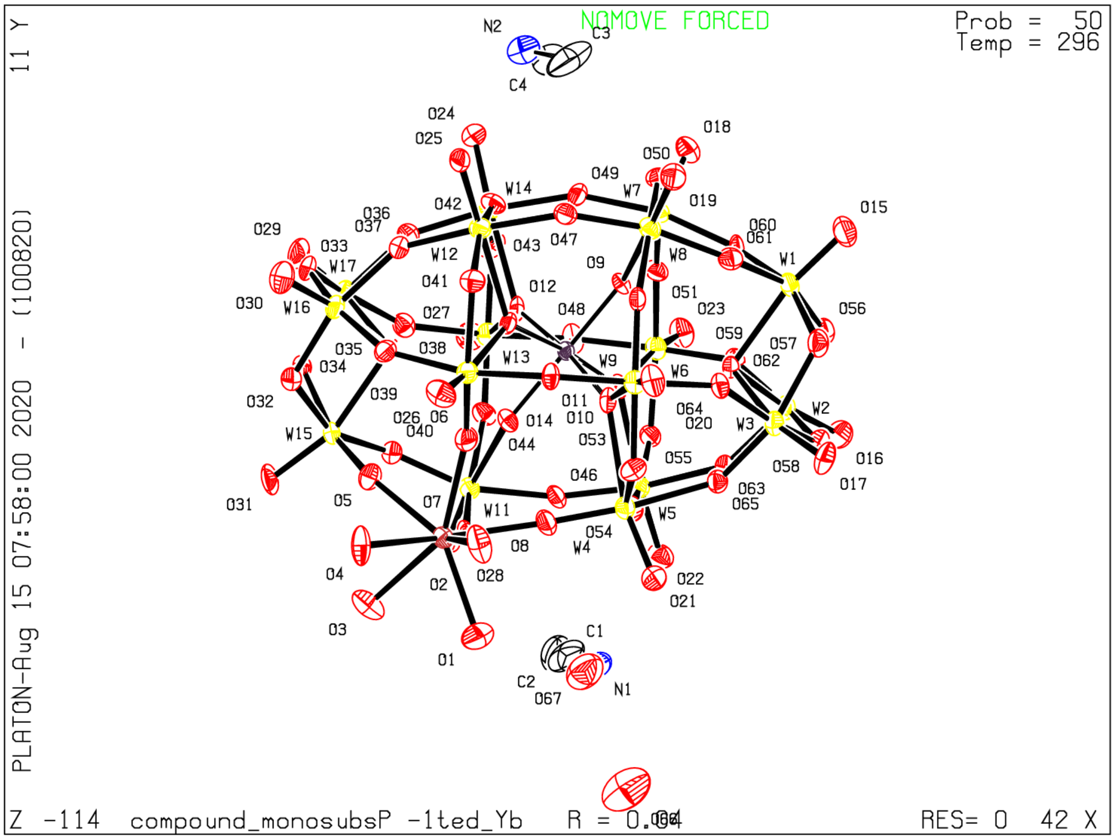

Datablock compound_monosubstituted_Lu - ellipsoid plot

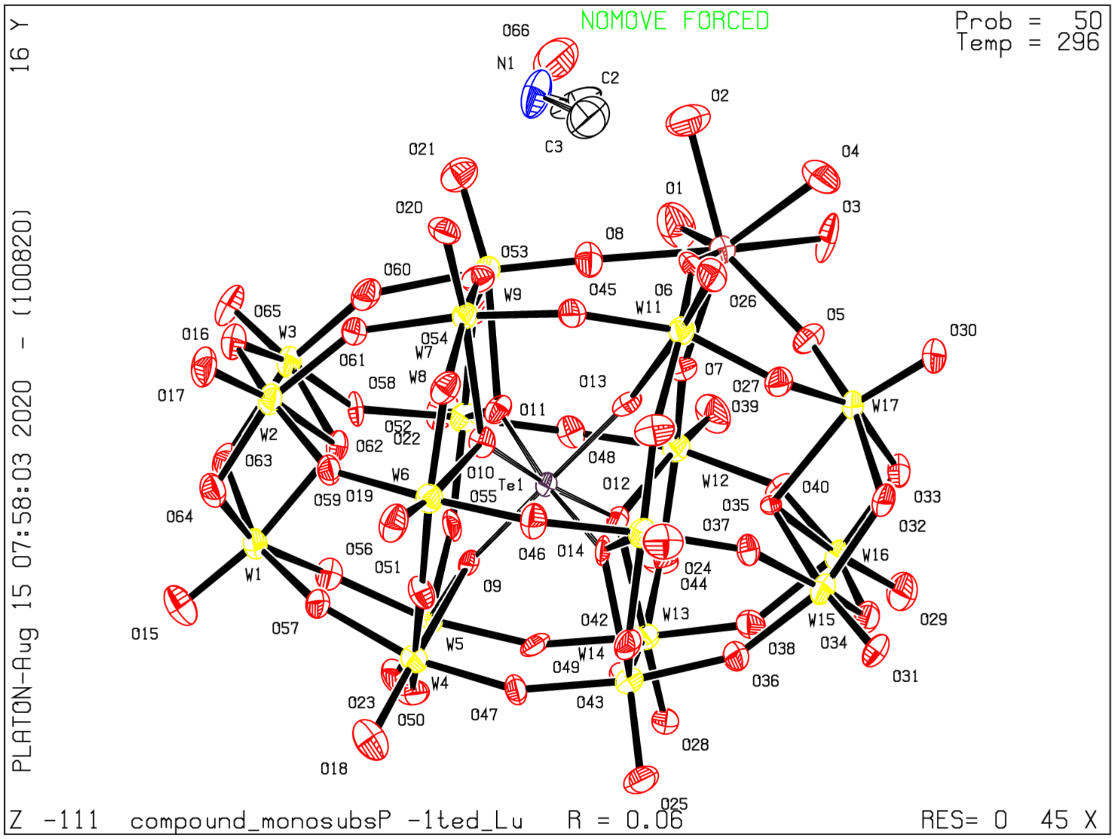

Supplement: Supplementary file 3 [file Data_Sheet_2.PDF]
