# Supplementary material for: Assembly of Lanthanide-Containing Tungstotellurates(VI): Syntheses, Structures, and Catalytic Properties
Source: Front Chem. 2020 Nov 23;8:598961. doi: 10.3389/fchem.2020.598961 (PMC7719746; doi:10.3389/fchem.2020.598961)

checkCIF/PLATON report

You have not supplied any structure factors. As a result the full set of tests cannot be run.

THIS REPORT IS FOR GUIDANCE ONLY. IF USED AS PART OF A REVIEW PROCEDURE FOR PUBLICATION, IT SHOULD NOT REPLACE THE EXPERTISE OF AN EXPERIENCED CRYSTALLOGRAPHIC REFEREE.

No syntax errors found. CIF dictionary Interpreting this report

Datablock: compound_3D_Dy

```
Bond precision:  Dy- O = 0.0040 A        Wavelength=0.71073

Cell:            a=13.6902(4)     b=17.6372(7)     c=15.6832(6)
                 alpha=90         beta=90          gamma=90
Temperature:     296 K
```

	Calculated	Reported
Volume	3786.8(2)	3786.8(2)
Space group	C c c m	C c c m
Hall group	-C 2 2c	-C 2 2c
Moiety formula	Dy2 O56 Te2 W12, 2(C2 N), 2(O) [+ solvent]	?
Sum formula	C4 Dy2 N2 O58 Te2 W12 [+ solvent]	C2 H26 Dy N O32 Te W6
Mr	3790.34	1969.35
Dx,g cm-3	3.324	3.454
Z	2	4
Mu (mm-1)	20.930	20.930
F000	3252.0	3252.0
F000’	3234.23	
h,k,lmax	20,25,23	19,25,23
Nref	3276	3124
Tmin,Tmax	0.009,0.123	0.009,0.123
Tmin’	0.001	

```
Correction method= # Reported T Limits: Tmin=0.009 Tmax=0.123
AbsCorr = MULTI-SCAN

Data completeness= 0.954          Theta(max)= 31.512

R(reflections)= 0.0276( 2840)     wR2(reflections)= 0.0647( 3124)

S = 1.053                   Npar= 112
```

The following ALERTS were generated. Each ALERT has the format
test-name_ALERT_alert-type_alert-level.
Click on the hyperlinks for more details of the test.

[IMAGE] **Alert level G**

FORMU01_ALERT_2_G There is a discrepancy between the atom counts in the _chemical_formula_sum and the formula from the _atom_site* data.
Atom count from _chemical_formula_sum:C2 H26 Dy1 N1 O32 Te1 W6
Atom count from the _atom_site data: C2 Dy1 N1 O29 Te1 W6
CELLZ01_ALERT_1_G Difference between formula and atom_site contents detected.
CELLZ01_ALERT_1_G ALERT: Large difference may be due to a symmetry error - see SYMMG tests
From the CIF: _cell_formula_units_Z 4
From the CIF: _chemical_formula_sum C2 H26 Dy N O32 Te W6
TEST: Compare cell contents of formula and atom_site data

atom	Z*formula	cif sites	diff
C	8.00	8.00	0.00
H	104.00	0.00	104.00
Dy	4.00	4.00	0.00
N	4.00	4.00	0.00
O	128.00	116.00	12.00
Te	4.00	4.00	0.00
W	24.00	24.00	0.00

PLAT003_ALERT_2_G Number of Uiso or Uij Restrained non-H Atoms ... 3 Report
PLAT012_ALERT_1_G N.O.K. _shelx_res_checksum Found in CIF ...... Please Check
PLAT040_ALERT_1_G No H-atoms in this Carbon Containing Compound .. Please Check
PLAT041_ALERT_1_G Calc. and Reported SumFormula Strings Differ Please Check
PLAT045_ALERT_1_G Calculated and Reported Z Differ by a Factor ... 0.50 Check
PLAT066_ALERT_1_G Predicted and Reported Tmin&Tmax Range Identical ? Check
PLAT083_ALERT_2_G SHELXL Second Parameter in WGHT Unusually Large 53.43 Why ?
PLAT186_ALERT_4_G The CIF-Embedded .res File Contains ISOR Records 3 Report
PLAT300_ALERT_4_G Atom Site Occupancy of N1 Constrained at 0.5 Check
PLAT302_ALERT_4_G Anion/Solvent/Minor-Residue Disorder (Resd 2) 50% Note
PLAT302_ALERT_4_G Anion/Solvent/Minor-Residue Disorder (Resd 3) 100% Note
PLAT302_ALERT_4_G Anion/Solvent/Minor-Residue Disorder (Resd 4) 100% Note
PLAT304_ALERT_4_G Non-Integer Number of Atoms in ..... (Resd 3) 0.08 Check
PLAT304_ALERT_4_G Non-Integer Number of Atoms in ..... (Resd 4) 0.17 Check
PLAT311_ALERT_2_G Isolated Disordered Oxygen Atom (No H's ?) ..... O9 Check
PLAT311_ALERT_2_G Isolated Disordered Oxygen Atom (No H's ?) ..... O10 Check
PLAT605_ALERT_4_G Largest Solvent Accessible VOID in the Structure 600 A**3
PLAT794_ALERT_5_G Tentative Bond Valency for W1 (VI) . 6.00 Info
PLAT794_ALERT_5_G Tentative Bond Valency for W2 (VI) . 6.03 Info
PLAT794_ALERT_5_G Tentative Bond Valency for Dy1 (III) . 2.92 Info
PLAT794_ALERT_5_G Tentative Bond Valency for Te1 (VI) . 5.82 Info
PLAT860_ALERT_3_G Number of Least-Squares Restraints .... 18 Note
PLAT868_ALERT_4_G ALERTS Due to the Use of _smtbx_masks Suppressed ! Info
PLAT933_ALERT_2_G Number of OMIT Records in Embedded .res File ... 3 Note

---

0 **ALERT level A** = Most likely a serious problem - resolve or explain
0 **ALERT level B** = A potentially serious problem, consider carefully
0 **ALERT level C** = Check. Ensure it is not caused by an omission or oversight
27 **ALERT level G** = General information/check it is not something unexpected

7 ALERT type 1 CIF construction/syntax error, inconsistent or missing data
6 ALERT type 2 Indicator that the structure model may be wrong or deficient
1 ALERT type 3 Indicator that the structure quality may be low
9 ALERT type 4 Improvement, methodology, query or suggestion
4 ALERT type 5 Informative message, check

Datablock: compound_3D_Ho

Bond precision: Ho- O = 0.0070 A Wavelength=0.71073

Cell:	a=13.6884(4)	b=17.5689(5)	c=15.6792(4)
	alpha=90	beta=90	gamma=90
Temperature:	296 K		

	Calculated	Reported
Volume	3770.69(18)	3770.69(18)
Space group	C c c m	C c c m
Hall group	-C 2 2c	-C 2 2c
Moiety formula	Ho2 O56 Te2 W12, 2(C2 N), 2(O) [+ solvent]	?
Sum formula	C4 Ho2 N2 O58 Te2 W12 [+ solvent]	C4 H27 Ho N2 O27 Te W6
Mr	3795.20	1930.82
Dx,g cm-3	3.343	3.401
Z	2	4
Mu (mm-1)	21.136	21.136
F000	3256.0	3256.0
F000′	3238.15	
h,k,lmax	20,25,23	20,24,23
Nref	3249	3012
Tmin,Tmax	0.009,0.121	0.009,0.121
Tmin′	0.001	

Correction method= # Reported T Limits: Tmin=0.009 Tmax=0.121
AbsCorr = MULTI-SCAN

Data completeness= 0.927 Theta(max)= 31.488

R(reflections)= 0.0440(2840) wR2(reflections)= 0.1113(3012)

S = 1.121 Npar= 112

The following ALERTS were generated. Each ALERT has the format
test-name_ALERT_alert-type_alert-level.
Click on the hyperlinks for more details of the test.

[IMAGE] **Alert level C**

DIFMX02_ALERT_1_C The maximum difference density is > 0.1*ZMAX*0.75
The relevant atom site should be identified.
PLAT097_ALERT_2_C Large Reported Max. (Positive) Residual Density 5.90 eA-3

[IMAGE] **Alert level G**

FORMU01_ALERT_2_G There is a discrepancy between the atom counts in the

```
            _chemical_formula_sum and the formula from the _atom_site* data.
            Atom count from _chemical_formula_sum:C4 H27 Ho1 N2 O27 Te1 W6
            Atom count from the _atom_site data:  C2 Ho1 N1 O29 Te1 W6
CELLZ01_ALERT_1_G Difference between formula and atom_site contents detected.
CELLZ01_ALERT_1_G ALERT: Large difference may be due to a
            symmetry error - see SYMMG tests
           From the CIF: _cell_formula_units_Z    4
           From the CIF: _chemical_formula_sum  C4 H27 Ho N2 O27 Te W6
           TEST: Compare cell contents of formula and atom_site data

           atom    Z*formula  cif sites diff
           C         16.00      8.00    8.00
           H        108.00      0.00  108.00
           Ho         4.00      4.00    0.00
           N          8.00      4.00    4.00
           O        108.00    116.00   -8.00
           Te         4.00      4.00    0.00
           W         24.00     24.00    0.00
PLAT003_ALERT_2_G Number of Uiso or Uij Restrained non-H Atoms ...          6 Report
PLAT012_ALERT_1_G N.O.K.   _shelx_res_checksum Found in CIF ......     Please Check
PLAT040_ALERT_1_G No H-atoms in this Carbon Containing Compound ..     Please Check
PLAT041_ALERT_1_G Calc. and Reported SumFormula    Strings  Differ     Please Check
PLAT045_ALERT_1_G Calculated and Reported Z Differ by a Factor ...       0.50 Check
PLAT066_ALERT_1_G Predicted and Reported Tmin&Tmax Range Identical          ? Check
PLAT083_ALERT_2_G SHELXL Second Parameter in WGHT  Unusually Large     192.08 Why ?
PLAT186_ALERT_4_G The CIF-Embedded .res File Contains ISOR Records          4 Report
PLAT300_ALERT_4_G Atom Site Occupancy of N1        Constrained at        0.5 Check
PLAT302_ALERT_4_G Anion/Solvent/Minor-Residue Disorder (Resd  2  )        50% Note
PLAT302_ALERT_4_G Anion/Solvent/Minor-Residue Disorder (Resd  3  )       100% Note
PLAT302_ALERT_4_G Anion/Solvent/Minor-Residue Disorder (Resd  4  )       100% Note
PLAT304_ALERT_4_G Non-Integer Number of Atoms in ..... (Resd  3  )       0.16 Check
PLAT304_ALERT_4_G Non-Integer Number of Atoms in ..... (Resd  4  )       0.09 Check
PLAT311_ALERT_2_G Isolated Disordered Oxygen Atom (No H's ?) .....         O9 Check
PLAT311_ALERT_2_G Isolated Disordered Oxygen Atom (No H's ?) .....        O10 Check
PLAT605_ALERT_4_G Largest Solvent Accessible VOID in the Structure        595 A**3
PLAT794_ALERT_5_G Tentative Bond Valency for W1        (VI)      .       6.06 Info
PLAT794_ALERT_5_G Tentative Bond Valency for W2        (VI)      .       5.98 Info
PLAT794_ALERT_5_G Tentative Bond Valency for Ho1       (III)     .       3.20 Info
PLAT794_ALERT_5_G Tentative Bond Valency for Te1       (VI)      .       5.72 Info
PLAT860_ALERT_3_G Number of Least-Squares Restraints ............          36 Note
PLAT868_ALERT_4_G ALERTS Due to the Use of _smtbx_masks Suppressed          ! Info
```

```
   0 ALERT level A = Most likely a serious problem - resolve or explain
   0 ALERT level B = A potentially serious problem, consider carefully
   2 ALERT level C = Check. Ensure it is not caused by an omission or oversight
  26 ALERT level G = General information/check it is not something unexpected

   8 ALERT type 1 CIF construction/syntax error, inconsistent or missing data
   6 ALERT type 2 Indicator that the structure model may be wrong or deficient
   1 ALERT type 3 Indicator that the structure quality may be low
   9 ALERT type 4 Improvement, methodology, query or suggestion
   4 ALERT type 5 Informative message, check
```

Datablock: compound_3D_Er

Bond precision: W- O = 0.0037 A   Wavelength=0.71073

Cell:	a=13.6976(6)	b=17.4564(6)	c=15.6222(6)
	alpha=90	beta=90	gamma=90
Temperature:	296 K		

	Calculated	Reported
Volume	3735.4(3)	3735.4(3)
Space group	C c c m	C c c m
Hall group	-C 2 2c	-C 2 2c
Moiety formula	Er2 O56 Te2 W12, O [+ solvent]	?
Sum formula	Er2 O57 Te2 W12 [+ solvent]	C2 H38 Er N O38 Te W6
Mr	3707.80	2082.19
Dx,g cm-3	3.297	3.702
Z	2	4
Mu (mm-1)	21.458	21.458
F000	3168.0	3168.0
F000'	3150.18	
h,k,lmax	20,25,22	19,25,22
Nref	3222	3066
Tmin,Tmax	0.008,0.117	0.008,0.117
Tmin'	0.001	

Correction method= # Reported T Limits: Tmin=0.008 Tmax=0.117
AbsCorr = MULTI-SCAN

Data completeness= 0.952   Theta(max)= 31.501

R(reflections)= 0.0267(2899)   wR2(reflections)= 0.0706(3066)

S = 1.087   Npar= 94

The following ALERTS were generated. Each ALERT has the format
test-name_ALERT_alert-type_alert-level.
Click on the hyperlinks for more details of the test.

[IMAGE] **Alert level G**

```
FORMU01_ALERT_2_G  There is a discrepancy between the atom counts in the
            _chemical_formula_sum and the formula from the _atom_site* data.
            Atom count from _chemical_formula_sum:C2 H38 Er1 N1 O38 Te1 W6
            Atom count from the _atom_site data:  Er1 O28.5 Te1 W6
CELLZ01_ALERT_1_G Difference between formula and atom_site contents detected.
CELLZ01_ALERT_1_G ALERT: Large difference may be due to a
            symmetry error - see SYMMG tests
           From the CIF: _cell_formula_units_Z    4
           From the CIF: _chemical_formula_sum  C2 H38 Er N O38 Te W6
           TEST: Compare cell contents of formula and atom_site data

           atom    Z*formula  cif sites diff
           C          8.00      0.00    8.00
           H        152.00      0.00  152.00
```

```
          Er         4.00      4.00    0.00
          N          4.00      0.00    4.00
          O        152.00    114.00   38.00
          Te         4.00      4.00    0.00
          W         24.00     24.00    0.00
PLAT003_ALERT_2_G Number of Uiso or Uij Restrained non-H Atoms ...          1 Report
PLAT012_ALERT_1_G N.O.K.   _shelx_res_checksum Found in CIF ......     Please Check
PLAT041_ALERT_1_G Calc. and Reported SumFormula    Strings  Differ     Please Check
PLAT045_ALERT_1_G Calculated and Reported Z Differ by a Factor ...       0.50 Check
PLAT066_ALERT_1_G Predicted and Reported Tmin&Tmax Range Identical          ? Check
PLAT083_ALERT_2_G SHELXL Second Parameter in WGHT  Unusually Large      50.27 Why ?
PLAT186_ALERT_4_G The CIF-Embedded .res File Contains ISOR Records          1 Report
PLAT300_ALERT_4_G Atom Site Occupancy of O9         Constrained at       0.25 Check
PLAT302_ALERT_4_G Anion/Solvent/Minor-Residue Disorder (Resd  2  )       100% Note
PLAT304_ALERT_4_G Non-Integer Number of Atoms in ..... (Resd  2  )       0.13 Check
PLAT311_ALERT_2_G Isolated Disordered Oxygen Atom (No H's ?) .....         O9 Check
PLAT606_ALERT_4_G Solvent Accessible VOID(S) in Structure ........          ! Info
PLAT794_ALERT_5_G Tentative Bond Valency for W1        (VI)      .       5.98 Info
PLAT794_ALERT_5_G Tentative Bond Valency for W2        (VI)      .       5.96 Info
PLAT794_ALERT_5_G Tentative Bond Valency for Er1       (III)     .       3.05 Info
PLAT794_ALERT_5_G Tentative Bond Valency for Te1       (VI)      .       5.83 Info
PLAT860_ALERT_3_G Number of Least-Squares Restraints .............          6 Note
PLAT868_ALERT_4_G ALERTS Due to the Use of _smtbx_masks Suppressed          ! Info
PLAT933_ALERT_2_G Number of OMIT Records in Embedded .res File ...          1 Note
```

---

   0 **ALERT level A** = Most likely a serious problem - resolve or explain
   0 **ALERT level B** = A potentially serious problem, consider carefully
   0 **ALERT level C** = Check. Ensure it is not caused by an omission or oversight
  22 **ALERT level G** = General information/check it is not something unexpected

```
   6 ALERT type 1 CIF construction/syntax error, inconsistent or missing data
   5 ALERT type 2 Indicator that the structure model may be wrong or deficient
   1 ALERT type 3 Indicator that the structure quality may be low
   6 ALERT type 4 Improvement, methodology, query or suggestion
   4 ALERT type 5 Informative message, check
```

Datablock: compound_3D_Tm

Bond precision:	Tm- O = 0.0050 A		Wavelength=0.71073
Cell:	a=13.7303(5)	b=17.5197(7)	c=15.6614(6)
	alpha=90	beta=90	gamma=90
Temperature:	296 K		

	Calculated	Reported
Volume	3767.4(2)	3767.4(2)
Space group	C c c m	C c c m
Hall group	-C 2 2c	-C 2 2c
Moiety formula	O56 Te2 Tm2 W12 [+ solvent]	?
Sum formula	O56 Te2 Tm2 W12 [+ solvent]	C4 H41 O36 N2 Te Tm W6
Mr	3695.14	2092.92
Dx,g cm-3	3.257	3.690
Z	2	4
Mu (mm-1)	21.401	21.402
F000	3156.0	3156.0
F000'	3138.02	
h,k,lmax	20,25,23	19,24,23
Nref	3258	3035
Tmin,Tmax	0.008,0.118	0.008,0.118
Tmin'	0.001	

Correction method= # Reported T Limits: Tmin=0.008 Tmax=0.118
AbsCorr = MULTI-SCAN

Data completeness= 0.932 Theta(max)= 31.546

R(reflections)= 0.0295(2796) wR2(reflections)= 0.0789(3035)

S = 1.103 Npar= 88

The following ALERTS were generated. Each ALERT has the format
test-name_ALERT_alert-type_alert-level.
Click on the hyperlinks for more details of the test.

[IMAGE] **Alert level G**

```
FORMU01_ALERT_2_G  There is a discrepancy between the atom counts in the
            _chemical_formula_sum and the formula from the _atom_site* data.
            Atom count from _chemical_formula_sum:C4 H41 N2 O36 Te1 Tm1 W6
            Atom count from the _atom_site data:  O28 Te1 Tm1 W6
CELLZ01_ALERT_1_G Difference between formula and atom_site contents detected.
CELLZ01_ALERT_1_G ALERT: Large difference may be due to a
            symmetry error - see SYMMG tests
           From the CIF: _cell_formula_units_Z    4
           From the CIF: _chemical_formula_sum  C4 H41 O36 N2 Te Tm W6
           TEST: Compare cell contents of formula and atom_site data

           atom    Z*formula  cif sites diff
           C         16.00      0.00   16.00
           H        164.00      0.00  164.00
           O        144.00    112.00   32.00
           N          8.00      0.00    8.00
           Te         4.00      4.00    0.00
           Tm         4.00      4.00    0.00
           W         24.00     24.00    0.00
```

```
PLAT003_ALERT_2_G Number of Uiso or Uij Restrained non-H Atoms ...          3 Report
PLAT012_ALERT_1_G N.O.K.   _shelx_res_checksum Found in CIF ......     Please Check
PLAT041_ALERT_1_G Calc. and Reported SumFormula    Strings  Differ     Please Check
PLAT045_ALERT_1_G Calculated and Reported Z Differ by a Factor ...       0.50 Check
PLAT066_ALERT_1_G Predicted and Reported Tmin&Tmax Range Identical          ? Check
PLAT083_ALERT_2_G SHELXL Second Parameter in WGHT  Unusually Large      64.00 Why ?
PLAT186_ALERT_4_G The CIF-Embedded .res File Contains ISOR Records          3 Report
PLAT606_ALERT_4_G Solvent Accessible VOID(S) in Structure ........          ! Info
PLAT794_ALERT_5_G Tentative Bond Valency for W1        (VI)      .       5.92 Info
PLAT794_ALERT_5_G Tentative Bond Valency for W2        (VI)      .       5.89 Info
PLAT794_ALERT_5_G Tentative Bond Valency for Tm1       (III)     .       3.13 Info
PLAT794_ALERT_5_G Tentative Bond Valency for Te1       (VI)      .       5.59 Info
PLAT860_ALERT_3_G Number of Least-Squares Restraints .............         18 Note
PLAT868_ALERT_4_G ALERTS Due to the Use of _smtbx_masks Suppressed          ! Info
PLAT933_ALERT_2_G Number of OMIT Records in Embedded .res File ...          6 Note
```

---

0 **ALERT level A** = Most likely a serious problem - resolve or explain
0 **ALERT level B** = A potentially serious problem, consider carefully
0 **ALERT level C** = Check. Ensure it is not caused by an omission or oversight
18 **ALERT level G** = General information/check it is not something unexpected

6 ALERT type 1 CIF construction/syntax error, inconsistent or missing data
4 ALERT type 2 Indicator that the structure model may be wrong or deficient
1 ALERT type 3 Indicator that the structure quality may be low
3 ALERT type 4 Improvement, methodology, query or suggestion
4 ALERT type 5 Informative message, check

Datablock: compound_3D_Yb

Bond precision:	Yb- O = 0.0115 A		Wavelength=0.71073
Cell:	a=13.5837(8)	b=17.4520(8)	c=15.5907(9)
	alpha=90	beta=90	gamma=90
Temperature:	296 K		

	Calculated	Reported
Volume	3696.0(3)	3696.0(3)
Space group	C c c m	C c c m
Hall group	-C 2 2c	-C 2 2c
Moiety formula	O56 Te2 W12 Yb2 [+ solvent]	?
Sum formula	O56 Te2 W12 Yb2 [+ solvent]	C2 H33 O35.5 N Te W6 Yb
Mr	3703.36	2042.95
Dx,g cm-3	3.328	3.671
Z	2	4
Mu (mm-1)	21.945	21.945
F000	3160.0	3160.0
F000’	3141.80	
h,k,lmax	19,25,22	19,25,22
Nref	3188	3020
Tmin,Tmax	0.007,0.111	0.007,0.111
Tmin’	0.001	

Correction method= # Reported T Limits: Tmin=0.007 Tmax=0.111
AbsCorr = MULTI-SCAN

Data completeness= 0.947 Theta(max)= 31.527

R(reflections)= 0.0666(2694) wR2(reflections)= 0.1743(3020)

S = 1.063 Npar= 88

The following ALERTS were generated. Each ALERT has the format
test-name_ALERT_alert-type_alert-level.
Click on the hyperlinks for more details of the test.

[IMAGE] **Alert level B**
PLAT097_ALERT_2_B Large Reported Max. (Positive) Residual Density 12.53 eA-3

[IMAGE] **Alert level C**
DIFMX02_ALERT_1_C The maximum difference density is > 0.1*ZMAX*0.75
The relevant atom site should be identified.
PLAT094_ALERT_2_C Ratio of Maximum / Minimum Residual Density .... 3.04 Report
PLAT242_ALERT_2_C Low 'MainMol' Ueq as Compared to Neighbors of Yb00 Check
PLAT250_ALERT_2_C Large U3/U1 Ratio for Average U(i,j) Tensor .... 3.2 Note

---

[IMAGE] **Alert level G**
FORMU01_ALERT_2_G There is a discrepancy between the atom counts in the
_chemical_formula_sum and the formula from the _atom_site* data.
Atom count from _chemical_formula_sum:C2 H33 N1 O35.5 Te1 W6 Yb1
Atom count from the _atom_site data: O28 Te1 W6 Yb1
CELLZ01_ALERT_1_G Difference between formula and atom_site contents detected.
CELLZ01_ALERT_1_G ALERT: Large difference may be due to a
symmetry error - see SYMMG tests

```
From the CIF: _cell_formula_units_Z    4
From the CIF: _chemical_formula_sum  C2 H33 O35.5 N Te W6 Yb
TEST: Compare cell contents of formula and atom_site data

atom    Z*formula  cif sites diff
C          8.00      0.00    8.00
H        132.00      0.00  132.00
O        142.00    112.00   30.00
N          4.00      0.00    4.00
Te         4.00      4.00    0.00
W         24.00     24.00    0.00
Yb         4.00      4.00    0.00
```

```
PLAT003_ALERT_2_G Number of Uiso or Uij Restrained non-H Atoms ...          2 Report
PLAT012_ALERT_1_G N.O.K.   _shelx_res_checksum Found in CIF ......     Please Check
PLAT041_ALERT_1_G Calc. and Reported SumFormula    Strings  Differ     Please Check
PLAT045_ALERT_1_G Calculated and Reported Z Differ by a Factor ...       0.50 Check
PLAT066_ALERT_1_G Predicted and Reported Tmin&Tmax Range Identical          ? Check
PLAT083_ALERT_2_G SHELXL Second Parameter in WGHT  Unusually Large     431.84 Why ?
PLAT186_ALERT_4_G The CIF-Embedded .res File Contains ISOR Records          2 Report
PLAT605_ALERT_4_G Largest Solvent Accessible VOID in the Structure       1557 A**3
PLAT720_ALERT_4_G Number of Unusual/Non-Standard Labels ..........         12 Note
PLAT794_ALERT_5_G Tentative Bond Valency for W002      (VI)      .       6.12 Info
PLAT794_ALERT_5_G Tentative Bond Valency for W003      (VI)      .       5.94 Info
PLAT794_ALERT_5_G Tentative Bond Valency for Yb00      (III)     .       2.96 Info
PLAT794_ALERT_5_G Tentative Bond Valency for Te04      (VI)      .       5.83 Info
PLAT860_ALERT_3_G Number of Least-Squares Restraints .............         12 Note
PLAT868_ALERT_4_G ALERTS Due to the Use of _smtbx_masks Suppressed          ! Info
```

---

0 **ALERT level A** = Most likely a serious problem - resolve or explain
1 **ALERT level B** = A potentially serious problem, consider carefully
4 **ALERT level C** = Check. Ensure it is not caused by an omission or oversight
18 **ALERT level G** = General information/check it is not something unexpected

7 ALERT type 1 CIF construction/syntax error, inconsistent or missing data
7 ALERT type 2 Indicator that the structure model may be wrong or deficient
1 ALERT type 3 Indicator that the structure quality may be low
4 ALERT type 4 Improvement, methodology, query or suggestion
4 ALERT type 5 Informative message, check

Datablock: compound_3D_Lu

```
Bond precision:  Te- O = 0.0097 A        Wavelength=0.71073

Cell:            a=13.5556(11)     b=17.4711(13)     c=15.5599(12)
                 alpha=90          beta=90           gamma=90
Temperature:     296 K
```

	Calculated	Reported
Volume	3685.1(5)	3685.1(5)
Space group	C c c m	C c c m
Hall group	-C 2 2c	-C 2 2c
Moiety formula	Lu2 O56 Te2 W12 [+ solvent]	?
Sum formula	Lu2 O56 Te2 W12 [+ solvent]	C3 H41.5 N1.5 Lu O38 Te W6
Mr	3707.22	2112.44
Dx,g cm-3	3.341	3.807
Z	2	4
Mu (mm-1)	22.151	22.151
F000	3164.0	3164.0
F000'	3145.51	
h,k,lmax	18,23,20	18,23,20
Nref	2400	2388
Tmin,Tmax	0.007,0.109	0.007,0.109
Tmin'	0.001	

Correction method= # Reported T Limits: Tmin=0.007 Tmax=0.109
AbsCorr = MULTI-SCAN

Data completeness= 0.995 Theta(max)= 28.361

R(reflections)= 0.0479(2383) wR2(reflections)= 0.1085(2388)

S = 1.258 Npar= 88

The following ALERTS were generated. Each ALERT has the format
test-name_ALERT_alert-type_alert-level.
Click on the hyperlinks for more details of the test.

[IMAGE] **Alert level C**

PLAT220_ALERT_2_C NonSolvent Resd 1 O Ueq(max)/Ueq(min) Range 5.7 Ratio

---

[IMAGE] **Alert level G**

```
FORMU01_ALERT_2_G  There is a discrepancy between the atom counts in the
            _chemical_formula_sum and the formula from the _atom_site* data.
            Atom count from _chemical_formula_sum:C3 H41.5 Lu1 N1.5 O38 Te1 W6
            Atom count from the _atom_site data:  Lu1 O28 Te1 W6
CELLZ01_ALERT_1_G Difference between formula and atom_site contents detected.
CELLZ01_ALERT_1_G ALERT: Large difference may be due to a
            symmetry error - see SYMMG tests
           From the CIF: _cell_formula_units_Z    4
           From the CIF: _chemical_formula_sum  C3 H41.5 N1.5 Lu O38 Te W6
           TEST: Compare cell contents of formula and atom_site data

           atom    Z*formula  cif sites diff
           C         12.00      0.00   12.00
           H        166.00      0.00  166.00
           N          6.00      0.00    6.00
```

```
          Lu         4.00      4.00    0.00
          O        152.00    112.00   40.00
          Te         4.00      4.00    0.00
          W         24.00     24.00    0.00
PLAT003_ALERT_2_G Number of Uiso or Uij Restrained non-H Atoms ...          8 Report
PLAT012_ALERT_1_G N.O.K.   _shelx_res_checksum Found in CIF ......     Please Check
PLAT041_ALERT_1_G Calc. and Reported SumFormula    Strings  Differ     Please Check
PLAT045_ALERT_1_G Calculated and Reported Z Differ by a Factor ...       0.50 Check
PLAT066_ALERT_1_G Predicted and Reported Tmin&Tmax Range Identical          ? Check
PLAT083_ALERT_2_G SHELXL Second Parameter in WGHT  Unusually Large     604.01 Why ?
PLAT178_ALERT_4_G The CIF-Embedded .res File Contains SIMU Records          1 Report
PLAT186_ALERT_4_G The CIF-Embedded .res File Contains ISOR Records          2 Report
PLAT605_ALERT_4_G Largest Solvent Accessible VOID in the Structure       1541 A**3
PLAT794_ALERT_5_G Tentative Bond Valency for W1        (VI)      .       5.94 Info
PLAT794_ALERT_5_G Tentative Bond Valency for W2        (VI)      .       5.94 Info
PLAT794_ALERT_5_G Tentative Bond Valency for Lu1       (III)     .       3.06 Info
PLAT794_ALERT_5_G Tentative Bond Valency for Te1       (VI)      .       5.89 Info
PLAT860_ALERT_3_G Number of Least-Squares Restraints .............         72 Note
PLAT868_ALERT_4_G ALERTS Due to the Use of _smtbx_masks Suppressed          ! Info
```

---

  0 **ALERT level A** = Most likely a serious problem - resolve or explain
  0 **ALERT level B** = A potentially serious problem, consider carefully
  1 **ALERT level C** = Check. Ensure it is not caused by an omission or oversight
 18 **ALERT level G** = General information/check it is not something unexpected

  6 ALERT type 1 CIF construction/syntax error, inconsistent or missing data
  4 ALERT type 2 Indicator that the structure model may be wrong or deficient
  1 ALERT type 3 Indicator that the structure quality may be low
  4 ALERT type 4 Improvement, methodology, query or suggestion
  4 ALERT type 5 Informative message, check

It is advisable to attempt to resolve as many as possible of the alerts in all categories. Often the minor alerts point to easily fixed oversights, errors and omissions in your CIF or refinement strategy, so attention to these fine details can be worthwhile. In order to resolve some of the more serious problems it may be necessary to carry out additional measurements or structure refinements. However, the purpose of your study may justify the reported deviations and the more serious of these should normally be commented upon in the discussion or experimental section of a paper or in the "special_details" fields of the CIF. checkCIF was carefully designed to identify outliers and unusual parameters, but every test has its limitations and alerts that are not important in a particular case may appear. Conversely, the absence of alerts does not guarantee there are no aspects of the results needing attention. It is up to the individual to critically assess their own results and, if necessary, seek expert advice.

Publication of your CIF in IUCr journals

A basic structural check has been run on your CIF. These basic checks will be run on all CIFs submitted for publication in IUCr journals (*Acta Crystallographica*, *Journal of Applied Crystallography*, *Journal of Synchrotron Radiation*); however, if you intend to submit to *Acta Crystallographica Section C* or *E* or *IUCrData*, you should make sure that full publication checks are run on the final version of your CIF prior to submission.

Publication of your CIF in other journals

Please refer to the *Notes for Authors* of the relevant journal for any special instructions relating to CIF submission.

PLATON version of 10/08/2020; check.def file version of 06/08/2020

Datablock compound_3D_Dy - ellipsoid plot

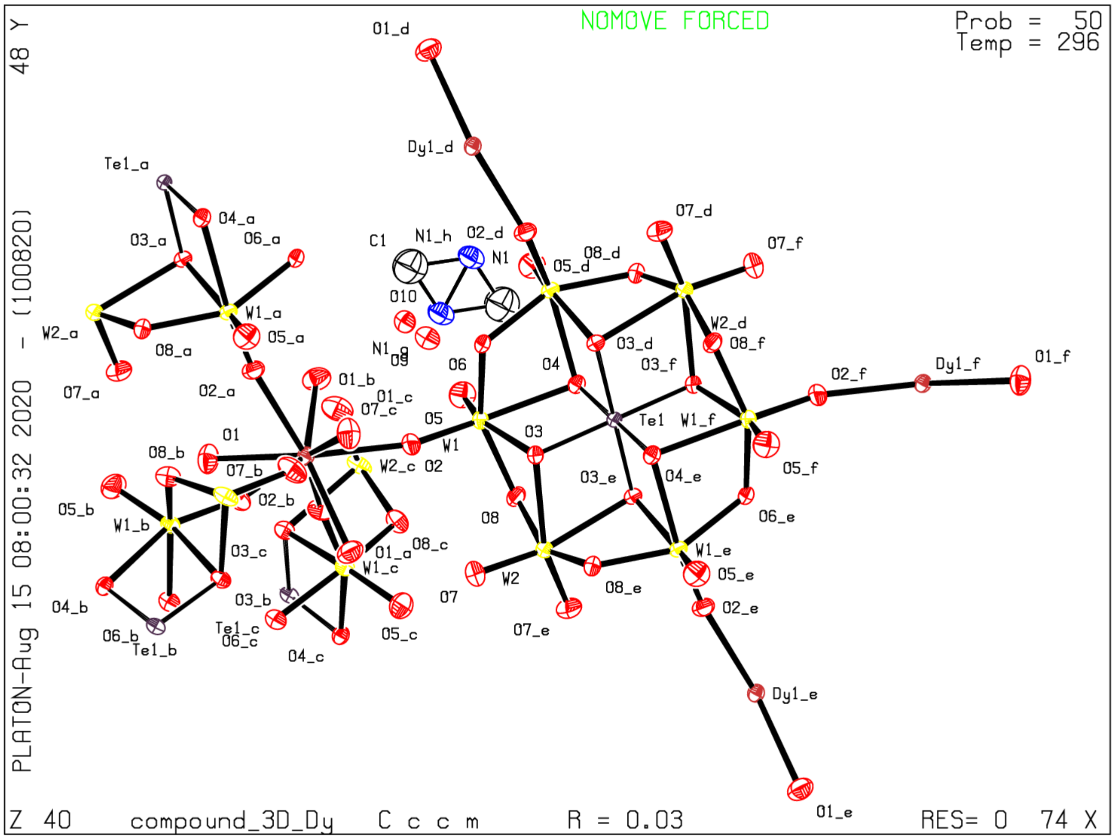

Datablock compound_3D_Ho - ellipsoid plot

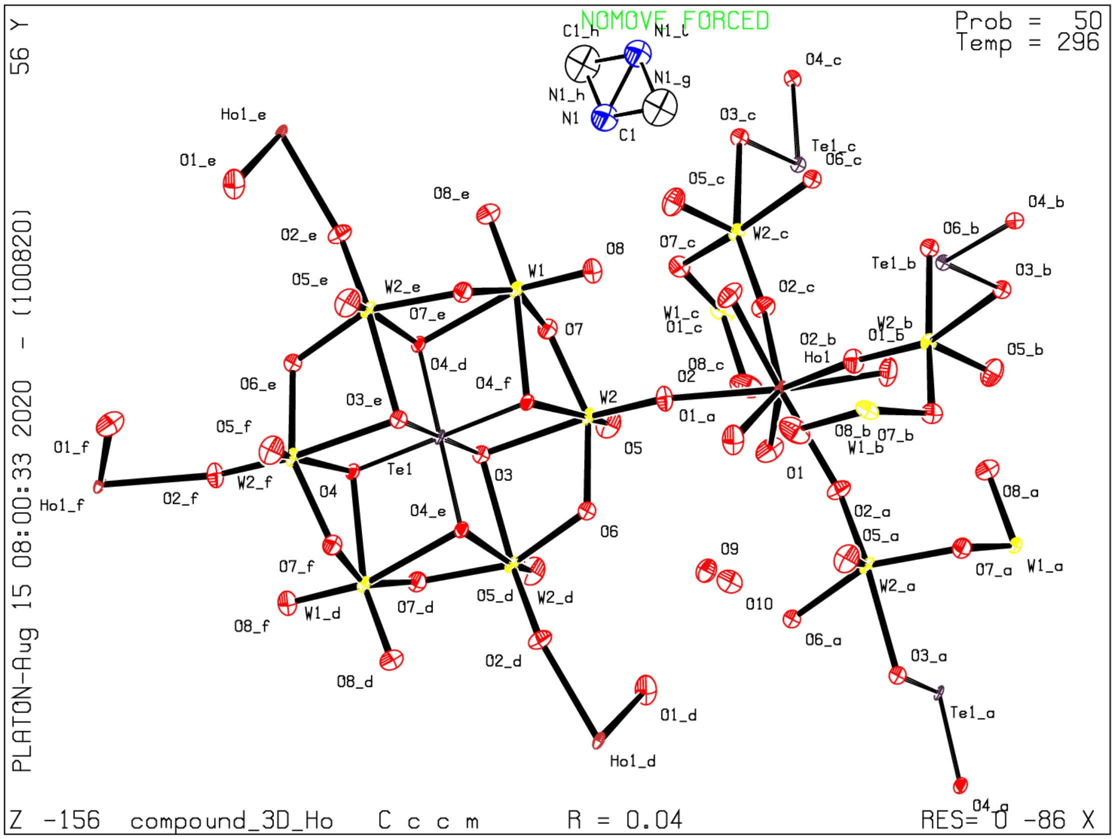

Datablock compound_3D_Er - ellipsoid plot

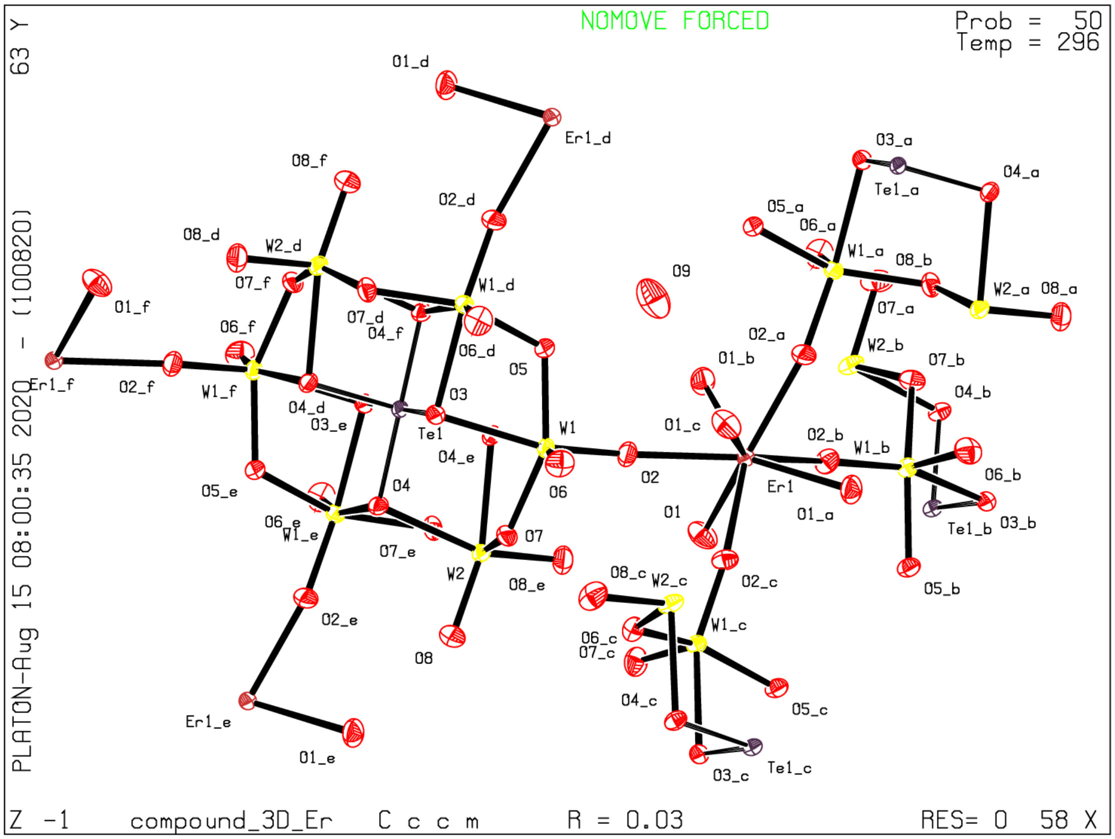

Datablock compound_3D_Tm - ellipsoid plot

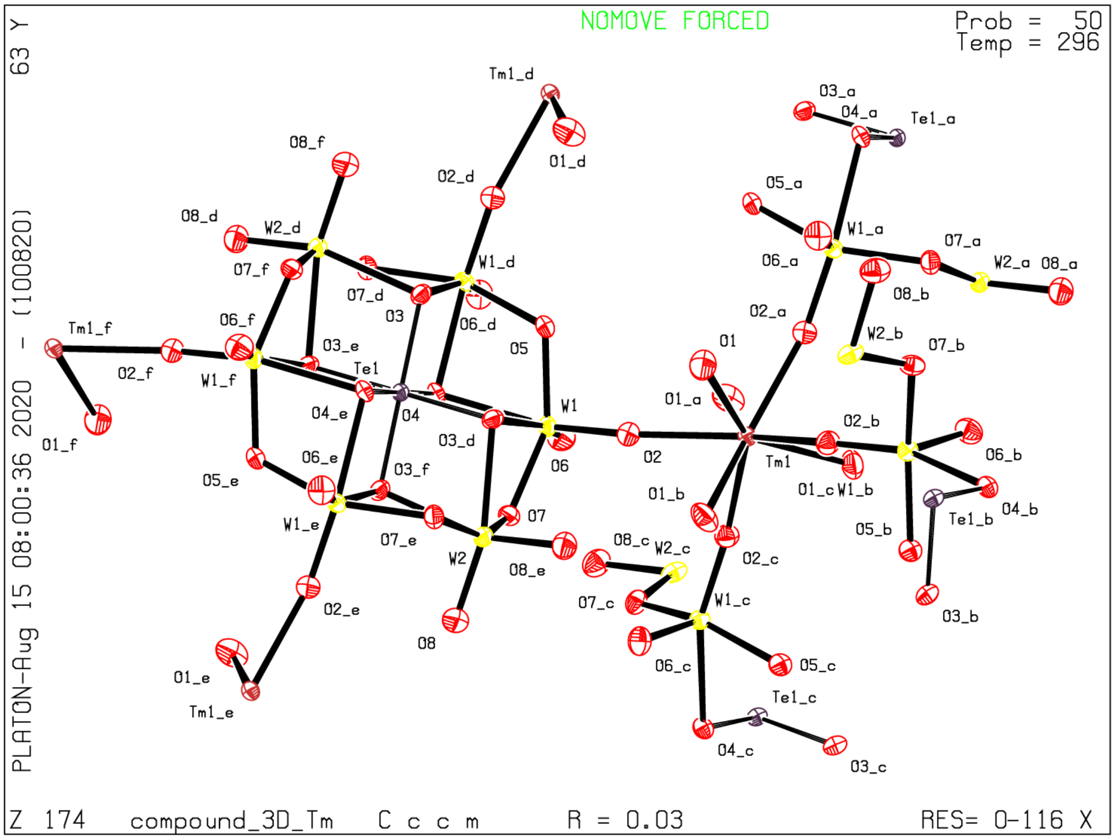

Datablock compound_3D_Yb - ellipsoid plot

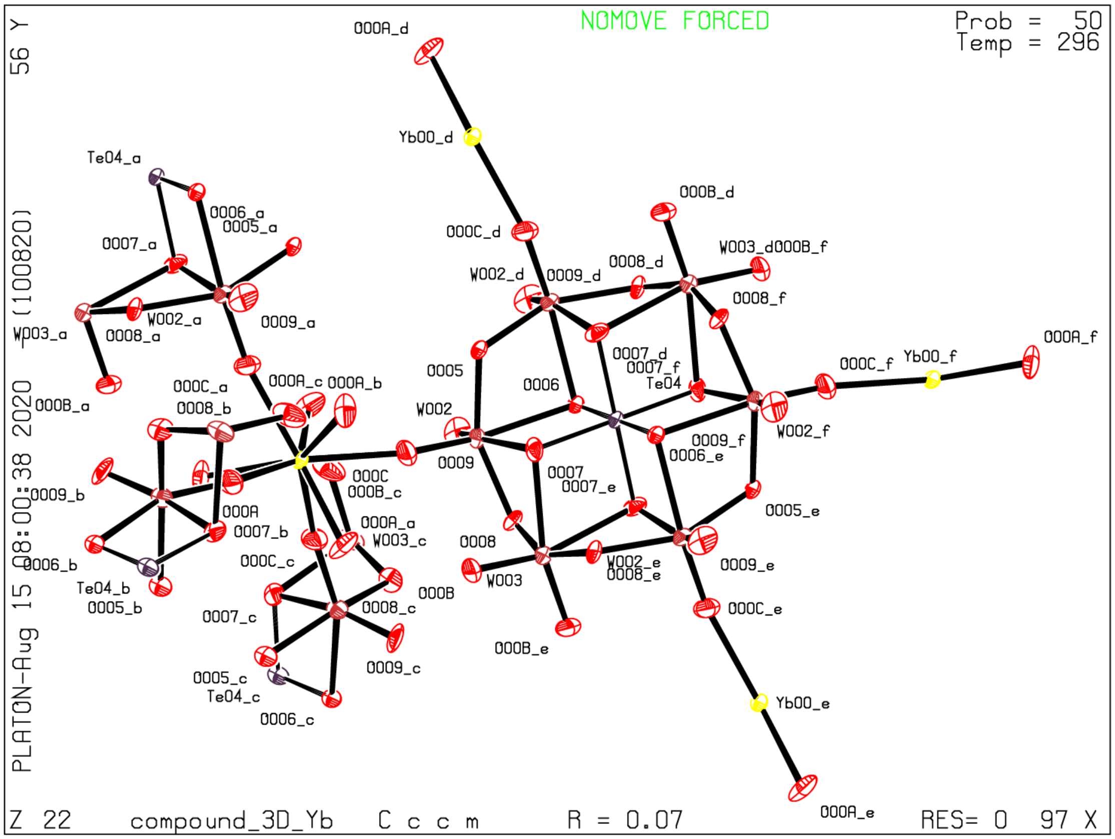

Datablock compound_3D_Lu - ellipsoid plot

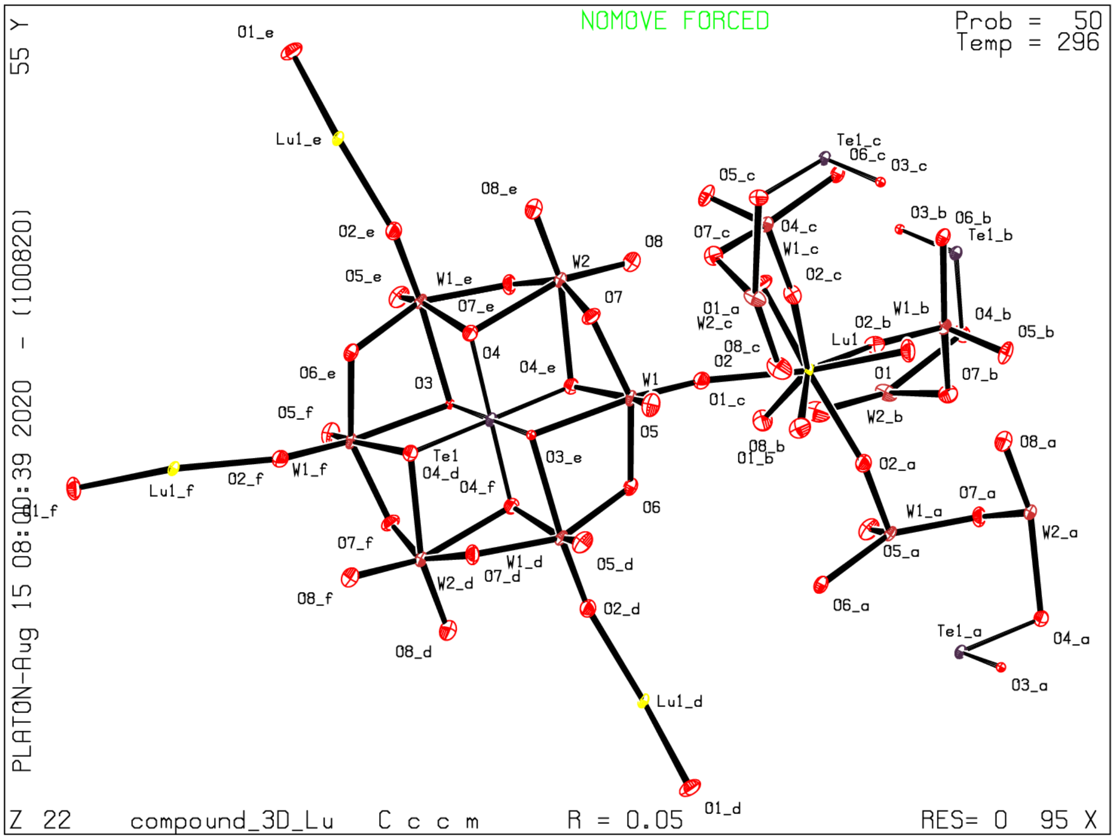

Supplement: Supplementary file 4 [file Data_Sheet_3.PDF]
